# Liver cancer cell lines distinctly mimic the metabolic gene expression pattern of the corresponding human tumours

**DOI:** 10.1186/s13046-018-0872-6

**Published:** 2018-09-03

**Authors:** Zeribe C. Nwosu, Nadia Battello, Melanie Rothley, Weronika Piorońska, Barbara Sitek, Matthias P. Ebert, Ute Hofmann, Jonathan Sleeman, Stefan Wölfl, Christoph Meyer, Dominik A. Megger, Steven Dooley

**Affiliations:** 1 Department of Medicine II, Molecular Hepatology Section, Medical Faculty Mannheim, Heidelberg University, Theodor-Kutzer-Ufer 1-3 (H42, Floor 4), 68167 Mannheim, Germany; 2 Molecular Hepatology Section, Medical Faculty Mannheim, Heidelberg University, Theodor-Kutzer-Ufer 1-3 (H42, Floor 4), 68167 Mannheim, Germany; 3 Luxembourg Science Center, 50 rue Emile Mark, L-4620 Differdange, Luxembourg; 4 Institut für Toxikologie und Genetik, Campus Nord, Karlsruhe Institute for Technology (KIT), Postfach 3640, 76021 Karlsruhe, Germany; 5 Medical Faculty Mannheim, CBTM TRIDOMUS-Gebäude Haus C, University of Heidelberg, 68167 Mannheim, Germany; 6 Medizinisches Proteom-Center, Department of Clinical Proteomics, Ruhr-Universität Bochum, Bochum, Germany; 7 Dr. Margarete Fischer-Bosch Institute of Clinical Pharmacology and University of Tübingen, 70376 Stuttgart, Germany; 8 Institute of Pharmacy and Molecular Biotechnology, Im Neuenheimer Feld 364, University of Heidelberg, 69120 Heidelberg, Germany; 9 Institute of Virology, University Hospital, University Duisburg-Essen, Essen, Germany

**Keywords:** Omics, Tumour metabolism, Well-differentiated, Poorly differentiated, Metabolic vulnerability, Selective therapy, Biomarkers, MAPK/Ras signaling, Hepatocellular carcinoma

## Abstract

**Background:**

Although metabolism is profoundly altered in human liver cancer, the extent to which experimental models, e.g. cell lines, mimic those alterations is unresolved. Here, we aimed to determine the resemblance of hepatocellular carcinoma (HCC) cell lines to human liver tumours, specifically in the expression of deregulated metabolic targets in clinical tissue samples.

**Methods:**

We compared the overall gene expression profile of poorly-differentiated (HLE, HLF, SNU-449) to well-differentiated (HUH7, HEPG2, HEP3B) HCC cell lines in three publicly available microarray datasets. Three thousand and eighty-five differentially expressed genes in ≥2 datasets (*P* < 0.05) were used for pathway enrichment and gene ontology (GO) analyses. Further, we compared the topmost gene expression, pathways, and GO from poorly differentiated cell lines to the pattern from four human HCC datasets (623 tumour tissues). In well- versus poorly differentiated cell lines, and in representative models HLE and HUH7 cells, we specifically assessed the expression pattern of 634 consistently deregulated metabolic genes in human HCC. These data were complemented by quantitative PCR, proteomics, metabolomics and assessment of response to thirteen metabolism-targeting compounds in HLE versus HUH7 cells.

**Results:**

We found that poorly-differentiated HCC cells display upregulated MAPK/RAS/NFkB signaling, focal adhesion, and downregulated complement/coagulation cascade, PPAR-signaling, among pathway alterations seen in clinical tumour datasets. In HLE cells, 148 downregulated metabolic genes in liver tumours also showed low gene/protein expression – notably in fatty acid β-oxidation (e.g. ACAA1/2, ACADSB, HADH), urea cycle (e.g. CPS1, ARG1, ASL), molecule transport (e.g. *SLC2A2, SLC7A1, SLC25A15/20*), and amino acid metabolism (e.g. PHGDH, PSAT1, GOT1, GLUD1). In contrast, HUH7 cells showed a higher expression of 98 metabolic targets upregulated in tumours (e.g. HK2, PKM, PSPH, GLUL, ASNS, and fatty acid synthesis enzymes ACLY, FASN). Metabolomics revealed that the genomic portrait of HLE cells co-exist with profound reliance on glutamine to fuel tricarboxylic acid cycle, whereas HUH7 cells use both glucose and glutamine. Targeting glutamine pathway selectively suppressed the proliferation of HLE cells.

**Conclusions:**

We report a yet unappreciated distinct expression pattern of clinically-relevant metabolic genes in HCC cell lines, which could enable the identification and therapeutic targeting of metabolic vulnerabilities at various liver cancer stages.

**Electronic supplementary material:**

The online version of this article (10.1186/s13046-018-0872-6) contains supplementary material, which is available to authorized users.

## Background

Hepatocellular carcinoma (HCC) is the most common type of liver cancer, which also include cholangiocarcinoma as well as secondary liver cancer that metastasized from distant organ-sites, e.g. colon and pancreas. HCC is a highly heterogeneous and poorly understood tumour entity. While a lot of its molecular alterations, e.g. *TP53*, *TERT* promoter and *CTNNB1* mutations are well known [1, 2], the clinical benefit of exploiting these genes has not been well proven. Similarly, reliable predictive biomarkers of  HCC are currently lacking. Drug resistance is also a major challenge in HCC, and has contributed to the failure of over 7 phase III clinical trials [3].

Similar to human HCC, the corresponding cell lines used for in vitro studies are heterogeneous in their molecular and phenotypic portraits. For example, HCC cell lines show differential proliferative response to Src/Abl inhibitor dasatinib [4] as well as transforming growth factor beta (TGF-β) stimulation [5, 6]. HCC cell lines also show dissimilar expression levels of many known cancer-associated proteins such as caveolin-1 (CAV1), alpha fetoprotein (AFP), and WNT signaling molecules [7–9]. Such distinct molecular and phenotypic background, which is also seen in cell lines of other cancer types, often raise the question of the extent to which cell lines mimic (or “recapitulate”) original human tumour profile. Although cancer cell lines are not necessarily original tumours – given the unphysiological culture conditions in which they are often maintained in vitro – numerous studies including the cancer cell line encyclopedia (CCLE) [10] and COSMIC projects [11] have found clinically meaningful similarities between cell lines and human tumours. In fact, despite the bourgeoning interest in the use of alternative models (e.g. organoids, mice), human cancer cell lines will for a long time remain the most readily accessible options for understanding the molecular basis of oncogenesis. Cell lines have notably proven to be useful for testing drug efficacy [10] and identifying synthetic lethality [12]. Therefore, a thorough characterization of the shared molecular signatures between HCC cell lines and the counterpart primary tumours is highly needed for defining core and novel alterations that can be investigated in vitro with the highest prospect of clinical translation.

We recently identified 284 metabolic genes upregulated in at least 6 of 8 human HCC microarray datasets, and also 350 downregulated metabolic genes under the same criteria. Two hundred and one of these genes were highlighted as predictive of overall survival in a cohort of HCC patients, underscoring the potential clinical significance of the genes [13]. Here, we investigated whether the expression pattern of those human HCC tissue-derived metabolic genes (herein called HMGs) is reflected in HCC cell lines, especially those poorly differentiated and known to be representative of more advanced HCC stage. By complementing the gene data with proteomics, metabolomics, and phenotypic response to metabolism-targeting drugs, we have uncovered pathway alterations that are shared or distinct between human HCC cell lines and the corresponding tumour  tissues.

## Methods

### Determination of the genomic pattern of human HCC tissues and cell lines

Microarray datasets GSE36133 (from CCLE project) [10], GSE35818 [4] as well as GSE57083 were used for comparing differential gene expression in human HCC cell lines. In each dataset, NCBI GEO2R tool was used to analyse the profile of HLE, HLF, and SNU-449 cells (poorly differentiated) relative to HUH7, HEPG2, and HEP3B cells (well-differentiated). Thereafter, the results were downloaded and the differentially expressed genes (*P* < 0.05) were separated into two categories – i.e. the upregulated and downregulated genes in poorly differentiated cell lines. Using Venn diagram tool (http://bioinfogp.cnb.csic.es/tools/venny/), genes exclusively upregulated or downregulated in ≥2 datasets (unless otherwise indicated) were selected for comparison with human HCC tissues genomic profile. Comparison with human HCC profile was in three parts. First, examining how the topmost 100 significantly upregulated or downregulated genes in all three cell line datasets are expressed in four patients datasets (GSE14520, GSE25097, GSE1898, and GSE55092). Second, examining the cell lines' expression of previously published list of consistent metabolic genes (*n* = 284 upregulated and 350 downregulated HMGs) that were derived from ≥6 human HCC microarrays, including GSE14520 (used in this study) [13]; and third, comparative analysis of the ontology of all genes consistently upregulated or downregulated in the four patients datasets (*P* < 0.05) and in ≥2 cell line datasets (*P* < 0.05). The second and third parts are discussed next. For the first part, the z-score of expression fold was calculated for each human HCC dataset. Then, the topmost genes identified from cell line data were extracted from each human dataset, and subsequently ranked by their average z-score across the four human datasets. With the + or – z-score values determined for each gene in all four human datasets, a gene significantly upregulated/downregulated in poorly differentiated HCC cell lines and upregulated/downregulated in the human datasets were considered to be concordantly expressed in both settings. In contrast, all genes significantly upregulated in poorly differentiated cell lines, but downregulated in tumours, and vice versa, were considered to be discordant (in other words, are more mimicked by well-differentiated HCC cell lines).

### Identification of altered human HCC metabolic genes mimicked by cell lines

To uncover the HMGs mimicked by the HCC cell lines, all genes significantly expressed in ≥2 of the earlier mentioned cell line datasets were overlapped with the 284 upregulated and 350 downregulated HMGs. This was followed by the selection of specific metabolic genes concordantly upregulated/downregulated in poorly differentiated cell lines as those more mimicked by the more ‘cancer-like’ cell lines. On the other hand, HMGs with discordant or opposite expression pattern were considered to be more portrayed by well-differentiated cell lines. In addition, HMGs expression was analyzed, focusing on HUH7 and HLE (two cell lines we mainly used for subsequent phenotypic assays). Specifically, gene expression data for these two cell lines were obtained via ArrayExpress database (https://www.ebi.ac.uk/arrayexpress/) for GSE57083, and cBioPortal platform for the CCLE project. Differences in measured mRNA (i.e. HLE - HUH7) were used to determine the relative expression of HMGs by the respective cell lines in each dataset. Subsequently, HLE cells were considered to mimic the up- or downregulated HMGs it concordantly express at a higher or lower level in both datasets. HMGs that showed discordant expression pattern in both datasets are more mimicked by HUH7 cells.

### Pathway enrichment and gene ontology analyses

Pathway enrichment and gene ontology (GO) analysis were performed using the bioinformatics platform DAVID 6.8 (https://david.ncifcrf.gov/). For human HCC microarrays, we have previously shown pathway enrichment [13] and so only GO analysis was performed in this current study. For this, the significantly upregulated (*n* = 2017) or downregulated (*n* = 1547) genes in all four human HCC datasets were separately analysed to determine their associated molecular function (MF), cellular component (CC) and biological process (BP). For the cell lines, pathway enrichment and GO analysis were performed using genes upregulated or downregulated in poorly differentiated HCC cell lines (at least in two datasets). Based on *P* < 0.0001 and false discovery rate (FDR) < 25%, the topmost 10 enriched pathways as well as top five MF, CC, and BP were selected.

### Cell culture

Human HCC cell lines HUH7, HEPG2, HLF and HLE were obtained from the Japanese Cancer Research Resources Bank or American Type Culture Collection. The cells were cultured at 37 °C in a humidified incubator with 5% CO_2_. Unless otherwise stated, cells were maintained in Dulbecco Modified Eagle’s medium (DMEM, High glucose, Lonza, BE12–709) supplemented with 2 mM glutamine (Gln), 10% heat inactivated fetal bovine serum, penicillin (100 U ml^− 1^), and streptomycin (100 μg ml^− 1^). For glucose or glutamine deprivation experiments, the cells were first seeded into culture plates and incubated overnight. Thereafter, the cells were washed 1× with Hank’s Balanced Salt Solution followed by further incubation with culture media lacking the respective metabolites. Compounds used to interfere with metabolism in the cell lines are listed in the Additional file 1: Methods.

### Proteomics

For proteomic comparison, HUH7 and HLE cells were seeded at a density of 5 × 10^5^ in 6 well plates (7 replicate wells for HUH7 and 8 for HLE). The cells were cultured for 48 h. Thereafter, culture media was aspirated off and the cells were washed by gentle swirling with 3 ml phosphate buffered saline per well. This step was repeated twice, after which cells were detached by scraping, and transferred into 1.5 ml vials. The cell suspension was then centrifuged at 800 rpm for 5 min at 4 °C and the supernatant discarded. The remaining cell pellets were stored at − 80 °C until analysis. Proteomic analysis was conducted by mass spectrometry as previously described [14]. Proteins with at least two unique peptides and FDR adjusted *P* < 0.05 after analysis of variance were considered significant in our analyses, which includes pathway enrichment, GO, and overlap with HMGs.

### Statistics and other analyses

Statistical significance was accepted as *P* < 0.05, unless otherwise indicated. Where applicable, results were expressed as mean ± standard deviation (SD). Data were analysed with GraphPad Prism v6 (La Jolla, USA). Genecards database (http://www.genecards.org) was used for crosschecking gene names and functions. The differentially expressed genes in human HCC microarrays GSE14520, GSE25097, GSE1898, and GSE55092 were determined by using NCBI GEO2R, comparing normal liver/adjacent non-tumours versus liver tumours. Information about metabolomics and other experiments performed with the cell lines are included in the Additional file 1: Methods.

## Results

### Poorly differentiated HCC cell lines are phenotypically more ‘cancer-like’ and possess tumour molecular portraits

**Fig. 1:**
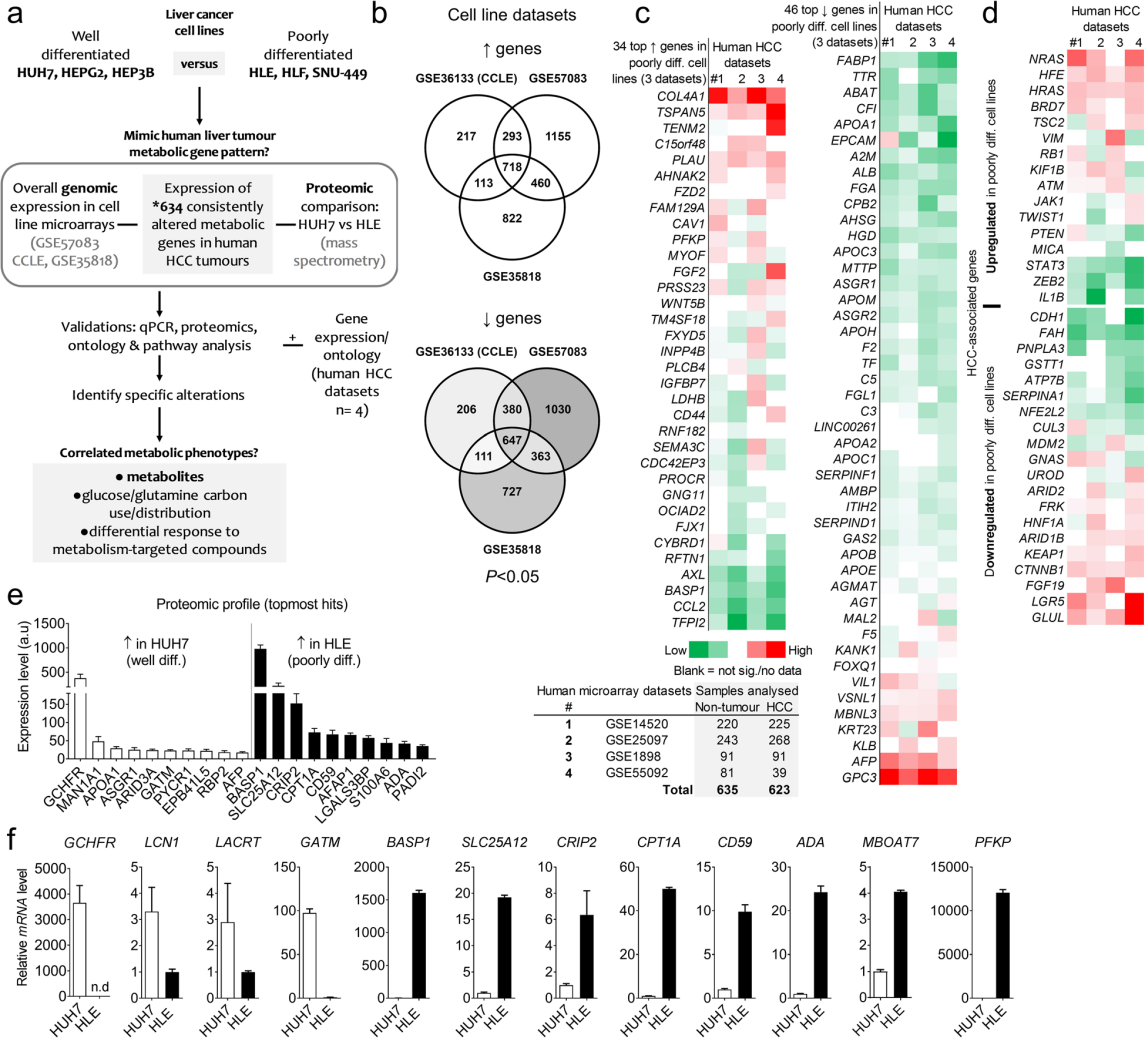
Molecular characterization of the resemblance between HCC cell lines and human liver tumours. **a** Workflow of analyses to unravel the metabolic profiles of HCC cell lines. *published list of metabolic genes consistently deregulated in 6–8 human HCC microarray datasets [13]. **b** Venn diagram showing the number of differentially expressed genes in poorly differentiated cell lines (HLE, HLF, SNU-449) relative to well-differentiated cell lines (HUH7, HEPG2, HEP3B), in three cell line microarray datasets (*P* < 0.05). **c** Heatmap showing genes within the top 100 upregulated or downregulated in all three cell line datasets, GSE36133, GSE35818, and GSE57083 (adjusted *P* < 0.0001) and their expression pattern in patients datasets (*P* < 0.05). Below are four human HCC datasets used for assessing resemblance with the cell line profile. The genes were ranked based on their average z-score in the human datasets. Diff. – differentiated. **d** Heatmap showing 36 HCC-associated genes differentially expressed in the poorly differentiated cell lines (*P* < 0.05 at least in one cell line dataset), and their expression pattern (z-score ranked) in patients datasets . Red – upregulated; green – downregulated; blank – not significant or no data available. **e** Top 10 proteins distinctly expressed in the representative well (HUH7) and poorly differentiated cell line (HLE). ≥2 peptides were detected by mass spectrometry for the indicated proteins, FDR adjusted *P* < 0.0001. Bars show mean ± SD of the expression of each protein in one of the cell lines compared to the other. Replicate samples *n* = 7 for HUH7 and 8 for HLE cells: a.u – arbitrary unit. **f** Quantitative PCR validation of a panel of genes derived from the topmost altered candidates in cell line datasets and or proteomics data. Bars show mean ± SD of triplicate samples

To identify human HCC metabolic gene patterns distinctly mimicked in vitro – the correlated proteomic and metabolite-level alterations, as well as cell-specific response to targeting metabolism – we started with comparing the genomic profile of six frequently used human HCC cell lines (Fig. 1a). Noteworthy, three of the cell lines (HUH7, HEPG2, and HEP3B) have epithelial features and represent early (well-differentiated) HCC stage, whereas the others (HLE, HLF, and SNU-449) are poorly differentiated late-stage models with mesenchymal cell properties [5, 9]. Importantly, these HCC cell lines are known to differ in cancer properties, with the poorly differentiated cells having higher proliferative and migratory phenotypes [4–9]. We confirmed that representative poorly differentiated cell line HLE has higher migration capacity compared to the well-differentiated cell HUH7 using FluoroBlok and in vitro scratch assays (Additional file 1: Figure S1a). Consistent with having more cancer features, intracellular adenosine triphosphate (ATP) – a metabolic readout positively associated with cell migration [15] – is also higher in poorly differentiated cell lines (Additional file 1: Figure S1b). To determine the genomic profile of the poorly differentiated relative to well-differentiated cell lines, we used three publicly available microarray datasets GSE57083, CCLE, and GSE35818 (Fig. 1a–d). We derived 3085 significantly expressed genes (*n* = 1584 upregulated and 1501 downregulated*, P* < 0.05) in at least two of the three datasets (Additional file 2: Table S1). Interestingly, within the top 100 upregulated, or downregulated genes in all three datasets were core candidates similarly upregulated (*n* = 34) or lowly expressed (*n* = 46, adjusted *P* < 0.0001) in poorly differentiated cell lines (Fig. 1c). Thus, with > 30% overlap within the top 100 genes, we considered these microarray datasets to be highly reproducible.

We therefore used the 34 upregulated and 46 downregulated genes to interrogate the resemblance in expression pattern between the cell lines and human HCC tissues from four datasets (total: 635 controls versus 623 tumour samples). Intriguingly, where significantly altered, more than half of the top upregulated genes in the cell lines (e.g. *RFTN1, TFPI2*, *AXL, BASP1,* C-C motif chemokine ligand 2*, CCL2)* were downregulated in most human HCC microarrays (Fig. 1c), suggesting a discordance in molecular expression in vitro for several upregulated genes in human HCC. Nevertheless, poorly differentiated cell lines mimicked upregulated expression of genes (in tumours) such as *CAV1, COL4A1,* and novel candidates such as *TSPAN5, TENM2*, *C15orf48, PLAU, AHNAK2, FAM129A, PLAU* and platelet-specific phosphofructokinase (*PFKP*) (Fig. 1c). In the downregulated gene category, human HCC profile was strongly mimicked by about 40 of the 46 identified genes (Fig. 1c), including fatty acid binding protein 1 (*FABP1*), *TTR, EPCAM,* apolipoproteins *(APOA1/2, APOB, APOC1/3, APOE), MTTP*, complement 3 and 5 (*C3/C5*), among other, reflecting ~ 85% concordance with patients’ tumour samples. The notable exemptions were villin 1 (*VIL1), VSNL1, MBNL3, KLB, AFP* and glypican 3 (*GPC3),* which are all consistently upregulated in liver tumour datasets*.* It is noteworthy that while many of these downregulated genes are novel candidates in HCC (Fig. 1c), AFP and GPC3 are often considered clinical biomarkers in HCC [16].

Next, we compiled a list of genes (*n* = 82, Additional file 3: Table S2) associated with human HCC. These, for instance, include candidate drivers, genes expressed by distinct proliferation subclasses or involved in epithelial–mesenchymal transition (EMT). Using this list, we asked which ‘HCC-associated’ genes can clearly distinguish poorly differentiated HCC cell lines from those well-differentiated, and also reflect a gene-level resemblance of the former to liver tumour tissues. Of the ‘HCC-associated’ gene list, 36 (44%) were differentially expressed (*P* < 0.05 at least in one dataset) in poorly differentiated cell lines relative to those well-differentiated. Examples are mitogen-activated protein kinase (MAPK)/RAS family members (*HRAS* and *NRAS*), hemochromatosis (*HFE)*, *BRD7*, *ATM*, vimentin (*VIM*), which are upregulated in poorly differentiated cell lines and in human HCC datasets (Fig. 1d). Other upregulated genes such as *PTEN, ZEB2, STAT3* and *IL1B* did not align with tumour expression pattern as they are consistently downregulated in the patients datasets.

Several ‘HCC-associated’ genes downregulated in poorly differentiated cell lines showed the opposite expression pattern in tumours. For example, besides e-cadherin (*CDH1*), *FAH, PNPLA3, GSTT1, ATP7B, SERPINA1*, *NFE2L2, CUL3* and *MDM2* (all downregulated both in poorly differentiated cell lines and tumours), the other ‘HCC-associated’ genes such as *FRK, HNF1A, ARID1B, KEAP1,* β-catenin *(CTNNB1), FGF19, LGR5* and glutamine synthetase (*GLUL)* were lowly expressed in poorly differentiated cell lines (i.e. more expressed in well-differentiated cells) and consistently upregulated in human liver tumours (Fig. 1d). Using HUH7 and HLE cell lines, we performed mass spectrometry-based proteomics and identified novel targets that clearly distinguish the two cell types (e.g. GCHFR, MAN1A1, APOA1, > 25 fold more expressed in HUH7 cells and BASP1, SLC25A12, CRIP2, CPT1A, CD59, AFAP1, LGALS3BP, > 50 fold more expressed in HLE cells at *P* < 0.0001) (Fig. 1e). The encoding genes of some of the proteins were validated by qPCR method (Fig. 1f), and showed a consistent expression pattern with the human tumour datasets (Additional file 1: Figure S1c). In immunoblotting and qPCR analyses, poorly differentiated cell lines also showed more resemblance to expected tumour profile based on their expression of known targets such as CDH1, VIM, CAV1, PKM2, pERK, mitochondrial pyruvate carrier 1 (MPC1), and matrix metalloproteinase 9 (Additional file 1: Figure S1d–e). Taken together, these data support that poorly differentiated HCC cell lines are more cancer-like in their molecular and phenotypic profiles.

### Molecular profiling reveals a strong overlap in metabolism between poorly differentiated HCC cell lines and human liver tumours

**Fig. 2:**
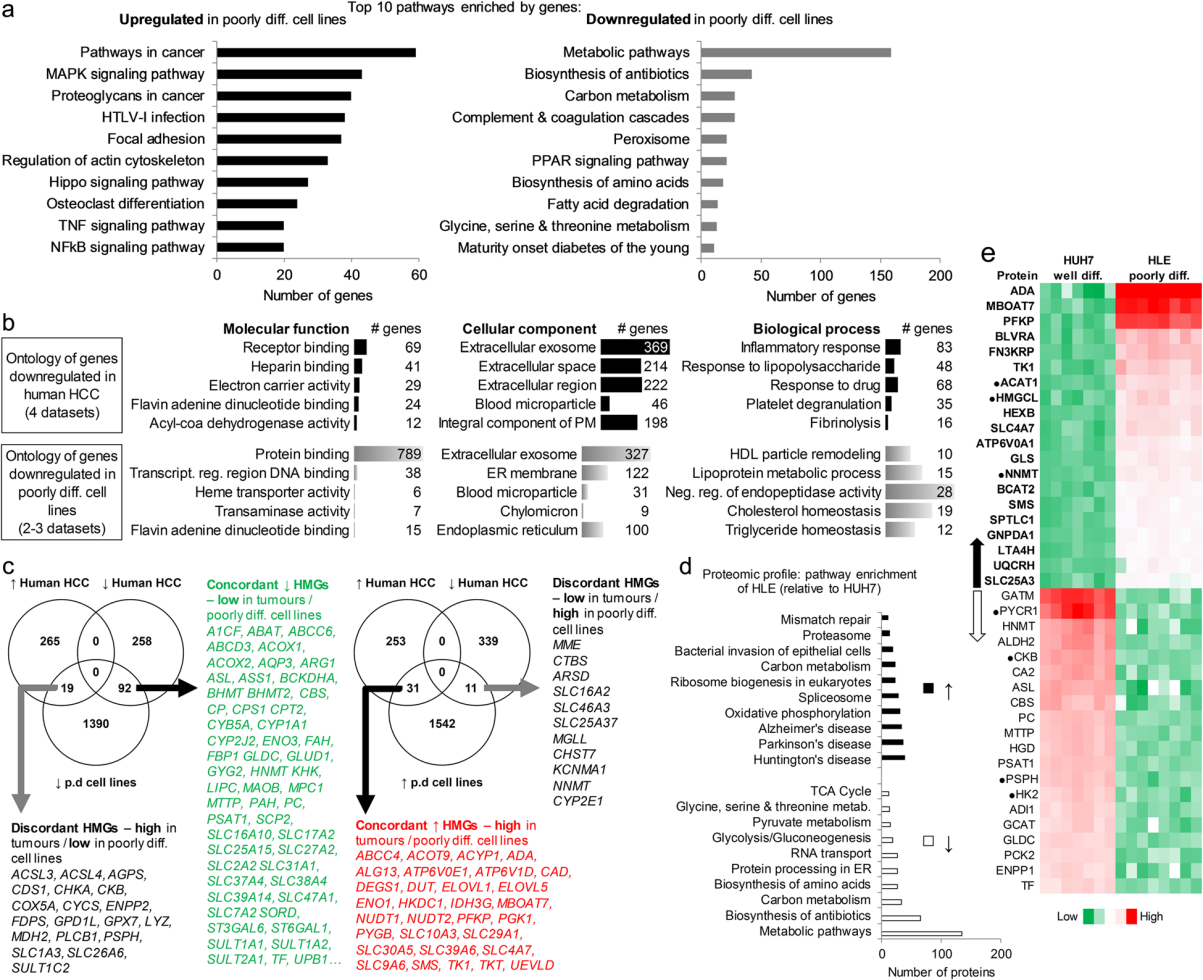
Functional annotation analysis, metabolic gene and proteomic profile of HCC cell lines. **a** Topmost pathway enrichment of genes upregulated (*n* = 1584) or lowly expressed (*n* = 1501) in poorly differentiated relative to well-differentiated HCC cell lines. *P* < 0.05 in 2–3 of the cell line datasets. Analysis was performed with DAVID v6.8, reference: Kyoto Encyclopedia of Genes and Genomes (KEGG). HTLV – Human T-lymphotropic virus, NFkB – nuclear factor kappa B, PPAR – peroxisome proliferator-activated receptor, TNF – tumor necrosis factor. **b** Topmost gene ontology (GO) for genes (*n* = 1547) consistently downregulated in all 4 human HCC microarrays datasets (GSE14520, GSE25097, GSE1898, GSE55092), compared to the GO profile of poorly differentiated cell lines. # - number of (genes). ER – Endoplasmic reticulum, HDL – high density lipoprotein, PM – plasma membrane, ‘Neg. – negative’ ‘Transcript.’ – transcriptional, ‘reg.’ – regulation. **c** Venn diagram showing human HCC tissue-derived metabolic genes (HMGs) expressed concordantly (i.e. mimicked) or discordantly by the poorly differentiated cell lines (2–3 datasets, *P* < 0.05). ↑ – upregulated; ↓ – downregulated. Note: the discordant genes are more mimicked by well-differentiated cell lines. A full list of the 92 concordant ↓ HMGs are contained in Additional file 6: Table S5. **d** Topmost pathway enrichment of proteins more expressed in HUH7 or HLE cells (FDR adjusted *P* < 0.05). **e** 20 most highly or lowly expressed HMGs-encoded proteins in the cell lines (FDR adjusted *P* < 0.05, mean fold difference > 2, HUH7 = 7, HLE = 8 samples). Arrows point to their encoding gene expression pattern in human HCC tissues. ● expressed in HCC in the opposite direction to the arrows

To identify the specific pathways to which the genes more or less expressed in the poorly differentiated HCC cell lines belong, we used the 1584 upregulated and 1501 downregulated genes for enrichment analysis in DAVID (https://david.ncifcrf.gov). Pathway enrichment of the upregulated genes indicated an activation of cancer pathways, MAPK/RAS pathway, focal adhesion, proteoglycans in cancer, nuclear factor kappa B signaling and tumour necrosis factor signaling (Fig. 2a). Examples of genes involved in these pathways are *CAV1/2, TGFB2, SMAD2/3, WNT5A/B, JUN, FGF1/3/5, MAPK10/11, HRAS, NRAS, RRAS*, *IRAK1,* etc (Additional file 4: Table S3a). This pathway enrichment pattern is consistent with our previous observation in eight human HCC microarray datasets[13]. Further, as observed in human HCC microarrays, the upregulation of cancer pathways in the poorly differentiated cell lines co-existed with the predominant downregulation of metabolic pathways – along with complement/coagulation cascade and peroxisome proliferator-activated receptor (PPAR) signaling (Fig. 2a, Additional file 4: Table S3b). However, our prior study did not analyse HCC gene ontology (GO) [i.e. molecular function, MF; cellular component, CC and biological process, BP]. Thus, to compare GO between the cell lines and tumours, we first separately overlapped upregulated or downregulated genes in the four human HCC datasets mentioned earlier  (Additional file 1: Figure S2a). This led to the generation of a list of core genes upregulated in all four human datasets  (e.g. *GPC3, AKR1B10, AFP, ACSL4, RRM2, CDK1, IGF2BP3, DKK1, TXNRD1*, *SOX4,* etc, *n* = 2017), as well as those consistently downregulated, e.g. liver-specific glutaminase (*GLS2*), complement 7 and 9 *(C7/9), CXCL12, CYP1A2, CYP2E1, GYS2, HAMP, IGF1, LCAT, SLC22A1,* etc, *n* = 1547) (Additional file 5: Table S4). Of note, *AKR1B10, ACSL4, GLS2*, *LCAT,* were among the metabolic targets we previously identified as consistent in HCC [13], indicating a high alteration frequency of these genes across liver cancer datasets.

GO of upregulated genes in human HCC or poorly differentiated cell lines showed substantial overlaps, i.e. common MF = 8, CC = 14, and 8 in BP (FDR < 25%). MF protein binding and cell adhesion-related activities, CC nucleoplasm and cytoplasm, as well as virus-related BP were within the top five of the respective GO terms for the human and cell line datasets (Additional file 1: Figure S2b). The GO derived with downregulated genes showed a much stronger concordance between tumours and the poorly differentiated cell lines (common MF = 13, CC = 19, and 38 in BP, FDR < 25%). Metabolic activities emerged as the most prominently mimicked alterations in the cell line and human HCC data (Fig. 2b). MF protein, fatty-acyl-CoA and flavin adenine dinucleotide binding, CC extracellular exosome and mitochondrial matrix, BP cholesterol metabolism and its transport activities (lipoprotein metabolic process) were the topmost or shared GOs (Fig. 2b). A striking BP in tumours that did not similarly manifest in poorly differentiated cell line data is ‘inflammatory response’, which may be due to the absence of tumour immune microenvironment in cell culture.

Next, we focused on metabolic genes. To our knowledge, the extent to which consistently altered metabolic genes in human HCC tissues (i.e. HMGs) are concordantly expressed by the surrogate cell lines has not yet been reported. We identified HMGs whose expression pattern are more mimicked by poorly differentiated HCC cell lines. These include  92 of 350 downregulated HMGs (Fig. 2c, see Additional file 6: Table S5). In addition, 31 of 284 upregulated HMGs showed higher expression in the poorly differentiated cell lines as expected, but we also found 30 HMGs with discordant expression pattern (Fig. 2c). Of note, the genes not mimicked by poorly differentiated cell lines show concordant pattern in well-differentiated cell lines as in human HCC microarrays. In addition, while poorly differentiated cell lines mimicked mostly the downregulated HMGs, the upregulated HMGs showed more consistency in well-differentiated cell lines. We further interrogated the proteomic data from HUH7 and HLE cells, focusing on proteins for which at least two peptides were detected and have FDR adjusted *P* < 0.05 (Additional file 7: Table S6). Pathway enrichment of the proteins more expressed in HLE cells (*n* = 797) was cancer-oriented albeit heterogeneous (Fig. 2d). In contrast, the proteins less expressed in HLE cells though lower in number (*n* = 616) were strongly involved in metabolic processes as observed with genomic data. Thus, at proteomic level, the poorly differentiated cell lines also strongly mimic the downregulation of metabolic components as seen in tumours. Carbon metabolism surprisingly appeared in the pathway enrichment of proteins more expressed as well as those lowly expressed in HLE cells (Fig. 2d). However, the specific proteins involved were a heterogeneous mixture of enzymes in glycolysis, tricarboxylic acid (TCA) cycle and amino acid metabolism (discussed latter).

In line with the notion that the metabolic gene profile of the cell lines is reflected at the protein level, the expression pattern of the top deregulated ‘metabolic proteins’ in HLE relative to HUH7 cells (fold difference > 2) overlapped with that of the encoding HMGs (Fig. 2e). For example, 17 of the top 20 upregulated metabolic proteins in HLE cells (except NNMT, HMGCL ACAT1), and 16 of the top 20 downregulated proteins (except HK2, PSPH, CKB, PYCR1) (Fig. 2e) are candidates whose corresponding HMGs are up- or downregulated, respectively. The exemptions, i.e. NNMT, HK2, etc., were expressed in HUH7 cells as in human HCC tissues, indicating that the well-differentiated cell lines also display tumour metabolic gene expression pattern at protein level (Additional file 1: Figure S3). Altogether, pathway enrichment, GO, and expression of specific metabolic genes or proteins underscore a strong overlap in signaling and metabolic processes between poorly differentiated HCC cell lines and clinical tumour data.

### HCC molecular alterations mimicked in the poorly differentiated cell lines include downregulated components of β-oxidation, urea cycle, molecule transport, and amino acid processes

**Fig. 3:**
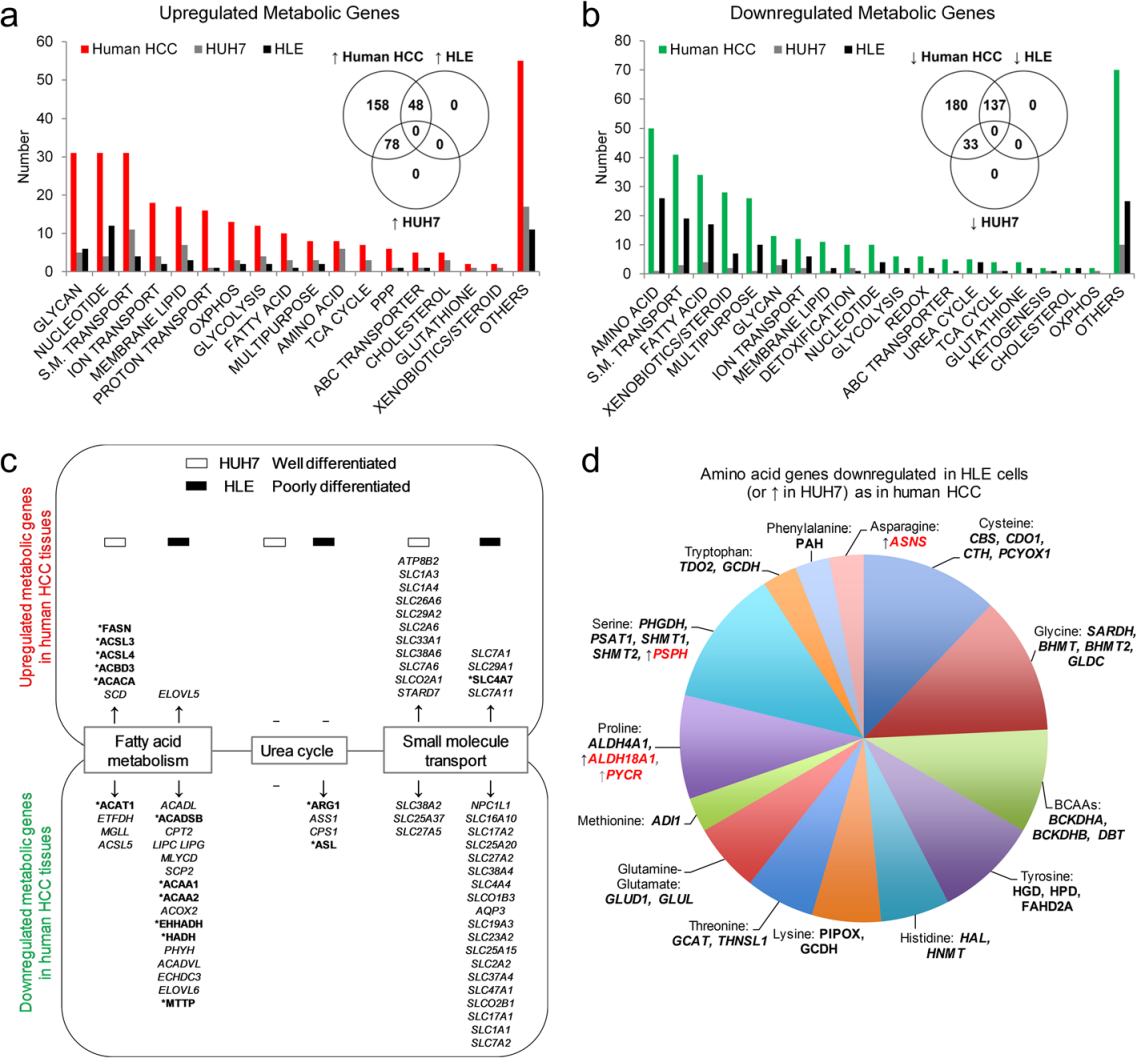
The collective representation of human HCC metabolic gene pattern by liver cancer cell lines. **a** Distribution of upregulated HMGs similarly more expressed in either HUH7 or HLE cells across metabolic processes. **b** Distribution of downregulated HMGs similarly less expressed in either HUH7 or HLE cells across metabolic processes. Generated based on consistent expression pattern in both GSE57083 and CCLE datasets.  ‘Others’ – metabolic processes beyond those shown. S.M. – small molecules, TCA – tricarboxylic acid, OXPHOS – oxidative phosphorylation, PPP – pentose phosphate pathway. **c** Specific HMGs more mimicked by HUH7 or HLE cell lines in selected metabolic processes . *showed similar expression pattern in proteomics. **d** Amino acid-associated HMGs that showed low expression in HLE cells, or high expression (↑/red) in HUH7, as in patients datasets. BCAAs – branched chain amino acids

It is noteworthy that some metabolic genes , though differentially expressed between well- and poorly differentiated cell lines, were at protein level not distinctly expressed in HLE compared to HUH7 cells. Examples are RRM1, RRM2, PAICS, ITPA, PPAT and GART (in nucleotide biosynthesis) and PGLS, TALDO1 and DERA in the pentose phosphate pathways (PPP). Along with these were over 20 candidates in ribosomal protein synthesis (Additional file 7: Table S6), suggesting that such indistinct expression level could be due to the overabundance of these proteins in the cell lines. To determine the specific metabolic processes represented by deregulated HMGs in HLE relative to HUH7 cells, we overlapped their distinctly expressed HMGs with those up- or downregulated in human HCC datasets (Fig. 3a–b). Consistent with the comparison of three well- versus poorly differentiated cell lines in general (shown in Fig. 2c), there was a strong overlap in the expression pattern of downregulated HMGs in HLE cells. Specifically, 137 downregulated HMGs  emerged  as also lowly expressed in HLE cells  (Fig. 3b). With upregulated HMGs, HUH7 cells showed a stronger resemblance to tumours as it expressed 78 candidates at a higher level compared to 48 candidates more expressed in HLE cells (Fig. 3a). Indeed, combined with proteomics data, 148 downregulated or 79 upregulated HMGs were similarly low or highly expressed by HLE cells, whereas 42 and 98, respectively, were expressed by HUH7 cells (Additional file 8: Table S7). These data confirm our earlier observation (shown in Fig. 1) that some genes identified in human HCC are more similarly expressed in the well-differentiated cell lines than in the more ‘cancer-like’ groups.

Next, we assigned the individual genes to their respective biochemical pathways, as in our prior study [13]. Across multiple metabolic pathways, several upregulated HMGs were collectively more expressed in HUH7 cells (Fig. 3a). For example, in glycolysis, hexokinase domain containing 1 (*HKDC1)*, hexokinase (HK1), *PFKP*, PGK1, ENO1, and lactate dehydrogenases (LDHA/B) are more expressed in HLE cells, but glucose transporter 6 (*SLC2A6*), hexokinase muscle isoform (*HK2)*, ALDOA, *BPGM*, pyruvate kinase (PKM), *DLAT*, and *MPC2* are upregulated HMGs more expressed in HUH7 cells. Similarly, asparagine synthetase (*ASNS)*, glutamine transporters (*SLC1A3*, *SLC1A4*), *FASN* in fatty acid synthesis (lipogenesis), and *PSPH* (in serine pathway) are all expressed higher in HUH7 cells. Furthermore, yet unknown but consistently upregulated HMGs such as *PLCB1, GNPAT, PLA2G7* and *CDS1* in membrane lipids, as well as *ALG3, B3GALNT1, CHPF2, DOLK* and *LYZ* in glycan metabolism are more expressed in HUH7 cells. Other upregulated metabolic genes that showed higher expression in HUH7 cells include NADPH oxidase 4 (*NOX4)*, phosphogluconate dehydrogenase (*PGD* in PPP*)* and *AKR1B10* (among the topmost upregulated metabolic gene in human HCC datasets), as well as key players in cholesterol metabolism (e.g. *SQLE, FDPS*, *IDI1*) and TCA cycle (e.g. *MDH2,* IDH1/2, IDH3A/B, FH, ME1/2) (Additional file 8: Table S7). These observations reveal known and novel metabolic genes in cancer that are more similarly expressed in a well-differentiated HCC cell line.

We observed an overwhelming consistency with human HCC data when considering downregulated HMGs also lowly expressed in HLE cells (Fig. 3b). Alterations in fatty acid metabolism (mainly β-oxidation), urea cycle, molecule transporters, and amino acid processes emerged as the most collectively represented in HLE cells. For instance, half of over 30 downregulated HMGs in fatty acid metabolism, e.g. ACAA1/2, ACADSB, HADH*, ACADL, CPT2* and lipases *(LIPC/G),* are low in HLE cells as in human HCC datasets (Fig. 3c). In urea cycle, of the 5 downregulated HMGs, four (*CPS1, ARG1, ASS1, ASL*) emerged as lowly expressed in HLE cells. This cell line also mostly mimicked downregulated small molecule transporters seen in tumours, e.g. *NPC1L1*, *SLC25A15/20, SLC2A2, SLC1A1* and *SLC7A2* (Fig. 3c). Indeed, besides urea cycle, downregulated amino acid HMGs were the best collectively represented alterations in HLE cells. We identified at least 31 of the ~ 50 downregulated amino acid genes in human HCC tissues that also showed low expression in HLE cells (Fig. 3d, Additional file 8: Table S7). The downregulated amino acid-related HMGs are associated with almost all known amino acids, including phenylalanine, serine/glycine, glutamate, cysteine and branched chain amino acids (BCAAs, i.e. valine, isoleucine and leucine) (Fig. 3d). Well known examples are phenylalanine hydroxylase (*PAH*), *PHGDH*, *PSAT1*, *GLUL*, glutamate dehydrogenase 1 (*GLUD1*), among others. Indeed, the corresponding proteins encoded by many downregulated HMGs showed low expression in HLE cells, thus reinforcing the gene-level data (Fig. 3c, Additional file 8: Table S7). Altogether, we reveal that poorly differentiated HCC cells mimic in a cohesive manner the downregulation of physiologic liver metabolic components as seen in the tumour counterpart, with a remarkable similarity in the downregulation of transporters, fatty acid β-oxidation, urea cycle, and amino acid-related genes.

### The dysregulated metabolic pathways correlate with altered glutamine utilization

**Fig. 4:**
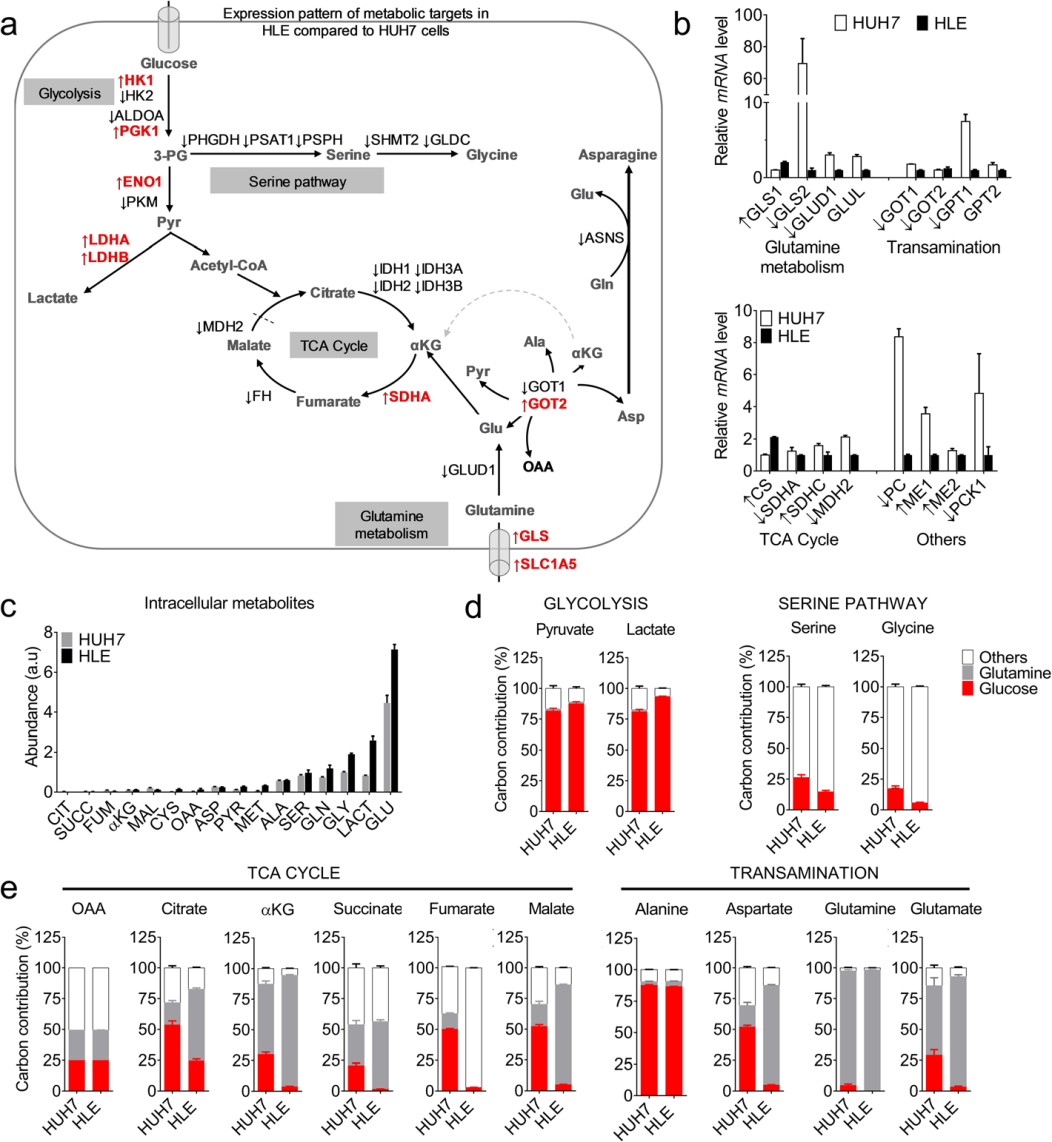
Metabolomic profile of the HCC cell lines. **a** Schematic representation of metabolic enzymes differentially expressed in intermediary pathways. In red and ↑ are those higher in HLE cells, while ↓ are lower compared to HUH7 cells. 3-PG – 3-phosphoglycerate; Pyr – pyruvate; OAA – oxaloacetate; αKG – alpha ketoglutarate; Asp – aspartate; Ala – alanine, Glu – glutamate; Gln – glutamine. **b** qPCR data showing relative mRNA level of notable metabolic genes in intermediary metabolism. Arrows indicate the direction of their consistent expression in human HCC microarray datasets as previously reported [13]. **c** Intracellular metabolites in HUH7 and HLE cells as measured after 24 h, in triplicates. Cit – citrate, Succ – succinate, Fum – fumarate, Mal – malate, Cys – cysteine, Ser – serine, Gly – glycine, Lact – lactate; a.u - arbitrary unit. **d** Isotope tracing data showing glucose/glutamine-derived carbon contribution to metabolites in glycolysis and serine pathway, after 24 h. **e** Isotope tracing data showing glucose/glutamine carbon contribution to TCA cycle and transamination metabolites after 24 h. Bars represent mean ± SD of triplicate analysis

We next wanted to uncover metabolite-level alterations that exist alongside the distinctly expressed metabolic genes or proteins in the HCC cell lines. To this end, we considered carbon metabolism, with emphasis on glycolytic pathway, TCA cycle and amino acid metabolism (Fig. 4a). Regarding glycolysis, both cell lines substantially mimic the expression pattern of key enzymes/transporters frequently altered in tumours (Fig. 4a), indicating that these cells employ different routes to preserve their glycolytic needs. On the contrary, as mentioned earlier, the downregulation of genes/proteins in amino acid metabolism was a more striking alteration in HLE cells consistent with human data. In perspective, besides higher expression of glutaminase (GLS) in HLE, this cell line showed a low expression of *GLS2, GLUD1, GLUL* in glutamine metabolism, PHGDH and serine hydroxymethyltransferase (SHMT1/2) in serine pathway, and transamination mediators (e.g. GOT1 and GPT1) as in patients’ datasets. Further, although HLE cells express a higher level of citrate synthase (CS), they lowly express MPC1 as noted earlier and also several TCA cycle enzymes as seen in patients datasets (Fig. 4a–b, Additional file 8: Table S7).

To gain clinically relevant insight on the overlap between carbon metabolism genes/enzymes and their associated metabolites, we next sort to determine metabolite-level changes that exist in human HCC tissues. For this, we referred to two tissue-based studies that together analysed 78 paired HCC samples [17, 18]. Interestingly, these studies demonstrated that the human HCC tissues have low glucose, elevated lactate [18], and low TCA cycle intermediates (e.g. succinate, fumarate and malate) [17, 18]. In contrast, human HCC tissues showed a high level of several amino acids, namely glutamine, glutamate, aspartate, methionine, serine, phenylalanine, tyrosine, and BCAAs [17, 18], indicating that altered amino acid genes/proteins also reflect at the metabolite level. Similar to human HCC tissues, HUH7 and HLE cells showed low intracellular level of TCA cycle intermediates compared to other metabolites like lactate, glutamine and glutamate (Fig. 4c). Compared to HUH7, HLE cells showed a higher level of glycolytic intermediate lactate (> 2 fold), as well as amino acids (e.g. methionine, glycine, glutamate and glutamine) (Fig. 4c), thus more closely portray the metabolite-level alterations seen in human HCC tissues.

Glucose and glutamine are two main metabolites known to be preferentially used by cancer cells [19]. Tracing their carbon distribution is a standard approach for elucidating intracellular metabolic activities in cancer [20]. We observed that glucose-derived carbon in glycolytic intermediates (pyruvate and lactate) is almost indistinct between both cell lines, albeit slightly higher in HLE cells (Fig. 4d). Serine – an amino acid produced de novo from glycolytic intermediate 3-phosphoglycerate [21, 22], and whose pathway enzymes are low in HLE cells (Fig. 4a) – displayed  a lower glucose-derived carbon in this cell line.  Similar readout was observed in glycine, which lie downstream of serine (Fig. 4d). We found striking differences between HUH7 and HLE cells when examining the contribution of glucose or glutamine-derived carbon to metabolites in the TCA cycle and transamination reaction. In HLE cells, the average glutamine carbon enrichment was about 80% for intermediates of TCA cycle [e.g. alpha ketoglutarate (αKG), malate] and transamination (glutamate and aspartate). This proportion is substantially higher compared to ~ 40% enrichment for the same metabolites in HUH7 cells (Fig. 4e). Citrate and succinate also showed ~ 2× higher glutamine-derived carbon enrichment in HLE than HUH7 cells (Fig. 4e), altogether suggesting a profound reliance on glutamine to sustain TCA cycle in the cell line more closely mimicking human HCC profile. Unlike glutamine, the glucose-carbon contribution to most TCA cycle metabolites is on average ~ 35% in HUH7 compared to ~ 5% in HLE (except citrate, ~ 25%). These data reveal that HUH7 cells support TCA cycle by proportionately using both glucose and glutamine, whereas altered amino acid processes in HLE cells align with a reliance on extracellular glutamine for enriching intermediates of TCA cycle, and aspartate in transamination pathway.

### Altered glutamine utilization is a selectively targetable metabolic vulnerability

**Fig. 5:**
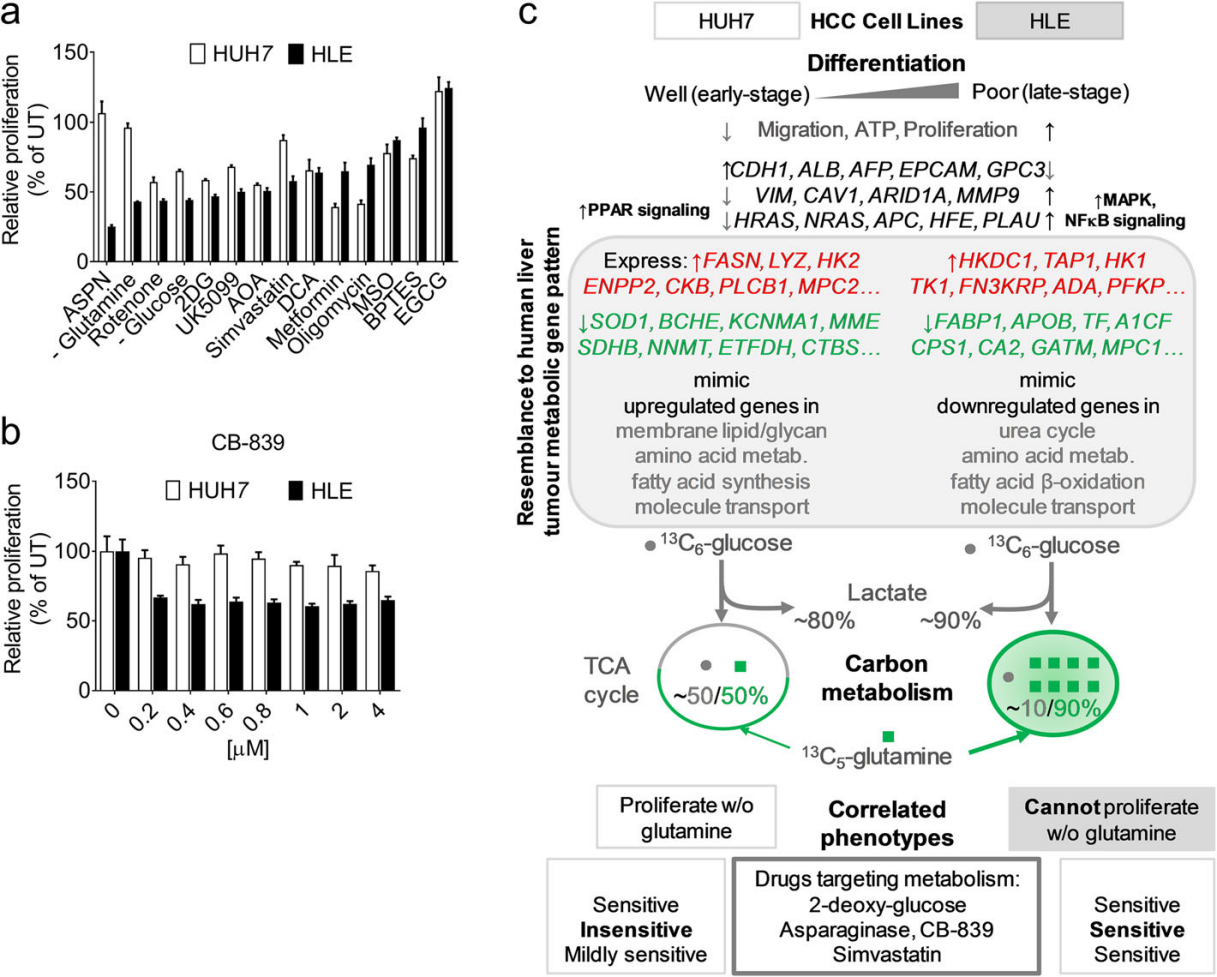
Phenotypic response to metabolic perturbation in HCC cell lines representing opposite tumour differentiation grades. **a** MTT proliferation assay showing response of the HCC cells to metabolism-targeting compounds or glucose/glutamine deprivation. ASPN – Asparaginase (0.5 U), Rotenone (0.25 μM), 2DG – 2-deoxy-glucose (5 mM), UK5099 (10 μM), AOA – Aminooxyacetate (1 mM), Simvastatin (2.5 mM), DCA –Dichloroacetate (5 mM), Metformin (5 mM), Oligomycin (1 μM), MSO – Methionine sulfoximine (2 mM), BPTES (5 μM) and EGCG (50 μM). Duration of treatment or deprivation is 48 h, except ASPN (72 h) and the cells were treated in parallel, each normalized to its untreated/control group. Bars indicate mean ± SD, and representative of at least 2 experimental observations (*n* = 4 per group). **b** MTT proliferation assay showing a selective antiproliferative effect of CB-839 (glutaminase inhibitor) on HLE cells over 48 h (*n* = 4 per group). CM – complete medium; UT – untreated, MTT – 3-(4,5-dimethylthiazol-2-yl)-2,5-diphenyltetrazolium bromide. **c** A scheme summarizing the differentiation status of HCC cell lines, examples of human HCC tissue-derived metabolic genes mimicked by the respective cell lines, the accompanying metabolomics profile, and differential response to metabolic perturbation. w/o – without: refers to extracellular glutamine

Targeting metabolism has shown promising prospects in cancer therapy [23–25]. We treated HUH7 and HLE cell lines in parallel with thirteen drugs acting on various metabolic pathways (Additional file 1: Figure S4a) to determine their selective proliferative response to metabolic perturbation. Of the tested drugs, metformin (complex I inhibitor), oligomycin (ATP synthase inhibitor), and BPTES (a glutaminase inhibitor) exerted more anti-proliferative effect on HUH7 than in HLE cells (Fig. 5a). Glucose deprivation, or treatment with 2-deoxy-glucose (2DG), suppressed the proliferation of both cell lines, although more profoundly in HLE cells as also observed in the other poorly differentiated cell line, HLF (Fig. 5a, Additional file 1: Figure S4b). Simvastatin (HMGCR inhibitor) and UK5099 (MPC inhibitor) both exerted more anti-proliferative effect on HLE compared to HUH7 cells. Treatment with methionine sulfoximine (MSO, inhibitor of GLUL), epigallocatechin gallate (EGCG, inhibitor of GLUD1), and aminooxyacetate (AOA, pan-transaminase inhibitor) did not produce a selective response in the cell lines (Fig. 5a).

Asparaginase is an enzyme clinically used for the treatment of acute myeloid leukemia [25], and was previously reported to deplete glutamine availability in HEPG2 cells [26]. Asparaginase selectively inhibited HLE cell proliferation with no tangible effect on HUH7 cells (Fig. 5a). Similarly, the withdrawal of extracellular glutamine (i.e. from culture media) caused a drastic and selective suppression of HLE cell proliferation (> 50% reduction in 48 h) and this effect was more obvious in the poorly differentiated- compared to the well-differentiated cell lines (Additional file 1: Figure S4c). We tested CB-839, a glutaminase inhibitor shown to mimic glutamine withdrawal in triple negative breast cancer cells [27]. As anticipated, CB-839 selectively inhibited HLE cell proliferation (Fig. 5b). These data show that the tumour molecular alterations displayed by the poorly differentiated cell lines (Fig. 5c) co-exist with inherent metabolic vulnerabilities like glutamine dependency that may lead to the identification of selectively druggable pathways in human liver cancer.

## Discussion

Recent studies have provided evidence of profound metabolic alterations in human liver cancer [13, 28–33]. HCC cell lines are among a plethora of preclinical experimental models (e.g. mice, organoids) that can enable basic and translational studies to delineate the relevance of altered metabolism, and the prospects of selective inhibition of biochemical processes in liver cancer. However, a critical but so far largely unanswered question is the extent to which experimental models mimic the metabolic features of liver tumours. In this study, we focused on the expression pattern of metabolic genes as it offers the advantage of broad pathway coverage, reflects tumour biochemical activities [34], and predicts clinical outcome in cancer datasets, HCC inclusive [13, 34]. Proteomics and metabolomics analyses were integrated to enable a multi-level insight on the tumour molecular alterations depicted by HCC cell lines. We placed greater emphasis on the poorly differentiated cell lines as they possess several known cancer phenotypes.

Our observation is that the cell lines representative of well (e.g. HUH7) and poorly differentiated (e.g. HLE) HCC subclasses, both, mimic the expression pattern of human HCC-derived metabolic genes (HMGs) (see summary in Fig. 5c). When compared, HLE cells strongly display a low expression of about 150 downregulated HMGs, notably involved in fatty acid β-oxidation, urea cycle, molecule transport, and amino acid metabolism. These alterations might be pivotal for HCC progression, given that this cell line possess more tumourigenic phenotype, e.g. higher migratory and proliferative capabilities. In addition, metabolic alterations in HLE cells correlate with other striking features, including a dependency on extracellular glutamine for proliferation, higher GLS expression, intracellular lactate, ATP, amino acids, as well as a higher sensitivity to metabolism-targeting drugs (e.g. asparaginase, CB-839, simvastatin). Of note, elevated lactate and amino acids have been shown in human HCC tissues [17, 18]. Further, profound downregulation of metabolic genes in HCC tissues, as seen in HLE cells, has been reported in other multi-omics data analyses studies, including TCGA liver cancer project [28, 29]. This downregulation of metabolic genes has been attributed to mutation or hypermethylation [29]. Strikingly, three specific metabolic genes implicated by the TCGA (i.e. *ALB, APOB, CPS1*) are all lowly expressed in HLE cells. Taken together, our findings on the metabolic profiles shared between tumours and the poorly differentiated cell lines support the notion that these cells could be important surrogates for clinically-relevant HCC metabolism studies.

While poorly differentiated cell lines more closely mimic human HCC profiles, the well-differentiated cells, exemplified by HUH7 cells, also showed striking resemblance to human tumours. For example, considering gene or protein expression differences, we identified almost 100 upregulated HMGs more expressed in HUH7 than in HLE cells. These upregulated HMGs are mostly involved in lipogenesis, small molecule transport, glycan-, membrane lipid- and cholesterol metabolism. Furthermore, with the exemption of GLS (more expressed in HLE cells), few amino acid-related genes upregulated in HCC tissues (e.g. *GLUL, ASNS*, *PSPH, ALDH18A1* and *PYCR1*) are more expressed in HUH7 cells. It is therefore difficult to exclude that the well-differentiated cell lines also display tumour molecular signatures. Rather, given that these cell lines are low migrating, low proliferating and are known to be more hepatocyte-like [4], we believe they indicate a breadth of novel metabolic alterations that either exist exclusively, or are initiated, at the early stages of liver cancer. At the metabolic phenotypic level, HUH7 cells do not rely on extracellular glutamine to drive their proliferation.

Warburg effect (i.e. aerobic glycolysis) is a known metabolic feature of cancer [25]. However, HLE and HUH7 cells did not clearly differ in their glucose carbon flux to glycolytic end-product lactate. Both cell lines are also sensitive to 2DG, which inhibits the first step in glycolysis, i.e. the hexokinase-catalysed phosphorylation of glucose to glucose-6-phosphate. Thus, it is noteworthy that both cell lines differentially express glycolytic enzymes (e.g. HK1 and LDHA/B are more expressed in HLE cells, whereas HK2 and PKM are higher in HUH7 cells). Such distinct expression of crucial enzymes  could enable an understanding of how human HCC fulfil glycolytic requirements at the early and advanced stages. Also noteworthy is a major distinction in the use glucose and glutamine for TCA cycle enrichment by the HCC cell lines. Whereas HUH7 cells take advantage of glucose and glutamine to fuel TCA cycle intermediates, HLE cells rely extensively on glutamine to support TCA cycle and aspartate. This data further supports the resemblance of HLE cells to tumours as glutamine dependency has been observed in in vitro and in vivo liver cancer studies [26, 35]. Consistent with our data,  prior studies have shown that glutamine sustains TCA cycle [23, 36, 37], and intracellular aspartate pool [38], with an overall phenotypic impact on cell proliferation. The support of aspartate production by glutamine is also biologically important in cellular respiration [39, 40]. Thus, we highlight altered glutamine metabolism as a metabolic vulnerability that could be exploited towards a selective treatment of HCC.

Beyond identifying resemblance to tumour metabolic profile, our study uncovered other altered targets in the cell lines that could be relevant signatures in HCC. For example, BASP1, LDHB, CD44 (high in HLE) and APOA1, ASGR1, ITIH2, AGT, AFP (high in HUH7 cells) emerged as the most consistently top targets (i.e. within top 100 genes in poorly differentiated cells, 3 datasets, and top 20 proteins in HLE relative to HUH7 cells). The impact of these candidates on HCC metabolic fate is currently  unclear. Of note, besides AFP and GPC3 which are highly studied biomarkers for early HCC detection [16], *CDH1, ALB* and *EPCAM* are highly expressed in HUH7 cells. This cell line also express a high level of many metabolic targets consistently upregulated in HCC patients microarrays (e.g. *AKR1B10, SQLE, LYZ, PLCB1*) [13], thus raising the question of whether HUH7 could be a crucial surrogate for identifying novel HCC metabolic biomarkers. On the other hand, higher expression of *HRAS, NRAS, APC, HFE, MMP9*, and a high enrichment of MAPK/RAS, NFKB, and TNF signaling pathways are all evident in HLE cells as often seen in cancer. Prospective hypotheses could interrogate the potential contribution of such enriched pathways in modulating HCC metabolism and progression.

## Conclusions

In light of the growing relevance of genomics in refining clinical HCC subclasses and therapeutic prospects [1, 2, 41], we identify for the first time, a set of human HCC-derived metabolic genes concordantly or discordantly expressed in vitro*.* Taken together with the metabolite level changes that coexist with these gene alterations, our findings provide useful insights that could lead to future identification of HCC subclasses with unique nutrient requirements. This will facilitate the prospects of targeting metabolism for HCC therapy, and pave way for delineating metabolic enzymes that are biomarkers or also indispensable drivers of HCC. Importantly, our work will guide the selection of suitable experimental models for the translational studies of specific metabolic pathway alterations seen in human liver cancer.

## Additional files

Additional file 1: Methods and Figures. **Figure S1.** Phenotypic and molecular characteristics of HCC cell lines. **Figure S2.** Overlap of genes deregulated in human HCC datasets, and the top ranked ontology of the highly expressed genes (tumours and poorly differentiated cell lines). **Figure S3.** Overlap of the gene and protein expression pattern in the cell lines compared with human HCC-tissue derived metabolic genes. **Figure S4.** Metabolic pathways targeted by the drugs used in this study and the effect of glycolysis inhibition or glutamine withdrawal on the proliferation of HCC cell lines. (PDF 1024 kb)

Additional file 2: **Table S1.** Genes more or lowly expressed in poorly differentiated relative to the well-differentiated cell lines. Includes all differentially expressed genes (i.e. in metabolism and other processes, *P* < 0.05). Datasets used: GSE57083, GSE36133 (CCLE) and GSE35818. (DOCX 166 kb)

Additional file 3: **Table S2.** HCC-associated genes. Contains a set of genes often associated with HCC, including those related to epithelial–mesenchymal transition. (DOCX 19 kb)

Additional file 4: **Table S3.** Top pathway enrichment and the specific genes involved. Enrichment analysis was performed with the genes more (3a) or (3b) lowly expressed in the poorly differentiated relative to well-differentiated HCC cell lines. (DOCX 18 kb)

Additional file 5: **Table S4.** Consistent genes in all four human HCC datasets analysed in this study. These are the genes differentially upregulated (in bold, *n* = 2017) or downregulated (*n* = 1547, *P* < 0.05) in HCC relative to non-tumour liver tissues in each of the four datasets. (XLSX 56 kb)

Additional file 6: **Table S5.** Complete list of downregulated HMGs concordantly low in the p.d. cells (part of Fig. 2c). (DOCX 14 kb)

Additional file 7: **Table S6.** Proteomics profile of HUH7 vs HLE cell lines. Expression pattern of proteins for which at least 2 peptides were detected in the cell lines after 48 h culture. When compared, 797 proteins emerged as more expressed in HLE cells whereas 616 proteins were more expressed in HUH7 (adjusted *P* < 0.05). Also included are 475 proteins indistinctly expressed under the same conditions. (XLSX 260 kb)

Additional file 8: **Table S7.** Human HCC tissue-derived metabolic genes (HMGs) mimicked by HLE or HUH7 cells upon comparison. Contains HMGs whose expression pattern in tumours (high or low expression) is reflected either in HLE (poorly differentiated, in total 227 HMGs) or HUH7 cells (well-differentiated, 140 candidates), when the cells are compared. The included HMGs showed consistent expression pattern in the respective cell lines both in GSE57083 and GSE36133 (CCLE) datasets. Those that also showed similar pattern at the protein level are indicated. (DOCX 25 kb)

## Abbreviations

2DG: 2-deoxy-glucose
ACLY: ATP citrate lyase
AFP: Alpha fetoprotein
ASNS: Asparagine synthetase
ATP: Adenosine triphosphate
BASP1: Brain abundant membrane attached signal protein 1
BCAAs: Branched chain amino acids
BP: Biological process
CAV1: Caveolin-1
CC: Cellular component
CCLE: Cancer Cell Line Encyclopaedia
CTNNB1: Beta-catenin
EMT: Epithelial–mesenchymal transition
FASN: Fatty acid synthase
FDR: False discovery rate
GLS: Glutaminase
GLUD: Glutamate dehydrogenase
GLUL: Glutamate-ammonia ligase (also called Glutamine synthetase)
GO: Gene ontology
GPC3: Glypican 3
HCC: Hepatocellular carcinoma
HK1/2: Hexokinase 1/2
HMGs: Human HCC tissue-derived metabolic genes
MAPK: Mitogen-activated protein kinase
MF: Molecular function
MPC1: Mitochondrial pyruvate carrier 1
NFKB: Nuclear factor kappa B
PPAR: Peroxisome proliferator-activated receptor
PPP: Pentose phosphate pathway
PSPH: Phosphoserine phosphatase
qPCR: Quantitative polymerase chain reaction
TCA: Tricarboxylic acid
TCGA: The cancer genome atlas
TGF-β: Transforming growth factor beta
αKG: Alpha ketoglutarate
