# Correction to: Liver cancer cell lines distinctly mimic the metabolic gene expression pattern of the corresponding human tumours

**DOI:** 10.1186/s13046-018-0939-4

**Published:** 2018-11-02

**Authors:** Zeribe C. Nwosu, Nadia Battello, Melanie Rothley, Weronika Piorońska, Barbara Sitek, Matthias P. Ebert, Ute Hofmann, Jonathan Sleeman, Stefan Wölfl, Christoph Meyer, Dominik A. Megger, Steven Dooley

**Affiliations:** 1 Department of Medicine II, Molecular Hepatology Section, Medical Faculty Mannheim, Heidelberg University, Theodor-Kutzer-Ufer 1-3 (H42, Floor 4), 68167 Mannheim, Germany; 2 Molecular Hepatology Section, Medical Faculty Mannheim, Heidelberg University, Theodor-Kutzer-Ufer 1-3 (H42, Floor 4), 68167 Mannheim, Germany; 3 Luxembourg Science Center, 50 rue Emile Mark, L-4620 Differdange, Luxembourg; 4 Institut für Toxikologie und Genetik, Campus Nord, Karlsruhe Institute for Technology (KIT), Postfach 3640, 76021 Karlsruhe, Germany; 5 Medical Faculty Mannheim, CBTM TRIDOMUS-Gebäude Haus C, University of Heidelberg, 68167 Mannheim, Germany; 6 Medizinisches Proteom-Center, Department of Clinical Proteomics, Ruhr-Universität Bochum, Bochum, Germany; 7 Dr. Margarete Fischer-Bosch Institute of Clinical Pharmacology and University of Tübingen, 70376 Stuttgart, Germany; 8 Institute of Pharmacy and Molecular Biotechnology, Im Neuenheimer Feld 364, University of Heidelberg, 69120 Heidelberg, Germany; 9 Institute of Virology, University Hospital, University Duisburg-Essen, Essen, Germany

## Correction

In the publication of this article, the concentration of CB-839 in Fig. 5b was inadvertently written as mM. This has now been updated to μM in the original article [1]. The authors declare that the correction does not change the results or conclusions of this paper.
