# Supplementary material for: Liver cancer cell lines distinctly mimic the metabolic gene expression pattern of the corresponding human tumours
Source: J Exp Clin Cancer Res. 2018 Sep 3;37:211. doi: 10.1186/s13046-018-0872-6 (PMC6122702; doi:10.1186/s13046-018-0872-6)
Supplement: Supplementary file 1 — Methods and Figures. Figure S1. Phenotypic and molecular characteristics of HCC cell lines. Figure S2. Overlap of genes deregulated in human HCC datasets, and the top ranked ontology of the highly expressed genes (tumours and poorly differentiated cell lines). Figure S3. Overlap of the gene and protein expression pattern in the cell lines compared with human HCC-tissue derived metabolic genes. Figure S4. Metabolic pathways targeted by the drugs used in this study and the effect of glycolysis inhibition or glutamine withdrawal on the proliferation of HCC cell lines. (PDF 1024 kb) [file 13046_2018_872_MOESM1_ESM.pdf]

Additional file 1: Methods and Figures

Liver cancer cell lines distinctly mimic the metabolic gene expression pattern of the corresponding human tumours

Nwosu et al.

Methods

Compounds used to target metabolism in the HCC cell lines

Name	Catalogue #	Mol. wt (g/mol)	Supplier
(−)-Epigallocatechin Gallate	E4143-50MG	458,37	Sigma
BPTES	SML0601-5mg	524,68	"
2-Deoxy-D-Glucose	D6134-1G	164,16	"
Aminooxyacetate (AOA)	C13408-1G	109,3	"
Asparaginase	A3809-100UN	-	"
Dichloroacetic acid	36545-1g	128,94	"
Metformin	Phr1084-500MG	165.62	"
Methionine sulfoximine	M5379-250MG	180.23	"
Simvastatin	S6196-5mg	418,57	"
UK5099	PZ0160-5MG	288.30	"
Oligomycin	75351-5MG	791.06	"
Rotenone	R8875	394.42	"
CB-839	22038	571.57	Bertin Pharma

RNA isolation and quantitative PCR

Total RNA was isolated using InviTrap Spin Universal RNA Mini Kit according to manufacturer's instruction (Stratec Biomedical AG, Germany). RNA concentration was quantified using Infinite 200 NanoQuant Plate (Tecan GmbH, Austria). Thereafter, RNA (0.5 or 1 µg) was reverse transcribed to cDNA using SuperScript II First Strand Kit (Invitrogen, USA). Quantitative PCR analysis was performed in a 10µl total reaction volume using SYBR Green PCR Master Mix on the Agilent Mx3005P QPCR Systems or AB StepOnePlus. Experiments were performed in triplicates with peptidylprolyl isomerase A (*PPIA*) as control and expression values calculated by delta CT method. The gene primer sequences used are listed below:

Gene primer sequences used for qPCR

Primer	Amplicon size	Forward	Reverse
CDH1	157	CTCTCACGCTGTGTCATCCA	CTCCATCACAGAGGTTCCTGG
MMP2	112	GATACCCCTTTGACGGTAAGGA	CCTTCTCCCAAGGTCCATAGC
MMP9	127	CGCTGGGCTTAGATCATTCCT	TCAGGGCGAGGACCATAGAG
PPIA	98	GACTGAGTGGTTGGATGGCA	TGCCATTCCTGGACCCAAAG
VIM	86	CACGTCTTGACCTTGAACGC	CTCCTGGATTTCCTCTTCGTGG
BASP1	90	TTCAGACTCAAAACCCGGCA	GCCTTGGGTGTGGAACTAGG
CRIP2	79	GTGCGACAAGACCGTGTACT	CTCGCACTTGAGGCAGAACT
CPT1A	73	GTACGCCAAGATCGACCCC	CATGCAGTTGGCCGTTTCC
CD59	88	CACAACCCGCTTGAGGGAA	CCCACCATTTTCAAGCTGTTCG
GCHFR	70	GCATCTGGGGGCTTCAAAGA	GGGAGGGTCATCGACGTAGT
ADA	78	TCCATCAAGCCTGAAACCATCT	CAGCCCCTCTGCTGTGTTAG
MBOAT7	83	GACCATCCGCAACATCGACT	GATATACTGCGCCAGCCACC
PFKP	96	GACCTTCGTTCTGGAGGTGATG	GATTCTGGAAGGAACACCCAGT
GATM	80	GGACTGCCCTGTCTCTTCTTAC	GAACACAGGCGTTTTCTGCTC
LCN1	73	CATGCTGATAAGTGGCCGGT	CGTGTATTTTCCCGGCTCGT
LACRT	139	CCCTGGTCTATGCTGAAGATGC	TGGGCTGTTGTGGTTGTCTC
SLC25A12	96	ACCCCAGAAGACTTTGTTCAGC	TTGATCAGCTACTCCTGCCAAG
MPC1	185	CATGAGTACGCACTTCTGGGG	GGCATGCAAACAGAAGCCAG
PDHA1	114	ATGGAATGGGAACGTCTGTTG	CCTCTCGGACGCACAGGATA
PC	115	GAGGTGAGATTGCCATCCGT	GCTTCATCTGCTTTCTGCCG
HK1	139	CCAACATTCGTAAGGTCCATTCC	CCTCGGACTCCATGTGAACATT
PGK1	137	GAACAAGGTTAAAGCCGAGCC	GTGGCAGATTGACTCCTACCA
LDHA	91	TTGACCTACGTGGCTTGGAAG	GGTAACGGAATCGGGCTGAAT
PKM2	108	ATAACGCCTACATGGAAAAGTGT	TAAGCCCATCATCCACGTAGA
CS	118	GCATGAGAGGCATGAAGGGA	CCCTTAGCCTTGGGTAGCAG
IDH1	129	CACCAAATGGCACCATACGAA	CCCCATAAGCATGACGACCTAT
SDHA	182	TGATGGGAACAAGAGGGCATC	ACCTGGTAGGAAACAGCTTGG
SDHC	176	TCCTCTGTCTCCCCACATTACT	CCAGACACAGGGACTTCACAA
ME1	85	TCTTGGCTTGGGAGACCTTG	ATTCATCCCTCCGCAAGCTG
ME2	177	CGACGGTTGGTCTTGCCTG	CCAGATCTCCAAGACCCAGAAT
PCK1	116	GCAAGACGGTTATCGTCACCC	GGCATTGAACGCTTTCTCAAAAT
GLS	114	AGGGTCTGTTACCTAGCTTGG	ACGTTCGCAATCCTGTAGATTT
GLS2	92	TCTCTTCCGAAAGTGTGTGAGC	CCGTGAACTCCTCAAAATCAGG
GLUD1	197	AAGGTGGCATGGTCCTCAAG	GGCATTCACTGTTTGAGTCAAGG
GLUL	137	TCATCTTGCATCGTGTGTGTG	CTTCAGACCATTCTCCTCCGG
GOT1	72	CAACTGGGATTGACCCAACT	GGAACAGAAACCGGTGCTT
GOT2	138	TTACGTTCTGCCTAGCGTCC	ACTTCGCTGTTCTCACCCAG
GPT1	75	GGTCTTGGCCCTCTGTGTTA	TCCGCCCTTTTCTTGGCATC
GPT2	103	GACCCCGACAACATCTACCTG	TCATCACACCTGTCCGTGACT
MDH2	134	GCAGCCACTTTCACTTCTCCT	CGAGGTAGCCTTTCACAGCG

Sources: *GOT1* - Son et al., 2013; *GLUL* - Chiu et al., 2014; *PCK1* - Foretz et al. 2010; PGK1 – Zieker et al., 2010; *PKM2* - Li et al., 2013; others - Harvard primer bank or designed with NCBI Primer Blast.

Intracellular metabolite profiling, glucose and glutamine isotope tracing

Sub-confluent HUH7 and HLE cells were each cultured overnight (o/n) in triplicates in 12-well plates. Next day, the culture medium was replaced with DMEM containing 10% dialysed Fetal bovine serum, 25 mM glucose and 4 mM Gln (Sigma-Aldrich). For isotope tracing, the DMEM was instead supplemented with 25mM uniformly labeled (U-$^{13}C_6$) glucose (Cambridge Isotope Laboratories) for glucose tracing, or 4 mM uniformly labeled (U-$^{13}C_5$) glutamine for glutamine tracing. The experiments were terminated after 24h. Extraction of intracellular metabolites, quantification by GC-MS and mass isotopomer distribution analysis was performed as previously described (Battello et al., 2016).

MTT proliferation assay

Cell proliferation was measured by 3-(4,5-dimethylthiazol-2-yl)-2,5-diphenyltetrazolium bromide (MTT) assay. Briefly, 2.5 – 7.5 x 10^3 cells per well were seeded in quadruplicates in 96-well plates and incubated o/n. The next day, the cells were treated with the indicated compounds. At the end of the experiment, MTT reagent was added to each well, followed by 3 – 4 h incubation at 37 °C. Media was then aspirated off and the formed formazan crystals were dissolved with 200 µl of solubilisation reagent (4 parts DMSO, 4 parts 10% SDS, 2 parts phosphate buffered saline, and acetic acid at 0.012% of the total reagent volume). Thereafter, the plate was incubated o/n at 37 °C. Absorbance was read at 560 nm using Infinite 200 Spectrophotometer (Tecan GmbH, Austria), with background correction at 670 nm. Experiments for HUH7 versus HLE were performed in parallel. Results were normalized to untreated/control group.

ATP determination assays

Cellular ATP content was measured using ATP Determination kit (Invitrogen, USA) according to manufacturer’s instruction. The results were normalized to total protein concentration of the cell lysates.

Western blotting

Following culture, adherent cells were washed, placed on ice, and cell protein lysates were prepared using radioimmunoprecipitation assay buffer supplemented with protease inhibitor

cocktail and phosphatase inhibitor. Protein content was estimated with Bio-Rad Protein Assay Kit according to manufacturer's instruction (Bio-Rad Laboratories Inc., USA). For electrophoresis, 20 – 25 µg of protein samples were loaded in 12% SDS–PAGE gels at 120V. Alternatively, 4-12% bis-Tris precast NuPAGE (Invitrogen, USA) was used at 150V for 75 minutes. Blots were subsequently transferred onto Nitrocellulose membranes (GE Healthcare Life Sciences) at 200 mA for 90 minutes. Thereafter, the membranes were briefly stained with Ponceau S solution to enable visualization of protein bands. Membranes with protein bands were then washed until Ponceau S stain was removed, using Tris-Buffered Saline containing 0.1% v/v Tween (TBST). The membranes were then blocked for 1h with 5% milk (dissolved in TBST), and later incubated o/n at 4°C with the primary antibody of interest. For detecting the bound protein, the corresponding mouse or rabbit secondary antibodies were diluted 1:10,000 and used to incubate the membrane for 1h incubation at room temperature, followed by 3x wash each for 10 minutes. The blot intensity was subsequently visualized after exposing the membrane to enhanced chemiluminescence (ECL) detection buffer. The following are the antibodies used:

Primary antibodies	Catalogue #	Source
Caveolin-1 (N20) (rabbit)	sc894	Santa Cruz Biotechnology
E-Cadherin (CDH1) (mouse)	3195S	Cell Signaling
MPC1 (D2L9I) (rabbit)	14462S	Cell Signaling
pERK (mouse)	sc-7383	Santa Cruz Biotechnology
PKM2	3198S	Cell Signaling
Tubulin (rabbit)	ab4074	Abcam
**Secondary antibodies**		
goat anti-mouse IgG-HRP	sc-2060	Santa Cruz Biotechnology
goat anti-rabbit IgG-HRP	sc-2301	Santa Cruz Biotechnology

Migration assays

For *in vitro* scratch assay, the cells were seeded in 24 well plates. After o/n incubation, the cell monolayer was scratched using 200µl pipette thus creating a gap. Culture media was replaced and bright field microscopy images were immediately taken (time point 0, t_0). After 24h, another image was taken (t_{24}). Using Image J, 20 equidistant measurements of the gap created by the scratch were taken from the t_0, and t_{24} images. Percentage migration was calculated as: 100 x (measured gap distance $t_0 – t_{24}$)/gap distance at t_0.

For FluoroBlok migration assay, harvested cells were pre-incubated for 15 minutes at 37°C with 15 µl DiI per ml of DMEM. Thereafter, the cells were washed twice with DMEM without phenol red. 1 x 10^5 HUH7 or 7.5 x 10^4 HLE cells were respectively seeded in 24-well plate in 200µl DMEM medium. The cells were seeded in the upper chamber of 8.0 µM Fluoroblok insert (Corning, USA), while 800µl of growth medium (i.e. with 10% FBS) was added to the lower chamber. For control plate, the same number of cells was seeded into a normal 24 well plate to serve as positive control (i.e. 100% migration). Triplicate wells containing only medium was also measured for background correction. Measurements were taken at 520/580 nm from the bottom of the plate using fluorescence reader. The whole experiment was performed in DMEM without phenol red. Calculation of % migration was as follows:

(measured value per experiment well – background)/(measured value of control plate – background)

Figure S1. Phenotypic and molecular characteristics of HCC cell lines

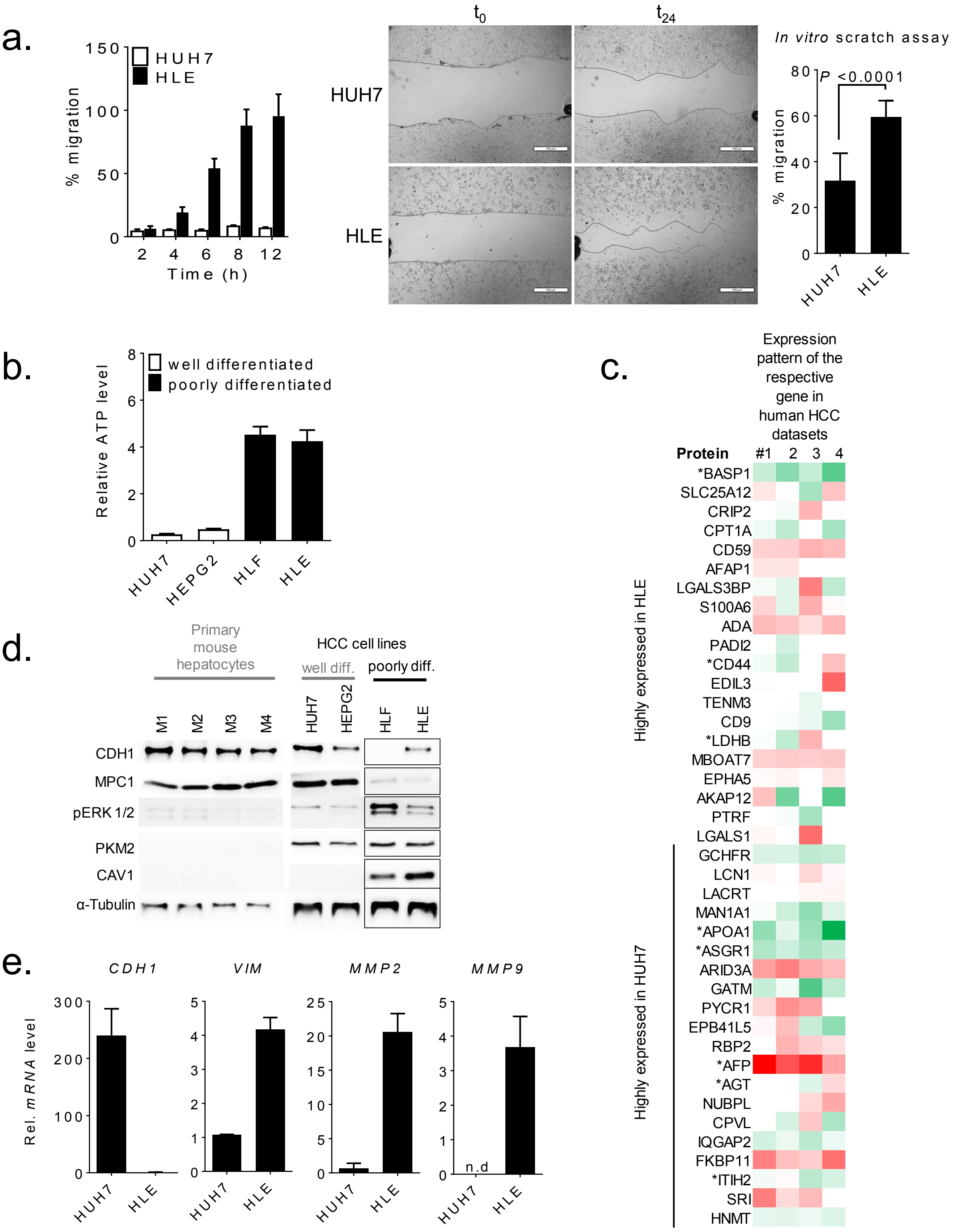

A. FluoroBlok migration assay, showing basal migration of HLE compared to HUH7 cells (n=3 per group). On the right, representative picture from *in vitro* scratch assay and the quantified gap distance showing % migration. Bars indicate mean ± SD of 20 measurements of migration after 24h (2 replicate experiments).
B. Relative intracellular ATP level determined after 24 h culture of the HCC cell lines. Bars indicate mean ± SD of triplicate samples.
C. Differentially expressed proteins (extended to top 20 high or low) in the cell lines and the expression pattern of their encoding genes in human HCC tumours ($P<0.0001$ except for LACRT and LCN1, $P<0.05$: * the corresponding genes for these candidates also appeared within the top 100 altered genes across all three cell line datasets (shown in Fig. 1c).
D. Western blot data showing differences between the HCC cell lines, and freshly isolated hepatocytes, using frequently altered proteins in cancer. M1 – 4 represent four mice from which hepatocytes were isolated as previously described (Dropmann et al., 2016).
E. qPCR showing relative mRNA level of EMT related genes E-cadherin (*CDH1*), vimentin (*VIM*) and matrix metalloproteinases 2 and 9 (*MMP2/*9) (n ≥ 2 per group). n.d. – not detected.

Figure S2. Overlap of genes deregulated in human HCC datasets and the top ranked ontology of the highly expressed genes (tumours and poorly differentiated cell lines)

a.

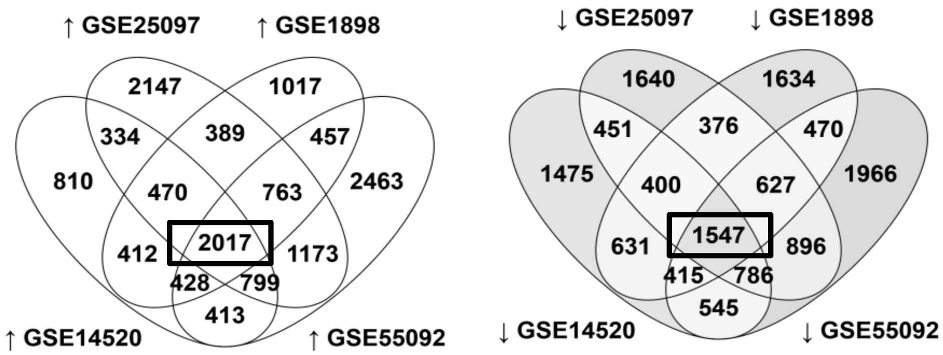

b.

Gene ontology of upregulated genes

Top 5 in Human HCC		Top 5 in poorly differentiated cell lines	
**Molecular function**	# genes	# genes	**Molecular function**
Protein binding	1388	840	Protein binding
Poly(A) RNA binding	259	7	S100 protein binding
ATP binding	266	87	Identical protein binding
Chromatin binding	94	20	Integrin binding
Cadherin binding involved in cell-cell adhesion	73	39	Actin binding
**Cellular component**			**Cellular component**
Nucleoplasm	645	522	Cytoplasm
Cytosol	605	356	Cytosol
Nucleus	848	68	Focal adhesion
Cytoplasm	804	34	Lamellipodium
Membrane	398	59	Cytoskeleton
**Biological function**			**Biological process**
Cell division	106	35	Cilium morphogenesis
DNA replication	60	31	Cilium assembly
Viral process	85	70	Positive regulation of transcription, DNA-templated
DNA repair	69	133	Signal transduction
Mitotic nuclear division	71	30	Defense response to virus

A. Venn diagram result showing the number of upregulated or downregulated genes in all 4 human HCC microarray datasets.
B. Ontology of the upregulated genes in the HCC microarrays compared to the poorly differentiated cell lines profile.

Figure S3. Overlap of the gene and protein expression pattern in the cell lines compared with human HCC-tissue derived metabolic genes.

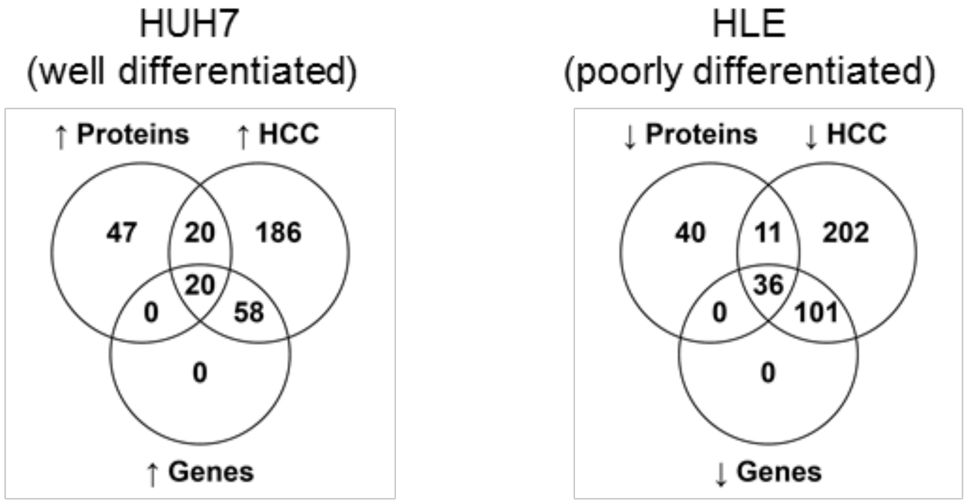

High gene/protein (HUH7) and high gene HCC = 20
ATP1B1, SEPHS1, HK2, ME1, ACBD3, PYCR1
IDI1, PSPH, NANS, ASNS, FDPS, PFAS, DLAT
AGPS, ACSL3, CKB, PGD, ALDH18A1, GFPT1, MDH2

High protein (HUH7) and high gene HCC = 20
IDH3B, DDAH2, BCAT1, ADSS, ADSL, SRM
NQO1, ABCF1, TXNRD1, PRDX1, ACACA
GPI, ALDOA, ACSL4, UGDH, ATIC, GAPDH, ACLY
PKM, FASN

Low gene/protein (HLE) and low gene HCC = 36
EHHADH, ASL, AGL, ADI1, HNMT, ALDH1B1
GCAT, SULT2A1, SLC39A14, GRHPR, NNT
GAMT, CA2, CBS, QDPR, ENPP1, ARG1
HADH, SARDH, ABHD10, ABCD3, ACADSB, CPOX
GATM, ACAA1, A1CF, UGP2, ACAA2, GLDC
HGD, ALDH2, GLUD1, MTTP, PSAT1, TF, PC

Low protein (HLE) and low gene HCC = 11
QPRT, DAK, PCK2, PAH, ACO1, CAT, SORD
ACOX1, GOT1, PGM1, PHGDH

Venn diagram showing the number of differentially expreesed metabolic genes in HLE relative to HUH7 cells (*P*<0.05) that their expression pattern (i.e. up- or down) was confirmed at protein level (proteomics).

Figure S4. Metabolic pathways targeted by the drugs used in this study and the effect of glycolysis inhibition or glutamine withdrawal on the proliferation of HCC cell lines.

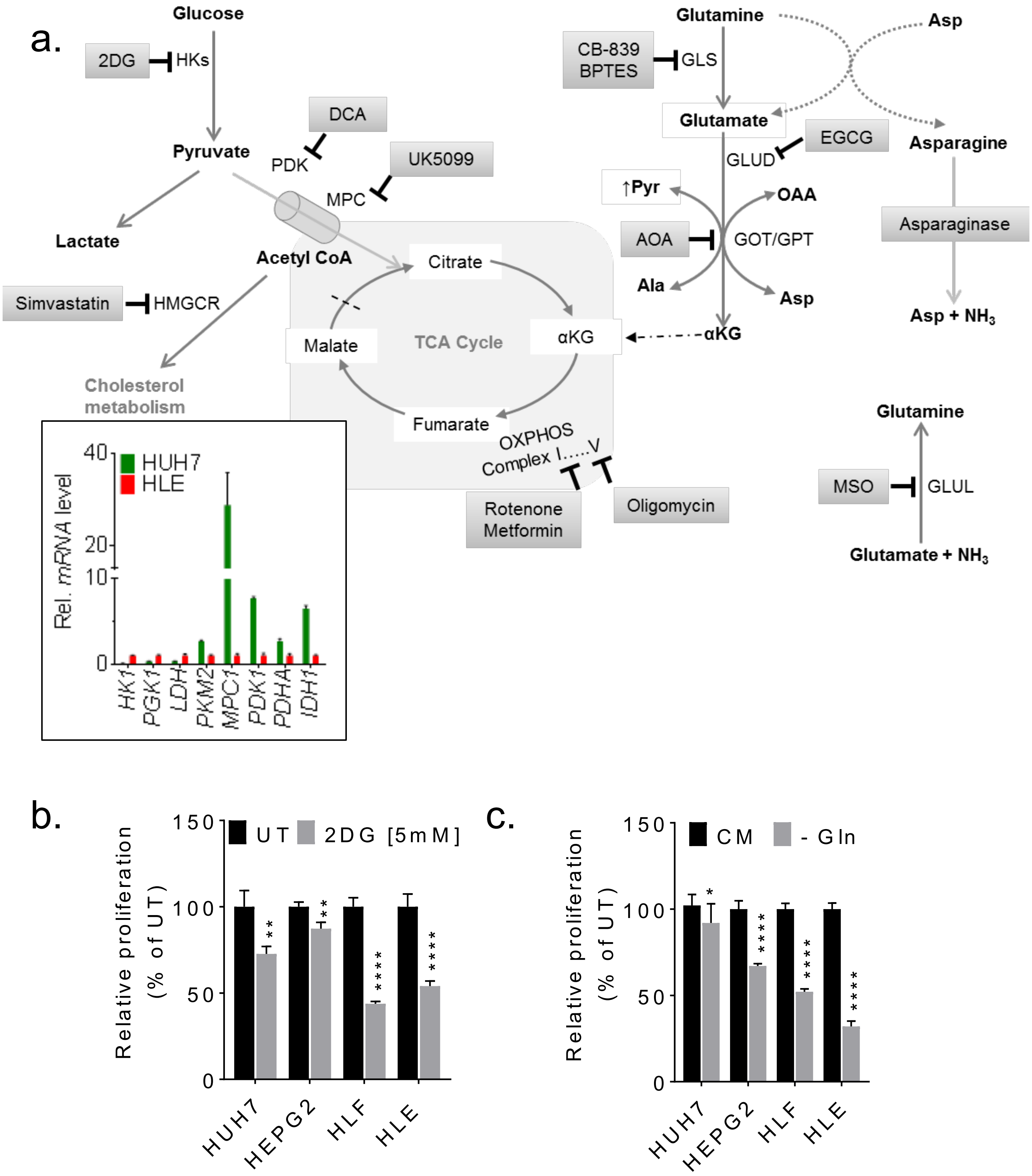

A. Schematic representation of metabolic pathways targeted in the HCC cell lines. Next to it is an additional qPCR data on the distinct expression of known metabolic targets related to glycolysis and the initial phase of TCA cycle.
B. MTT proliferation assay showing the effect of 2-deoxy-glucose (2DG) treatment on the HCC cell lines.
C. MTT proliferation assay showing the effect of extracellular glutamine withdrawal on the HCC cell lines. Bars represent mean ± SD; MTT assays were in quadruplicates, except HUH7 (n=8 per group). CM – complete medium; UT – Untreated.

References

1. Battello N, Zimmer AD, Goebel C, Dong X, Behrmann I, Haan C, et al (2016). The role of HIF-1 in oncostatin M-dependent metabolic reprogramming of hepatic cells. Cancer Metab. 4:3. doi: 10.1186/s40170-016-0141-0.
2. Chiu M, Tardito S, Pillozzi S, et al. (2014). Glutamine depletion by crisantaspase hinders the growth of human hepatocellular carcinoma xenografts. Br J Cancer. 111(6):1159-67. doi: 10.1038/bjc.2014.425.
3. Dropmann A, Dediulia T, Breitkopf-Heinlein K, et al. (2016). TGF-β1 and TGF-β2 abundance in liver diseases of mice and men. Oncotarget. 7:15. 19499-518. doi: 10.18632/oncotarget.6967.
4. Foretz M, Hébrard S, Leclerc J, Zarrinpashneh E, Soty M, Mithieux G, Sakamoto K, Andreelli F, Viollet B. Metformin inhibits hepatic gluconeogenesis in mice independently of the LKB1/AMPK pathway via a decrease in hepatic energy state. J Clin Invest.;120(7):2355-69. doi: 10.1172/JCI40671.
5. Li W, Wang J, Chen QD, Qian X, Li Q, Yin Y, Shi ZM, Wang L, Lin J, Liu LZ, Jiang BH (2013). Insulin promotes glucose consumption via regulation of miR-99a/mTOR/PKM2 pathway. PLoS One;8(6):e64924. doi: 10.1371/journal.pone.0064924.
6. Son J, Lyssiotis CA, Ying H, Wang X, Hua S, Ligorio M, Perera RM, Ferrone CR, Mullarky E, Shyh-Chang N, Kang Y, Fleming JB, Bardeesy N, Asara JM, Haigis MC, DePinho RA, Cantley LC, Kimmelman AC (2013). Glutamine supports pancreatic cancer growth through a KRAS-regulated metabolic pathway. Nature. 496(7443):101-5. doi: 10.1038/nature12040.

7. Zieker D, Königsrainer I, Tritschler I, Löffler M, Beckert S, Traub F, Nieselt K, Bühler S, Weller M, Gaedcke J, Taichman RS, Northoff H, Brücher BL, Königsrainer A (2010). Phosphoglycerate kinase 1 a promoting enzyme for peritoneal dissemination in gastric cancer. Int J Cancer.;126(6):1513-20. doi: 10.1002/ijc.24835.
