# Supplementary material for: Liver cancer cell lines distinctly mimic the metabolic gene expression pattern of the corresponding human tumours
Source: J Exp Clin Cancer Res. 2018 Sep 3;37:211. doi: 10.1186/s13046-018-0872-6 (PMC6122702; doi:10.1186/s13046-018-0872-6)
Supplement: Supplementary file 2 — Table S1. Genes more or lowly expressed in poorly differentiated relative to the well-differentiated cell lines. Includes all differentially expressed genes (i.e. in metabolism and other processes, P < 0.05). Datasets used: GSE57083, GSE36133 (CCLE) and GSE35818. (DOCX 166 kb) [file 13046_2018_872_MOESM2_ESM.docx]

**Table S1.** Genes more or lowly expressed in poorly differentiated relative to the well differentiated cell lines. Includes all differentially expressed genes (i.e. in metabolic and other processes, *P*<0.05). Datasets used: GSE57083, GSE36133 (CCLE) and GSE35818

| **(in bold)** 1,584 genes upregulated (or more expressed) in poorly differentiated HCC cell lines HLF, HLE and SNU-449 (combined) relative to well differentiated cell lines HUH7, HEPG2, HEP3B. | | | | 1,501 genes downregulated (or lowly expressed) in poorly differentiated HCC cell lines HLF, HLE and SNU-449 (combined) relative to well differentiated cell lines HUH7, HEPG2, HEP3B. | |
| --- | --- | --- | --- | --- | --- |
| ***SHISA2*** | ↑ in all three datasets |  |  | *APOA2* | ↓in all three datasets |
| ***BASP1*** | ↑ in all three datasets |  |  | *ALB* | ↓in all three datasets |
| ***RNF182*** | ↑ in all three datasets |  |  | *TF* | ↓in all three datasets |
| ***PLP2*** | ↑ in all three datasets |  |  | *AHSG* | ↓in all three datasets |
| ***PFKP*** | ↑ in all three datasets |  |  | *AFP* | ↓in all three datasets |
| ***MGARP*** | ↑ in all three datasets |  |  | *APOB* | ↓in all three datasets |
| ***TM4SF18*** | ↑ in all three datasets |  |  | *AMBP* | ↓in all three datasets |
| ***TENM2*** | ↑ in all three datasets |  |  | *RBP4* | ↓in all three datasets |
| ***LDHB*** | ↑ in all three datasets |  |  | *A2M* | ↓in all three datasets |
| ***TNC*** | ↑ in all three datasets |  |  | *GPC3* | ↓in all three datasets |
| ***PLCB4*** | ↑ in all three datasets |  |  | *APOH* | ↓in all three datasets |
| ***C15orf48*** | ↑ in all three datasets |  |  | *ITIH2* | ↓in all three datasets |
| ***CCL2*** | ↑ in all three datasets |  |  | *KRT19* | ↓in all three datasets |
| ***OCIAD2*** | ↑ in all three datasets |  |  | *ORM1* | ↓in all three datasets |
| ***GNG11*** | ↑ in all three datasets |  |  | *AGT* | ↓in all three datasets |
| ***EFHB*** | ↑ in all three datasets |  |  | *FABP1* | ↓in all three datasets |
| ***GPRC5A*** | ↑ in all three datasets |  |  | *SERPIND1* | ↓in all three datasets |
| ***ITGA3*** | ↑ in all three datasets |  |  | *SERPINA1* | ↓in all three datasets |
| ***CD44*** | ↑ in all three datasets |  |  | *APOA1* | ↓in all three datasets |
| ***TGFB1I1*** | ↑ in all three datasets |  |  | *PROM1* | ↓in all three datasets |
| ***ARL4C*** | ↑ in all three datasets |  |  | *EPCAM* | ↓in all three datasets |
| ***FZD2*** | ↑ in all three datasets |  |  | *SERPINF1* | ↓in all three datasets |
| ***TFPI2*** | ↑ in all three datasets |  |  | *VIL1* | ↓in all three datasets |
| ***CPA4*** | ↑ in all three datasets |  |  | *MAL2* | ↓in all three datasets |
| ***NNMT*** | ↑ in all three datasets |  |  | *CPB2* | ↓in all three datasets |
| ***WNT5B*** | ↑ in all three datasets |  |  | *AGMAT* | ↓in all three datasets |
| ***PLAU*** | ↑ in all three datasets |  |  | *CCL20* | ↓in all three datasets |
| ***CYP1B1*** | ↑ in all three datasets |  |  | *APOC3* | ↓in all three datasets |
| ***PROCR*** | ↑ in all three datasets |  |  | *MTTP* | ↓in all three datasets |
| ***AHNAK2*** | ↑ in all three datasets |  |  | *ASGR2* | ↓in all three datasets |
| ***FOSL1*** | ↑ in all three datasets |  |  | *KLB* | ↓in all three datasets |
| ***INPP4B*** | ↑ in all three datasets |  |  | *TTR* | ↓in all three datasets |
| ***FJX1*** | ↑ in all three datasets |  |  | *A1CF* | ↓in all three datasets |
| ***FRMD4A*** | ↑ in all three datasets |  |  | *GAS2* | ↓in all three datasets |
| ***FAM129A*** | ↑ in all three datasets |  |  | *F2* | ↓in all three datasets |
| ***BMP5*** | ↑ in all three datasets |  |  | *FGA* | ↓in all three datasets |
| ***TMEM47*** | ↑ in all three datasets |  |  | *LINC00261* | ↓in all three datasets |
| ***MYOF*** | ↑ in all three datasets |  |  | *GPX3* | ↓in all three datasets |
| ***FXYD5*** | ↑ in all three datasets |  |  | *C3* | ↓in all three datasets |
| ***CDC42EP3*** | ↑ in all three datasets |  |  | *TOX3* | ↓in all three datasets |
| ***TSPAN5*** | ↑ in all three datasets |  |  | *SERPINA5* | ↓in all three datasets |
| ***SEMA3C*** | ↑ in all three datasets |  |  | *IQGAP2* | ↓in all three datasets |
| ***AXL*** | ↑ in all three datasets |  |  | *HGD* | ↓in all three datasets |
| ***PTPN13*** | ↑ in all three datasets |  |  | *INHBE* | ↓in all three datasets |
| ***EMP3*** | ↑ in all three datasets |  |  | *C5* | ↓in all three datasets |
| ***MYL9*** | ↑ in all three datasets |  |  | *CEBPA* | ↓in all three datasets |
| ***FGF2*** | ↑ in all three datasets |  |  | *CFI* | ↓in all three datasets |
| ***MACROD2*** | ↑ in all three datasets |  |  | *ASGR1* | ↓in all three datasets |
| ***LYSMD2*** | ↑ in all three datasets |  |  | *APOE* | ↓in all three datasets |
| ***IGFBP7*** | ↑ in all three datasets |  |  | *VSNL1* | ↓in all three datasets |
| ***RASSF2*** | ↑ in all three datasets |  |  | *PRDX2* | ↓in all three datasets |
| ***WNT5A*** | ↑ in all three datasets |  |  | *KANK1* | ↓in all three datasets |
| ***GBP3*** | ↑ in all three datasets |  |  | *FGL1* | ↓in all three datasets |
| ***PTX3*** | ↑ in all three datasets |  |  | *MBNL3* | ↓in all three datasets |
| ***CAV1*** | ↑ in all three datasets |  |  | *DUSP9* | ↓in all three datasets |
| ***IFI16*** | ↑ in all three datasets |  |  | *APOC1* | ↓in all three datasets |
| ***DOCK10*** | ↑ in all three datasets |  |  | *PROX1* | ↓in all three datasets |
| ***MSN*** | ↑ in all three datasets |  |  | *FOXA1* | ↓in all three datasets |
| ***SERTAD4-AS1*** | ↑ in all three datasets |  |  | *TM4SF5* | ↓in all three datasets |
| ***PRSS23*** | ↑ in all three datasets |  |  | *UGT2B4* | ↓in all three datasets |
| ***TNNT1*** | ↑ in all three datasets |  |  | *FOXQ1* | ↓in all three datasets |
| ***CYBRD1*** | ↑ in all three datasets |  |  | *DSP* | ↓in all three datasets |
| ***COL4A1*** | ↑ in all three datasets |  |  | *FST* | ↓in all three datasets |
| ***SLC35F2*** | ↑ in all three datasets |  |  | *DMKN* | ↓in all three datasets |
| ***SFTA1P*** | ↑ in all three datasets |  |  | *SLC30A10* | ↓in all three datasets |
| ***SRPX*** | ↑ in all three datasets |  |  | *ACSS3* | ↓in all three datasets |
| ***TWIST1*** | ↑ in all three datasets |  |  | *DPP4* | ↓in all three datasets |
| ***ZNF462*** | ↑ in all three datasets |  |  | *SERPINA6* | ↓in all three datasets |
| ***S100A2*** | ↑ in all three datasets |  |  | *ENPP2* | ↓in all three datasets |
| ***LINC01420*** | ↑ in all three datasets |  |  | *ABAT* | ↓in all three datasets |
| ***FMN2*** | ↑ in all three datasets |  |  | *ANPEP* | ↓in all three datasets |
| ***ANXA1*** | ↑ in all three datasets |  |  | *SLC43A1* | ↓in all three datasets |
| ***PMEPA1*** | ↑ in all three datasets |  |  | *MAN1A1* | ↓in all three datasets |
| ***TMEM158*** | ↑ in all three datasets |  |  | *CPVL* | ↓in all three datasets |
| ***RFTN1*** | ↑ in all three datasets |  |  | *KRT23* | ↓in all three datasets |
| ***CNRIP1*** | ↑ in all three datasets |  |  | *AKR1D1* | ↓in all three datasets |
| ***SPARC*** | ↑ in all three datasets |  |  | *CHST13* | ↓in all three datasets |
| ***CTBP2*** | ↑ in all three datasets |  |  | *KANK4* | ↓in all three datasets |
| ***PRKCDBP*** | ↑ in all three datasets |  |  | *APOM* | ↓in all three datasets |
| ***SERTAD4*** | ↑ in all three datasets |  |  | *PROC* | ↓in all three datasets |
| ***ETS1*** | ↑ in all three datasets |  |  | *RHOU* | ↓in all three datasets |
| ***C11orf70*** | ↑ in all three datasets |  |  | *LIPC* | ↓in all three datasets |
| ***S100A6*** | ↑ in all three datasets |  |  | *MGAT4A* | ↓in all three datasets |
| ***SLC16A2*** | ↑ in all three datasets |  |  | *F5* | ↓in all three datasets |
| ***FGF5*** | ↑ in all three datasets |  |  | *NR1H4* | ↓in all three datasets |
| ***GSPT2*** | ↑ in all three datasets |  |  | *ALDH2* | ↓in all three datasets |
| ***GEM*** | ↑ in all three datasets |  |  | *GOLT1A* | ↓in all three datasets |
| ***ATP8B1*** | ↑ in all three datasets |  |  | *FOXA3* | ↓in all three datasets |
| ***INHBA*** | ↑ in all three datasets |  |  | *FOXA2* | ↓in all three datasets |
| ***SH3BGRL*** | ↑ in all three datasets |  |  | *IL17RB* | ↓in all three datasets |
| ***SQRDL*** | ↑ in all three datasets |  |  | *FGG* | ↓in all three datasets |
| ***KLHL4*** | ↑ in all three datasets |  |  | *SULT2A1* | ↓in all three datasets |
| ***SUN3*** | ↑ in all three datasets |  |  | *UGT2A3* | ↓in all three datasets |
| ***RGS20*** | ↑ in all three datasets |  |  | *ST3GAL6* | ↓in all three datasets |
| ***STAMBPL1*** | ↑ in all three datasets |  |  | *CRLF1* | ↓in all three datasets |
| ***PRR16*** | ↑ in all three datasets |  |  | *TSPAN13* | ↓in all three datasets |
| ***AFAP1L2*** | ↑ in all three datasets |  |  | *NR0B2* | ↓in all three datasets |
| ***CAV2*** | ↑ in all three datasets |  |  | *HAL* | ↓in all three datasets |
| ***ADORA2B*** | ↑ in all three datasets |  |  | *FGFR3* | ↓in all three datasets |
| ***C14orf105*** | ↑ in all three datasets |  |  | *METTL7A* | ↓in all three datasets |
| ***SERPINE1*** | ↑ in all three datasets |  |  | *SELENBP1* | ↓in all three datasets |
| ***BDNF*** | ↑ in all three datasets |  |  | *SULT1A1* | ↓in all three datasets |
| ***TSPO*** | ↑ in all three datasets |  |  | *DNAJC22* | ↓in all three datasets |
| ***KRT80*** | ↑ in all three datasets |  |  | *LSR* | ↓in all three datasets |
| ***DDX60*** | ↑ in all three datasets |  |  | *ASS1* | ↓in all three datasets |
| ***APOBEC3G*** | ↑ in all three datasets |  |  | *ST6GAL1* | ↓in all three datasets |
| ***MVP*** | ↑ in all three datasets |  |  | *IGDCC3* | ↓in all three datasets |
| ***WLS*** | ↑ in all three datasets |  |  | *HSD17B2* | ↓in all three datasets |
| ***GALNT14*** | ↑ in all three datasets |  |  | *SNTB1* | ↓in all three datasets |
| ***MLLT11*** | ↑ in all three datasets |  |  | *RBP1* | ↓in all three datasets |
| ***CAPN2*** | ↑ in all three datasets |  |  | *SERPINF2* | ↓in all three datasets |
| ***NMI*** | ↑ in all three datasets |  |  | *TCEA3* | ↓in all three datasets |
| ***HK1*** | ↑ in all three datasets |  |  | *CIDEB* | ↓in all three datasets |
| ***ME3*** | ↑ in all three datasets |  |  | *ADH6* | ↓in all three datasets |
| ***GPX8*** | ↑ in all three datasets |  |  | *C2* | ↓in all three datasets |
| ***LGALS1*** | ↑ in all three datasets |  |  | *PLA2G12B* | ↓in all three datasets |
| ***NMU*** | ↑ in all three datasets |  |  | *RGN* | ↓in all three datasets |
| ***NLGN4X*** | ↑ in all three datasets |  |  | *DDC* | ↓in all three datasets |
| ***GNG12*** | ↑ in all three datasets |  |  | *PDE9A* | ↓in all three datasets |
| ***ATP10A*** | ↑ in all three datasets |  |  | *MDK* | ↓in all three datasets |
| ***LOXL2*** | ↑ in all three datasets |  |  | *PHYHIPL* | ↓in all three datasets |
| ***LINC00839*** | ↑ in all three datasets |  |  | *ARG1* | ↓in all three datasets |
| ***KCNMA1*** | ↑ in all three datasets |  |  | *FZD5* | ↓in all three datasets |
| ***FSTL1*** | ↑ in all three datasets |  |  | *MPZL2* | ↓in all three datasets |
| ***UGCG*** | ↑ in all three datasets |  |  | *ERBB3* | ↓in all three datasets |
| ***LHX2*** | ↑ in all three datasets |  |  | *LRP5* | ↓in all three datasets |
| ***RRAS*** | ↑ in all three datasets |  |  | *HPN* | ↓in all three datasets |
| ***PDP1*** | ↑ in all three datasets |  |  | *TSPAN8* | ↓in all three datasets |
| ***PAX8-AS1*** | ↑ in all three datasets |  |  | *SALL1* | ↓in all three datasets |
| ***NFIB*** | ↑ in all three datasets |  |  | *GRTP1* | ↓in all three datasets |
| ***AMIGO2*** | ↑ in all three datasets |  |  | *RAPGEF5* | ↓in all three datasets |
| ***CBR3*** | ↑ in all three datasets |  |  | *GPR160* | ↓in all three datasets |
| ***KIRREL*** | ↑ in all three datasets |  |  | *SCRN1* | ↓in all three datasets |
| ***NEXN*** | ↑ in all three datasets |  |  | *SLC22A9* | ↓in all three datasets |
| ***FHL2*** | ↑ in all three datasets |  |  | *TSPAN7* | ↓in all three datasets |
| ***APBB1IP*** | ↑ in all three datasets |  |  | *GLDC* | ↓in all three datasets |
| ***SLFN12*** | ↑ in all three datasets |  |  | *SH3BGRL2* | ↓in all three datasets |
| ***GPR137B*** | ↑ in all three datasets |  |  | *SLC16A10* | ↓in all three datasets |
| ***SLITRK5*** | ↑ in all three datasets |  |  | *CKB* | ↓in all three datasets |
| ***PRKAG2*** | ↑ in all three datasets |  |  | *AMDHD1* | ↓in all three datasets |
| ***PMAIP1*** | ↑ in all three datasets |  |  | *NPW* | ↓in all three datasets |
| ***NTN4*** | ↑ in all three datasets |  |  | *AMT* | ↓in all three datasets |
| ***PCDHB6*** | ↑ in all three datasets |  |  | *ZHX2* | ↓in all three datasets |
| ***CHST7*** | ↑ in all three datasets |  |  | *EPAS1* | ↓in all three datasets |
| ***WDR54*** | ↑ in all three datasets |  |  | *VWCE* | ↓in all three datasets |
| ***ANTXR1*** | ↑ in all three datasets |  |  | *METTL7B* | ↓in all three datasets |
| ***SLIT3*** | ↑ in all three datasets |  |  | *F10* | ↓in all three datasets |
| ***C8orf48*** | ↑ in all three datasets |  |  | *ARID3A* | ↓in all three datasets |
| ***ZNF239*** | ↑ in all three datasets |  |  | *MBL2* | ↓in all three datasets |
| ***PSMB9*** | ↑ in all three datasets |  |  | *FOXO1* | ↓in all three datasets |
| ***TIMP1*** | ↑ in all three datasets |  |  | *SLC27A2* | ↓in all three datasets |
| ***HAS3*** | ↑ in all three datasets |  |  | *MGST2* | ↓in all three datasets |
| ***UPP1*** | ↑ in all three datasets |  |  | *ACOX2* | ↓in all three datasets |
| ***GBP1*** | ↑ in all three datasets |  |  | *GCHFR* | ↓in all three datasets |
| ***NAV1*** | ↑ in all three datasets |  |  | *MTUS1* | ↓in all three datasets |
| ***MID1*** | ↑ in all three datasets |  |  | *PPFIBP2* | ↓in all three datasets |
| ***TFAP2A*** | ↑ in all three datasets |  |  | *SLC38A11* | ↓in all three datasets |
| ***GLIPR1*** | ↑ in all three datasets |  |  | *C4BPA* | ↓in all three datasets |
| ***DDX60L*** | ↑ in all three datasets |  |  | *CLIC3* | ↓in all three datasets |
| ***MITF*** | ↑ in all three datasets |  |  | *CHN2* | ↓in all three datasets |
| ***MAGEH1*** | ↑ in all three datasets |  |  | *COLCA2* | ↓in all three datasets |
| ***CD109*** | ↑ in all three datasets |  |  | *LRG1* | ↓in all three datasets |
| ***COTL1*** | ↑ in all three datasets |  |  | *SLC17A9* | ↓in all three datasets |
| ***SCD5*** | ↑ in all three datasets |  |  | *CFB* | ↓in all three datasets |
| ***ANXA2R*** | ↑ in all three datasets |  |  | *TMEM45B* | ↓in all three datasets |
| ***FGFR1*** | ↑ in all three datasets |  |  | *GIPC2* | ↓in all three datasets |
| ***PLAT*** | ↑ in all three datasets |  |  | *ANXA9* | ↓in all three datasets |
| ***EPHB2*** | ↑ in all three datasets |  |  | *COBL* | ↓in all three datasets |
| ***TMEM156*** | ↑ in all three datasets |  |  | *GCH1* | ↓in all three datasets |
| ***KCNN4*** | ↑ in all three datasets |  |  | *FN1* | ↓in all three datasets |
| ***HCFC1R1*** | ↑ in all three datasets |  |  | *RASD1* | ↓in all three datasets |
| ***EFNA5*** | ↑ in all three datasets |  |  | *PC* | ↓in all three datasets |
| ***EDIL3*** | ↑ in all three datasets |  |  | *LEAP2* | ↓in all three datasets |
| ***PPME1*** | ↑ in all three datasets |  |  | *CYP1A1* | ↓in all three datasets |
| ***UBASH3B*** | ↑ in all three datasets |  |  | *CPN1* | ↓in all three datasets |
| ***PRICKLE2*** | ↑ in all three datasets |  |  | *CORO2A* | ↓in all three datasets |
| ***ADRB2*** | ↑ in all three datasets |  |  | *INHBB* | ↓in all three datasets |
| ***PAQR5*** | ↑ in all three datasets |  |  | *MARC1* | ↓in all three datasets |
| ***MOK*** | ↑ in all three datasets |  |  | *MST1* | ↓in all three datasets |
| ***AKT3*** | ↑ in all three datasets |  |  | *SLC2A2* | ↓in all three datasets |
| ***S100A11*** | ↑ in all three datasets |  |  | *HPR* | ↓in all three datasets |
| ***TWSG1*** | ↑ in all three datasets |  |  | *REEP6* | ↓in all three datasets |
| ***TMEM40*** | ↑ in all three datasets |  |  | *SERPINA10* | ↓in all three datasets |
| ***RGS10*** | ↑ in all three datasets |  |  | *GPRC5C* | ↓in all three datasets |
| ***ERAP2*** | ↑ in all three datasets |  |  | *FAM19A4* | ↓in all three datasets |
| ***TRAF5*** | ↑ in all three datasets |  |  | *RNF43* | ↓in all three datasets |
| ***RBM20*** | ↑ in all three datasets |  |  | *SCARB1* | ↓in all three datasets |
| ***EHD2*** | ↑ in all three datasets |  |  | *HNMT* | ↓in all three datasets |
| ***ADAMTS3*** | ↑ in all three datasets |  |  | *SLC7A2* | ↓in all three datasets |
| ***NMT2*** | ↑ in all three datasets |  |  | *CHKA* | ↓in all three datasets |
| ***MLKL*** | ↑ in all three datasets |  |  | *FGFR4* | ↓in all three datasets |
| ***TSHZ3*** | ↑ in all three datasets |  |  | *SLC5A9* | ↓in all three datasets |
| ***UBE2E2*** | ↑ in all three datasets |  |  | *DUSP6* | ↓in all three datasets |
| ***PRAF2*** | ↑ in all three datasets |  |  | *COCH* | ↓in all three datasets |
| ***TMEM54*** | ↑ in all three datasets |  |  | *SMPDL3A* | ↓in all three datasets |
| ***SVIL*** | ↑ in all three datasets |  |  | *COBLL1* | ↓in all three datasets |
| ***HYLS1*** | ↑ in all three datasets |  |  | *F7* | ↓in all three datasets |
| ***PLAUR*** | ↑ in all three datasets |  |  | *IL1RAP* | ↓in all three datasets |
| ***MARC4*** | ↑ in all three datasets |  |  | *TRIM24* | ↓in all three datasets |
| ***MTCL1*** | ↑ in all three datasets |  |  | *NMB* | ↓in all three datasets |
| ***SACS*** | ↑ in all three datasets |  |  | *FRAT2* | ↓in all three datasets |
| ***STAC*** | ↑ in all three datasets |  |  | *HPX* | ↓in all three datasets |
| ***MAMLD1*** | ↑ in all three datasets |  |  | *SLC51B* | ↓in all three datasets |
| ***ADA*** | ↑ in all three datasets |  |  | *TST* | ↓in all three datasets |
| ***CABYR*** | ↑ in all three datasets |  |  | *CXCL16* | ↓in all three datasets |
| ***MNS1*** | ↑ in all three datasets |  |  | *SETD6* | ↓in all three datasets |
| ***PTRF*** | ↑ in all three datasets |  |  | *SLC47A1* | ↓in all three datasets |
| ***IFIT3*** | ↑ in all three datasets |  |  | *C4BPB* | ↓in all three datasets |
| ***OGFRL1*** | ↑ in all three datasets |  |  | *ONECUT2* | ↓in all three datasets |
| ***BCAR3*** | ↑ in all three datasets |  |  | *HMGCS1* | ↓in all three datasets |
| ***RBMS3*** | ↑ in all three datasets |  |  | *DENND4A* | ↓in all three datasets |
| ***PQLC3*** | ↑ in all three datasets |  |  | *TMEM117* | ↓in all three datasets |
| ***C1orf216*** | ↑ in all three datasets |  |  | *RAB17* | ↓in all three datasets |
| ***B9D1*** | ↑ in all three datasets |  |  | *PNPLA3* | ↓in all three datasets |
| ***ELFN2*** | ↑ in all three datasets |  |  | *HSD11B2* | ↓in all three datasets |
| ***VLDLR*** | ↑ in all three datasets |  |  | *FBLN1* | ↓in all three datasets |
| ***OXCT1*** | ↑ in all three datasets |  |  | *GPX7* | ↓in all three datasets |
| ***ARSJ*** | ↑ in all three datasets |  |  | *SOAT2* | ↓in all three datasets |
| ***FRMD6*** | ↑ in all three datasets |  |  | *SLC9A3R1* | ↓in all three datasets |
| ***ANKRD29*** | ↑ in all three datasets |  |  | *CPPED1* | ↓in all three datasets |
| ***PPP1R18*** | ↑ in all three datasets |  |  | *VAMP5* | ↓in all three datasets |
| ***DCBLD2*** | ↑ in all three datasets |  |  | *F12* | ↓in all three datasets |
| ***IGFBP3*** | ↑ in all three datasets |  |  | *GPER1* | ↓in all three datasets |
| ***BIRC3*** | ↑ in all three datasets |  |  | *HHEX* | ↓in all three datasets |
| ***F8*** | ↑ in all three datasets |  |  | *CD7* | ↓in all three datasets |
| ***UPRT*** | ↑ in all three datasets |  |  | *TMED6* | ↓in all three datasets |
| ***LEPROT*** | ↑ in all three datasets |  |  | *C2orf72* | ↓in all three datasets |
| ***SFR1*** | ↑ in all three datasets |  |  | *SHROOM2* | ↓in all three datasets |
| ***OSCP1*** | ↑ in all three datasets |  |  | *STARD10* | ↓in all three datasets |
| ***HOXB7*** | ↑ in all three datasets |  |  | *ENPP1* | ↓in all three datasets |
| ***BATF3*** | ↑ in all three datasets |  |  | *TM7SF2* | ↓in all three datasets |
| ***CYR61*** | ↑ in all three datasets |  |  | *PLCB1* | ↓in all three datasets |
| ***HRH1*** | ↑ in all three datasets |  |  | *LRRC1* | ↓in all three datasets |
| ***PLK2*** | ↑ in all three datasets |  |  | *TTC38* | ↓in all three datasets |
| ***SMURF2*** | ↑ in all three datasets |  |  | *FRY* | ↓in all three datasets |
| ***TMEM136*** | ↑ in all three datasets |  |  | *ZBED6CL* | ↓in all three datasets |
| ***PNMA2*** | ↑ in all three datasets |  |  | *ELL2* | ↓in all three datasets |
| ***TANC2*** | ↑ in all three datasets |  |  | *ZNF280B* | ↓in all three datasets |
| ***CD70*** | ↑ in all three datasets |  |  | *EPB41L5* | ↓in all three datasets |
| ***LAT2*** | ↑ in all three datasets |  |  | *ENO3* | ↓in all three datasets |
| ***GFPT2*** | ↑ in all three datasets |  |  | *ESPN* | ↓in all three datasets |
| ***ELOVL7*** | ↑ in all three datasets |  |  | *ALDOC* | ↓in all three datasets |
| ***ENDOD1*** | ↑ in all three datasets |  |  | *MAP4K4* | ↓in all three datasets |
| ***ADM*** | ↑ in all three datasets |  |  | *ONECUT1* | ↓in all three datasets |
| ***ARHGAP29*** | ↑ in all three datasets |  |  | *TNFAIP3* | ↓in all three datasets |
| ***GPR39*** | ↑ in all three datasets |  |  | *MPC1* | ↓in all three datasets |
| ***ITGBL1*** | ↑ in all three datasets |  |  | *CBLB* | ↓in all three datasets |
| ***HOXB6*** | ↑ in all three datasets |  |  | *GLYCTK* | ↓in all three datasets |
| ***TTLL7*** | ↑ in all three datasets |  |  | *GSE1* | ↓in all three datasets |
| ***CCDC113*** | ↑ in all three datasets |  |  | *SEMA4G* | ↓in all three datasets |
| ***SYNE1*** | ↑ in all three datasets |  |  | *PLXNC1* | ↓in all three datasets |
| ***CPEB2*** | ↑ in all three datasets |  |  | *NID1* | ↓in all three datasets |
| ***C12orf75*** | ↑ in all three datasets |  |  | *YPEL3* | ↓in all three datasets |
| ***DRAP1*** | ↑ in all three datasets |  |  | *GK* | ↓in all three datasets |
| ***HERC4*** | ↑ in all three datasets |  |  | *DOCK8* | ↓in all three datasets |
| ***TPM1*** | ↑ in all three datasets |  |  | *ZNF124* | ↓in all three datasets |
| ***KAT2B*** | ↑ in all three datasets |  |  | *PTP4A3* | ↓in all three datasets |
| ***FAM127B*** | ↑ in all three datasets |  |  | *RND1* | ↓in all three datasets |
| ***CAMK1D*** | ↑ in all three datasets |  |  | *DHCR24* | ↓in all three datasets |
| ***CD40*** | ↑ in all three datasets |  |  | *XBP1* | ↓in all three datasets |
| ***ABCC4*** | ↑ in all three datasets |  |  | *GJB1* | ↓in all three datasets |
| ***CD200*** | ↑ in all three datasets |  |  | *CMTM8* | ↓in all three datasets |
| ***ARHGAP24*** | ↑ in all three datasets |  |  | *MARVELD2* | ↓in all three datasets |
| ***MAP7D1*** | ↑ in all three datasets |  |  | *IGDCC4* | ↓in all three datasets |
| ***C1QL1*** | ↑ in all three datasets |  |  | *CLGN* | ↓in all three datasets |
| ***MYBL1*** | ↑ in all three datasets |  |  | *MAOA* | ↓in all three datasets |
| ***CTTNBP2NL*** | ↑ in all three datasets |  |  | *SESN1* | ↓in all three datasets |
| ***MSRB3*** | ↑ in all three datasets |  |  | *SEMA6A* | ↓in all three datasets |
| ***IFIT5*** | ↑ in all three datasets |  |  | *TJP2* | ↓in all three datasets |
| ***WDR47*** | ↑ in all three datasets |  |  | *CREB3L2* | ↓in all three datasets |
| ***TRIM68*** | ↑ in all three datasets |  |  | *OAF* | ↓in all three datasets |
| ***HDX*** | ↑ in all three datasets |  |  | *ZRANB3* | ↓in all three datasets |
| ***WISP2*** | ↑ in all three datasets |  |  | *PER2* | ↓in all three datasets |
| ***PTPN14*** | ↑ in all three datasets |  |  | *ABCB4* | ↓in all three datasets |
| ***CRTAP*** | ↑ in all three datasets |  |  | *CDH1* | ↓in all three datasets |
| ***COL6A2*** | ↑ in all three datasets |  |  | *PIEZO2* | ↓in all three datasets |
| ***MB21D2*** | ↑ in all three datasets |  |  | *TNFSF4* | ↓in all three datasets |
| ***ANKRD33B*** | ↑ in all three datasets |  |  | *NGEF* | ↓in all three datasets |
| ***MB21D1*** | ↑ in all three datasets |  |  | *NR1H3* | ↓in all three datasets |
| ***RSU1*** | ↑ in all three datasets |  |  | *DOK4* | ↓in all three datasets |
| ***NAP1L2*** | ↑ in all three datasets |  |  | *SLC17A2* | ↓in all three datasets |
| ***ARHGEF9*** | ↑ in all three datasets |  |  | *SLC38A3* | ↓in all three datasets |
| ***KIF3A*** | ↑ in all three datasets |  |  | *PROS1* | ↓in all three datasets |
| ***ZNF25*** | ↑ in all three datasets |  |  | *TET1* | ↓in all three datasets |
| ***ROBO3*** | ↑ in all three datasets |  |  | *STXBP6* | ↓in all three datasets |
| ***SLC26A2*** | ↑ in all three datasets |  |  | *ELOVL2* | ↓in all three datasets |
| ***AP1S2*** | ↑ in all three datasets |  |  | *F11R* | ↓in all three datasets |
| ***CEP55*** | ↑ in all three datasets |  |  | *ITPKA* | ↓in all three datasets |
| ***IDS*** | ↑ in all three datasets |  |  | *CAMSAP3* | ↓in all three datasets |
| ***BNC2*** | ↑ in all three datasets |  |  | *CHDH* | ↓in all three datasets |
| ***ZNF766*** | ↑ in all three datasets |  |  | *BCR* | ↓in all three datasets |
| ***NEIL2*** | ↑ in all three datasets |  |  | *SLC35D2* | ↓in all three datasets |
| ***LOC100288911*** | ↑ in all three datasets |  |  | *CERS4* | ↓in all three datasets |
| ***MAP3K14*** | ↑ in all three datasets |  |  | *PIK3AP1* | ↓in all three datasets |
| ***TRIM9*** | ↑ in all three datasets |  |  | *SDC1* | ↓in all three datasets |
| ***ANGPTL4*** | ↑ in all three datasets |  |  | *CCR6* | ↓in all three datasets |
| ***AHRR*** | ↑ in all three datasets |  |  | *EGLN3* | ↓in all three datasets |
| ***CCDC80*** | ↑ in all three datasets |  |  | *GLUL* | ↓in all three datasets |
| ***NRG1*** | ↑ in all three datasets |  |  | *TMCC1* | ↓in all three datasets |
| ***LINC00847*** | ↑ in all three datasets |  |  | *PHACTR2* | ↓in all three datasets |
| ***CMC4*** | ↑ in all three datasets |  |  | *C1orf226* | ↓in all three datasets |
| ***JRKL*** | ↑ in all three datasets |  |  | *LAD1* | ↓in all three datasets |
| ***DNASE1L1*** | ↑ in all three datasets |  |  | *SLC18B1* | ↓in all three datasets |
| ***ABCA3*** | ↑ in all three datasets |  |  | *SLC39A5* | ↓in all three datasets |
| ***CHIC1*** | ↑ in all three datasets |  |  | *KNG1* | ↓in all three datasets |
| ***ADCY3*** | ↑ in all three datasets |  |  | *NHSL1* | ↓in all three datasets |
| ***LINC00094*** | ↑ in all three datasets |  |  | *IQSEC1* | ↓in all three datasets |
| ***SGMS2*** | ↑ in all three datasets |  |  | *SERPINB9* | ↓in all three datasets |
| ***RPGRIP1L*** | ↑ in all three datasets |  |  | *ATP7B* | ↓in all three datasets |
| ***CACNB3*** | ↑ in all three datasets |  |  | *INSR* | ↓in all three datasets |
| ***CBX6*** | ↑ in all three datasets |  |  | *PTP4A1* | ↓in all three datasets |
| ***PADI3*** | ↑ in all three datasets |  |  | *ATP2B1* | ↓in all three datasets |
| ***GGACT*** | ↑ in all three datasets |  |  | *IKBKAP* | ↓in all three datasets |
| ***LYST*** | ↑ in all three datasets |  |  | *ZFAND2A* | ↓in all three datasets |
| ***AFAP1L1*** | ↑ in all three datasets |  |  | *RBP5* | ↓in all three datasets |
| ***ZNFX1*** | ↑ in all three datasets |  |  | *SLC35D1* | ↓in all three datasets |
| ***YBEY*** | ↑ in all three datasets |  |  | *RPS27L* | ↓in all three datasets |
| ***FLNA*** | ↑ in all three datasets |  |  | *POU2AF1* | ↓in all three datasets |
| ***KCNB1*** | ↑ in all three datasets |  |  | *GATA2* | ↓in all three datasets |
| ***RNF217*** | ↑ in all three datasets |  |  | *RAP1GAP* | ↓in all three datasets |
| ***METRN*** | ↑ in all three datasets |  |  | *SLC38A4* | ↓in all three datasets |
| ***SLC41A1*** | ↑ in all three datasets |  |  | *CX3CL1* | ↓in all three datasets |
| ***CCDC181*** | ↑ in all three datasets |  |  | *DOK6* | ↓in all three datasets |
| ***FAM104B*** | ↑ in all three datasets |  |  | *CPD* | ↓in all three datasets |
| ***TMEM159*** | ↑ in all three datasets |  |  | *FAM117A* | ↓in all three datasets |
| ***TCP11L1*** | ↑ in all three datasets |  |  | *SLC29A4* | ↓in all three datasets |
| ***NAV3*** | ↑ in all three datasets |  |  | *FES* | ↓in all three datasets |
| ***MSL3*** | ↑ in all three datasets |  |  | *HIC2* | ↓in all three datasets |
| ***VCL*** | ↑ in all three datasets |  |  | *ENTPD5* | ↓in all three datasets |
| ***SYT1*** | ↑ in all three datasets |  |  | *HECTD1* | ↓in all three datasets |
| ***TLR6*** | ↑ in all three datasets |  |  | *PPARGC1A* | ↓in all three datasets |
| ***SAMD9*** | ↑ in all three datasets |  |  | *HNF1A* | ↓in all three datasets |
| ***PKD2*** | ↑ in all three datasets |  |  | *SUSD1* | ↓in all three datasets |
| ***FBXL2*** | ↑ in all three datasets |  |  | *CYP2J2* | ↓in all three datasets |
| ***MXRA7*** | ↑ in all three datasets |  |  | *GRHL1* | ↓in all three datasets |
| ***MPP1*** | ↑ in all three datasets |  |  | *GLUD1* | ↓in all three datasets |
| ***CLIP4*** | ↑ in all three datasets |  |  | *RAB11FIP4* | ↓in all three datasets |
| ***PPP2R3A*** | ↑ in all three datasets |  |  | *PLXNB1* | ↓in all three datasets |
| ***HRAS*** | ↑ in all three datasets |  |  | *GSTM4* | ↓in all three datasets |
| ***SPA17*** | ↑ in all three datasets |  |  | *HEATR5A* | ↓in all three datasets |
| ***ZNF32*** | ↑ in all three datasets |  |  | *TYRO3* | ↓in all three datasets |
| ***DYNC2H1*** | ↑ in all three datasets |  |  | *SOBP* | ↓in all three datasets |
| ***GLI2*** | ↑ in all three datasets |  |  | *ORMDL3* | ↓in all three datasets |
| ***DYX1C1*** | ↑ in all three datasets |  |  | *SLC19A2* | ↓in all three datasets |
| ***MYO5A*** | ↑ in all three datasets |  |  | *PRR5* | ↓in all three datasets |
| ***C15orf65*** | ↑ in all three datasets |  |  | *ZKSCAN1* | ↓in all three datasets |
| ***FHOD1*** | ↑ in all three datasets |  |  | *ROBO1* | ↓in all three datasets |
| ***LNPEP*** | ↑ in all three datasets |  |  | *MAST4* | ↓in all three datasets |
| ***HECTD2*** | ↑ in all three datasets |  |  | *TMEM181* | ↓in all three datasets |
| ***SPG20*** | ↑ in all three datasets |  |  | *AQP3* | ↓in all three datasets |
| ***TRIM59*** | ↑ in all three datasets |  |  | *NPC1L1* | ↓in all three datasets |
| ***TMEM231*** | ↑ in all three datasets |  |  | *ACVR2A* | ↓in all three datasets |
| ***MSANTD3*** | ↑ in all three datasets |  |  | *CTAGE5* | ↓in all three datasets |
| ***SLC46A3*** | ↑ in all three datasets |  |  | *LNX2* | ↓in all three datasets |
| ***NMRAL1*** | ↑ in all three datasets |  |  | *PPP1R1C* | ↓in all three datasets |
| ***CUEDC2*** | ↑ in all three datasets |  |  | *PDE3B* | ↓in all three datasets |
| ***ERCC6L*** | ↑ in all three datasets |  |  | *GLTPD2* | ↓in all three datasets |
| ***CKLF*** | ↑ in all three datasets |  |  | *TMEM150A* | ↓in all three datasets |
| ***TCEAL1*** | ↑ in all three datasets |  |  | *SERPINB1* | ↓in all three datasets |
| ***TLR3*** | ↑ in all three datasets |  |  | *TIGD2* | ↓in all three datasets |
| ***DNMBP*** | ↑ in all three datasets |  |  | *ITPR2* | ↓in all three datasets |
| ***LINC00472*** | ↑ in all three datasets |  |  | *CRB3* | ↓in all three datasets |
| ***LURAP1L*** | ↑ in all three datasets |  |  | *DUSP16* | ↓in all three datasets |
| ***CAMK4*** | ↑ in all three datasets |  |  | *FURIN* | ↓in all three datasets |
| ***SH3RF1*** | ↑ in all three datasets |  |  | *TRIM15* | ↓in all three datasets |
| ***BCAS4*** | ↑ in all three datasets |  |  | *NUP210* | ↓in all three datasets |
| ***KIAA1549L*** | ↑ in all three datasets |  |  | *MYO5B* | ↓in all three datasets |
| ***RAB9A*** | ↑ in all three datasets |  |  | *ACAA1* | ↓in all three datasets |
| ***ZC2HC1A*** | ↑ in all three datasets |  |  | *AIG1* | ↓in all three datasets |
| ***KIRREL3*** | ↑ in all three datasets |  |  | *TLE1* | ↓in all three datasets |
| ***GDI1*** | ↑ in all three datasets |  |  | *ARHGEF10L* | ↓in all three datasets |
| ***LAGE3*** | ↑ in all three datasets |  |  | *AK3* | ↓in all three datasets |
| ***GPR176*** | ↑ in all three datasets |  |  | *GATA4* | ↓in all three datasets |
| ***RHOC*** | ↑ in all three datasets |  |  | *LRRC61* | ↓in all three datasets |
| ***BAG3*** | ↑ in all three datasets |  |  | *YIF1A* | ↓in all three datasets |
| ***SSX2IP*** | ↑ in all three datasets |  |  | *TAB3* | ↓in all three datasets |
| ***PEA15*** | ↑ in all three datasets |  |  | *GAMT* | ↓in all three datasets |
| ***MTMR11*** | ↑ in all three datasets |  |  | *POR* | ↓in all three datasets |
| ***BTG3*** | ↑ in all three datasets |  |  | *MEP1A* | ↓in all three datasets |
| ***PRR5L*** | ↑ in all three datasets |  |  | *SMOC1* | ↓in all three datasets |
| ***IL1RAPL1*** | ↑ in all three datasets |  |  | *SOWAHA* | ↓in all three datasets |
| ***ARHGAP26*** | ↑ in all three datasets |  |  | *SLC39A14* | ↓in all three datasets |
| ***IFI27L2*** | ↑ in all three datasets |  |  | *TBC1D8B* | ↓in all three datasets |
| ***MRC2*** | ↑ in all three datasets |  |  | *C15orf39* | ↓in all three datasets |
| ***ITPRIP*** | ↑ in all three datasets |  |  | *EHHADH* | ↓in all three datasets |
| ***JOSD2*** | ↑ in all three datasets |  |  | *AMN* | ↓in all three datasets |
| ***SIRPA*** | ↑ in all three datasets |  |  | *NIPSNAP1* | ↓in all three datasets |
| ***RGMB*** | ↑ in all three datasets |  |  | *CNOT11* | ↓in all three datasets |
| ***CFAP36*** | ↑ in all three datasets |  |  | *PCSK6* | ↓in all three datasets |
| ***CDKN2C*** | ↑ in all three datasets |  |  | *STXBP2* | ↓in all three datasets |
| ***TMEM218*** | ↑ in all three datasets |  |  | *XYLB* | ↓in all three datasets |
| ***SLK*** | ↑ in all three datasets |  |  | *ZFP36L2* | ↓in all three datasets |
| ***RPS6KA5*** | ↑ in all three datasets |  |  | *PROZ* | ↓in all three datasets |
| ***STX11*** | ↑ in all three datasets |  |  | *PRLR* | ↓in all three datasets |
| ***SMG9*** | ↑ in all three datasets |  |  | *ANKRD46* | ↓in all three datasets |
| ***HKDC1*** | ↑ in all three datasets |  |  | *PDLIM5* | ↓in all three datasets |
| ***TNFRSF10A*** | ↑ in all three datasets |  |  | *RHOF* | ↓in all three datasets |
| ***PLEC*** | ↑ in all three datasets |  |  | *PCYT2* | ↓in all three datasets |
| ***PDZD11*** | ↑ in all three datasets |  |  | *TOM1* | ↓in all three datasets |
| ***BAD*** | ↑ in all three datasets |  |  | *SPATA13* | ↓in all three datasets |
| ***N6AMT1*** | ↑ in all three datasets |  |  | *ADD3* | ↓in all three datasets |
| ***CD59*** | ↑ in all three datasets |  |  | *COPE* | ↓in all three datasets |
| ***DYRK3*** | ↑ in all three datasets |  |  | *MRPL30* | ↓in all three datasets |
| ***NOTCH2*** | ↑ in all three datasets |  |  | *DLX6* | ↓in all three datasets |
| ***IRF9*** | ↑ in all three datasets |  |  | *DIP2B* | ↓in all three datasets |
| ***TLDC1*** | ↑ in all three datasets |  |  | *NR2F6* | ↓in all three datasets |
| ***WDR41*** | ↑ in all three datasets |  |  | *PIK3R1* | ↓in all three datasets |
| ***TMX3*** | ↑ in all three datasets |  |  | *NPHP4* | ↓in all three datasets |
| ***CD274*** | ↑ in all three datasets |  |  | *HNF4A* | ↓in all three datasets |
| ***MACF1*** | ↑ in all three datasets |  |  | *QPRT* | ↓in all three datasets |
| ***TUFT1*** | ↑ in all three datasets |  |  | *SHMT2* | ↓in all three datasets |
| ***IRAK1*** | ↑ in all three datasets |  |  | *PTPN6* | ↓in all three datasets |
| ***CEP290*** | ↑ in all three datasets |  |  | *SPTBN1* | ↓in all three datasets |
| ***GAS6*** | ↑ in all three datasets |  |  | *MXD1* | ↓in all three datasets |
| ***TCEAL4*** | ↑ in all three datasets |  |  | *PGM3* | ↓in all three datasets |
| ***EMD*** | ↑ in all three datasets |  |  | *TTC6* | ↓in all three datasets |
| ***NEDD4*** | ↑ in all three datasets |  |  | *MYRF* | ↓in all three datasets |
| ***DNAAF3*** | ↑ in all three datasets |  |  | *IL1R2* | ↓in all three datasets |
| ***RBPMS*** | ↑ in all three datasets |  |  | *TMEM39A* | ↓in all three datasets |
| ***CEP250*** | ↑ in all three datasets |  |  | *ADI1* | ↓in all three datasets |
| ***SPTLC2*** | ↑ in all three datasets |  |  | *IDH1* | ↓in all three datasets |
| ***ARL6IP5*** | ↑ in all three datasets |  |  | *TP53INP1* | ↓in all three datasets |
| ***NIN*** | ↑ in all three datasets |  |  | *HMG20A* | ↓in all three datasets |
| ***PAX9*** | ↑ in all three datasets |  |  | *RMND5A* | ↓in all three datasets |
| ***MIF4GD*** | ↑ in all three datasets |  |  | *LYSMD4* | ↓in all three datasets |
| ***EHD3*** | ↑ in all three datasets |  |  | *GUF1* | ↓in all three datasets |
| ***TRDMT1*** | ↑ in all three datasets |  |  | *COL9A2* | ↓in all three datasets |
| ***NAA10*** | ↑ in all three datasets |  |  | *ALAD* | ↓in all three datasets |
| ***CD151*** | ↑ in all three datasets |  |  | *PEBP1* | ↓in all three datasets |
| ***TMEM107*** | ↑ in all three datasets |  |  | *TP53INP2* | ↓in all three datasets |
| ***C6orf1*** | ↑ in all three datasets |  |  | *CCDC117* | ↓in all three datasets |
| ***ACOT9*** | ↑ in all three datasets |  |  | *TPST1* | ↓in all three datasets |
| ***PPP1R13L*** | ↑ in all three datasets |  |  | *PECR* | ↓in all three datasets |
| ***AMZ2*** | ↑ in all three datasets |  |  | *GBA2* | ↓in all three datasets |
| ***SFXN3*** | ↑ in all three datasets |  |  | *SV2A* | ↓in all three datasets |
| ***PIFO*** | ↑ in all three datasets |  |  | *MAP3K8* | ↓in all three datasets |
| ***NR3C1*** | ↑ in all three datasets |  |  | *SLC25A1* | ↓in all three datasets |
| ***FAM229B*** | ↑ in all three datasets |  |  | *CRKL* | ↓in all three datasets |
| ***APOBEC3F*** | ↑ in all three datasets |  |  | *DCAF12* | ↓in all three datasets |
| ***RNF145*** | ↑ in all three datasets |  |  | *MPST* | ↓in all three datasets |
| ***THG1L*** | ↑ in all three datasets |  |  | *CROCCP2* | ↓in all three datasets |
| ***EHBP1L1*** | ↑ in all three datasets |  |  | *SLC6A19* | ↓in all three datasets |
| ***TSPAN17*** | ↑ in all three datasets |  |  | *NPAS2* | ↓in all three datasets |
| ***RASSF8*** | ↑ in all three datasets |  |  | *GRAMD4* | ↓in all three datasets |
| ***OPTN*** | ↑ in all three datasets |  |  | *SMARCA1* | ↓in all three datasets |
| ***IFIT2*** | ↑ in all three datasets |  |  | *NCBP2-AS2* | ↓in all three datasets |
| ***CCDC102A*** | ↑ in all three datasets |  |  | *TMEM216* | ↓in all three datasets |
| ***CAMLG*** | ↑ in all three datasets |  |  | *FAXDC2* | ↓in all three datasets |
| ***RASA1*** | ↑ in all three datasets |  |  | *UBA5* | ↓in all three datasets |
| ***CAST*** | ↑ in all three datasets |  |  | *ATP6V1A* | ↓in all three datasets |
| ***ADAM12*** | ↑ in all three datasets |  |  | *GCDH* | ↓in all three datasets |
| ***FAM171B*** | ↑ in all three datasets |  |  | *TRAF4* | ↓in all three datasets |
| ***GABBR1*** | ↑ in all three datasets |  |  | *SEPHS2* | ↓in all three datasets |
| ***SHKBP1*** | ↑ in all three datasets |  |  | *PIGN* | ↓in all three datasets |
| ***PIGK*** | ↑ in all three datasets |  |  | *SLC2A8* | ↓in all three datasets |
| ***MASTL*** | ↑ in all three datasets |  |  | *SNX4* | ↓in all three datasets |
| ***EVA1B*** | ↑ in all three datasets |  |  | *HIGD1A* | ↓in all three datasets |
| ***ZNF569*** | ↑ in all three datasets |  |  | *TSN* | ↓in all three datasets |
| ***FAM3A*** | ↑ in all three datasets |  |  | *PDXDC1* | ↓in all three datasets |
| ***PIAS3*** | ↑ in all three datasets |  |  | *PHOSPHO2* | ↓in all three datasets |
| ***CBR1*** | ↑ in all three datasets |  |  | *MARVELD3* | ↓in all three datasets |
| ***ARHGAP31*** | ↑ in all three datasets |  |  | *RORA* | ↓in all three datasets |
| ***BBS10*** | ↑ in all three datasets |  |  | *LMAN1* | ↓in all three datasets |
| ***C11orf68*** | ↑ in all three datasets |  |  | *CENPV* | ↓in all three datasets |
| ***MED7*** | ↑ in all three datasets |  |  | *PSME4* | ↓in all three datasets |
| ***ARL16*** | ↑ in all three datasets |  |  | *MOGS* | ↓in all three datasets |
| ***SLC39A6*** | ↑ in all three datasets |  |  | *KDM7A* | ↓in all three datasets |
| ***NME7*** | ↑ in all three datasets |  |  | *BCL2L11* | ↓in all three datasets |
| ***UBTD1*** | ↑ in all three datasets |  |  | *ATF7IP2* | ↓in all three datasets |
| ***KLHL36*** | ↑ in all three datasets |  |  | *PEX13* | ↓in all three datasets |
| ***TTC7B*** | ↑ in all three datasets |  |  | *TBX2* | ↓in all three datasets |
| ***ARHGAP21*** | ↑ in all three datasets |  |  | *IMMP1L* | ↓in all three datasets |
| ***LRTOMT*** | ↑ in all three datasets |  |  | *SORBS1* | ↓in all three datasets |
| ***CMTR2*** | ↑ in all three datasets |  |  | *C2orf88* | ↓in all three datasets |
| ***KATNAL1*** | ↑ in all three datasets |  |  | *ATF2* | ↓in all three datasets |
| ***MORN2*** | ↑ in all three datasets |  |  | *BEND3* | ↓in all three datasets |
| ***KCTD18*** | ↑ in all three datasets |  |  | *STRN3* | ↓in all three datasets |
| ***FAM50A*** | ↑ in all three datasets |  |  | *THPO* | ↓in all three datasets |
| ***PRKCA*** | ↑ in all three datasets |  |  | *EPHB4* | ↓in all three datasets |
| ***C11orf1*** | ↑ in all three datasets |  |  | *BTG2* | ↓in all three datasets |
| ***PNMA1*** | ↑ in all three datasets |  |  | *R3HDM4* | ↓in all three datasets |
| ***EFHC1*** | ↑ in all three datasets |  |  | *LGALSL* | ↓in all three datasets |
| ***CEP19*** | ↑ in all three datasets |  |  | *PSPH* | ↓in all three datasets |
| ***ERC1*** | ↑ in all three datasets |  |  | *CCDC28A* | ↓in all three datasets |
| ***SLC10A3*** | ↑ in all three datasets |  |  | *ZCCHC3* | ↓in all three datasets |
| ***ALKBH2*** | ↑ in all three datasets |  |  | *DTNA* | ↓in all three datasets |
| ***DCLK2*** | ↑ in all three datasets |  |  | *EPB41L4B* | ↓in all three datasets |
| ***CRYZL1*** | ↑ in all three datasets |  |  | *OSBPL3* | ↓in all three datasets |
| ***GNPDA2*** | ↑ in all three datasets |  |  | *DAP* | ↓in all three datasets |
| ***FAM161A*** | ↑ in all three datasets |  |  | *PGPEP1* | ↓in all three datasets |
| ***CPNE2*** | ↑ in all three datasets |  |  | *C1RL* | ↓in all three datasets |
| ***PTPRJ*** | ↑ in all three datasets |  |  | *HSD17B11* | ↓in all three datasets |
| ***APOO*** | ↑ in all three datasets |  |  | *ACVR2B* | ↓in all three datasets |
| ***RHOQ*** | ↑ in all three datasets |  |  | *C1orf131* | ↓in all three datasets |
| ***BCL9L*** | ↑ in all three datasets |  |  | *ETFA* | ↓in all three datasets |
| ***TRIM21*** | ↑ in all three datasets |  |  | *MIEF1* | ↓in all three datasets |
| ***FAT4*** | ↑ in all three datasets |  |  | *SARDH* | ↓in all three datasets |
| ***IL31RA*** | ↑ in all three datasets |  |  | *SURF4* | ↓in all three datasets |
| ***CLUAP1*** | ↑ in all three datasets |  |  | *SSBP3* | ↓in all three datasets |
| ***SMAD3*** | ↑ in all three datasets |  |  | *NCOA4* | ↓in all three datasets |
| ***IFT22*** | ↑ in all three datasets |  |  | *NDUFA13* | ↓in all three datasets |
| ***CEP89*** | ↑ in all three datasets |  |  | *UGGT1* | ↓in all three datasets |
| ***ALG13*** | ↑ in all three datasets |  |  | *EMILIN2* | ↓in all three datasets |
| ***WDR34*** | ↑ in all three datasets |  |  | *SPOPL* | ↓in all three datasets |
| ***ZNF280C*** | ↑ in all three datasets |  |  | *ACAT2* | ↓in all three datasets |
| ***DEGS1*** | ↑ in all three datasets |  |  | *RREB1* | ↓in all three datasets |
| ***ERCC1*** | ↑ in all three datasets |  |  | *MAP2K4* | ↓in all three datasets |
| ***ARL3*** | ↑ in all three datasets |  |  | *CLDN12* | ↓in all three datasets |
| ***RFESD*** | ↑ in all three datasets |  |  | *RABL3* | ↓in all three datasets |
| ***SP100*** | ↑ in all three datasets |  |  | *LSM7* | ↓in all three datasets |
| ***CYTH3*** | ↑ in all three datasets |  |  | *LHPP* | ↓in all three datasets |
| ***UBQLN2*** | ↑ in all three datasets |  |  | *TRMT10A* | ↓in all three datasets |
| ***FDXACB1*** | ↑ in all three datasets |  |  | *MTSS1* | ↓in all three datasets |
| ***DNAJC6*** | ↑ in all three datasets |  |  | *PLEKHM3* | ↓in all three datasets |
| ***CSF2RA*** | ↑ in all three datasets |  |  | *SEC13* | ↓in all three datasets |
| ***ZAK*** | ↑ in all three datasets |  |  | *FUCA2* | ↓in all three datasets |
| ***MET*** | ↑ in all three datasets |  |  | *MRPS25* | ↓in all three datasets |
| ***RNF11*** | ↑ in all three datasets |  |  | *PPP6R2* | ↓in all three datasets |
| ***NF2*** | ↑ in all three datasets |  |  | *FLVCR1* | ↓in all three datasets |
| ***CCDC85B*** | ↑ in all three datasets |  |  | *ZNF518A* | ↓in all three datasets |
| ***LMNA*** | ↑ in all three datasets |  |  | *RPH3AL* | ↓in all three datasets |
| ***ZMYM6*** | ↑ in all three datasets |  |  | *MAST3* | ↓in all three datasets |
| ***JAK1*** | ↑ in all three datasets |  |  | *TMEM182* | ↓in all three datasets |
| ***NUDT2*** | ↑ in all three datasets |  |  | *EDC3* | ↓in all three datasets |
| ***POLK*** | ↑ in all three datasets |  |  | *NIPBL* | ↓in all three datasets |
| ***CAMSAP2*** | ↑ in all three datasets |  |  | *EIF4EBP2* | ↓in all three datasets |
| ***ARMCX5*** | ↑ in all three datasets |  |  | *FBXL12* | ↓in all three datasets |
| ***TRPS1*** | ↑ in all three datasets |  |  | *IMP3* | ↓in all three datasets |
| ***PPP3CB*** | ↑ in all three datasets |  |  | *MRPL46* | ↓in all three datasets |
| ***TBC1D19*** | ↑ in all three datasets |  |  | *MPHOSPH10* | ↓in all three datasets |
| ***BMPR2*** | ↑ in all three datasets |  |  | *RANBP2* | ↓in all three datasets |
| ***GBX2*** | ↑ in all three datasets |  |  | *RNF38* | ↓in all three datasets |
| ***MID2*** | ↑ in all three datasets |  |  | *DNMT3A* | ↓in all three datasets |
| ***ZNF879*** | ↑ in all three datasets |  |  | *CKAP4* | ↓in all three datasets |
| ***CHRNA5*** | ↑ in all three datasets |  |  | *SLC43A2* | ↓in all three datasets |
| ***ODF2L*** | ↑ in all three datasets |  |  | *JAGN1* | ↓in all three datasets |
| ***CCSER2*** | ↑ in all three datasets |  |  | *ADPGK* | ↓in all three datasets |
| ***GLRX2*** | ↑ in all three datasets |  |  | *CLSTN3* | ↓in all three datasets |
| ***STK17B*** | ↑ in all three datasets |  |  | *C1QTNF3* | ↓in all three datasets |
| ***ZBTB25*** | ↑ in all three datasets |  |  | *UPF1* | ↓in all three datasets |
| ***FAM127A*** | ↑ in all three datasets |  |  | *VWA9* | ↓in all three datasets |
| ***BCL10*** | ↑ in all three datasets |  |  | *CPLX1* | ↓in all three datasets |
| ***TRERF1*** | ↑ in all three datasets |  |  | *MRPL50* | ↓in all three datasets |
| ***SHCBP1*** | ↑ in all three datasets |  |  | *PIK3R2* | ↓in all three datasets |
| ***PTCD2*** | ↑ in all three datasets |  |  | *SLC44A1* | ↓in all three datasets |
| ***ANXA2P1*** | ↑ in all three datasets |  |  | *MAU2* | ↓in all three datasets |
| ***TEAD1*** | ↑ in all three datasets |  |  | *AGFG2* | ↓in all three datasets |
| ***PYGB*** | ↑ in all three datasets |  |  | *PIF1* | ↓in all three datasets |
| ***CRIM1*** | ↑ in all three datasets |  |  | *UMPS* | ↓in all three datasets |
| ***HSPB11*** | ↑ in all three datasets |  |  | *GREB1* | ↓in all three datasets |
| ***DSTN*** | ↑ in all three datasets |  |  | *SH3PXD2A* | ↓in all three datasets |
| ***AP4B1*** | ↑ in all three datasets |  |  | *SRPRB* | ↓in all three datasets |
| ***REEP3*** | ↑ in all three datasets |  |  | *FDPS* | ↓in all three datasets |
| ***PXN*** | ↑ in all three datasets |  |  | *ANKH* | ↓in all three datasets |
| ***OGT*** | ↑ in all three datasets |  |  | *ELAVL1* | ↓in all three datasets |
| ***ORAI2*** | ↑ in all three datasets |  |  | *SEC31A* | ↓in all three datasets |
| ***LARP6*** | ↑ in all three datasets |  |  | *RBM14* | ↓in all three datasets |
| ***BBIP1*** | ↑ in all three datasets |  |  | *SLC22A23* | ↓in all three datasets |
| ***CYYR1*** | ↑ in all three datasets |  |  | *SLC17A5* | ↓in all three datasets |
| ***GJA3*** | ↑ in all three datasets |  |  | *TFRC* | ↓in all three datasets |
| ***PAFAH1B3*** | ↑ in all three datasets |  |  | *SOX9-AS1* | ↓in all three datasets |
| ***ZNF584*** | ↑ in all three datasets |  |  | *SND1-IT1* | ↓in all three datasets |
| ***AHNAK*** | ↑ in all three datasets |  |  | *ANP32A* | ↓in all three datasets |
| ***DPCD*** | ↑ in all three datasets |  |  | *SENP7* | ↓in all three datasets |
| ***RBFOX2*** | ↑ in all three datasets |  |  | *HDHD2* | ↓in all three datasets |
| ***VBP1*** | ↑ in all three datasets |  |  | *TMEM97* | ↓in all three datasets |
| ***AP2B1*** | ↑ in all three datasets |  |  | *HNRNPC* | ↓in all three datasets |
| ***MAP3K12*** | ↑ in all three datasets |  |  | *TPRN* | ↓in all three datasets |
| ***FAM129B*** | ↑ in all three datasets |  |  | *PMF1* | ↓in all three datasets |
| ***USP33*** | ↑ in all three datasets |  |  | *CCDC138* | ↓in all three datasets |
| ***GNAI2*** | ↑ in all three datasets |  |  | *CHP1* | ↓in all three datasets |
| ***TRIO*** | ↑ in all three datasets |  |  | *CYB5R3* | ↓in all three datasets |
| ***ZEB2*** | ↑ in all three datasets |  |  | *SFXN5* | ↓in all three datasets |
| ***DDX50*** | ↑ in all three datasets |  |  | *GOLGA2P5* | ↓in all three datasets |
| ***VANGL1*** | ↑ in all three datasets |  |  | *DANCR* | ↓in all three datasets |
| ***COQ2*** | ↑ in all three datasets |  |  | *ABCD3* | ↓in all three datasets |
| ***SGPP1*** | ↑ in all three datasets |  |  | *NDUFA11* | ↓in all three datasets |
| ***FUBP1*** | ↑ in all three datasets |  |  | *UBXN4* | ↓in all three datasets |
| ***RPS6KA4*** | ↑ in all three datasets |  |  | *DOLPP1* | ↓in all three datasets |
| ***ZFAND4*** | ↑ in all three datasets |  |  | *ARIH1* | ↓in all three datasets |
| ***NOC3L*** | ↑ in all three datasets |  |  | *ATG13* | ↓in all three datasets |
| ***USP32*** | ↑ in all three datasets |  |  | *TIA1* | ↓in all three datasets |
| ***ELOVL5*** | ↑ in all three datasets |  |  | *ABHD10* | ↓in all three datasets |
| ***C2CD3*** | ↑ in all three datasets |  |  | *UBE2N* | ↓in all three datasets |
| ***DZANK1*** | ↑ in all three datasets |  |  | *SIPA1L1* | ↓in all three datasets |
| ***RRAGC*** | ↑ in all three datasets |  |  | *EBLN2* | ↓in all three datasets |
| ***STPG1*** | ↑ in all three datasets |  |  | *NGRN* | ↓in all three datasets |
| ***TTC12*** | ↑ in all three datasets |  |  | *OXNAD1* | ↓in all three datasets |
| ***PKMYT1*** | ↑ in all three datasets |  |  | *RALGAPA2* | ↓in all three datasets |
| ***SHISA4*** | ↑ in all three datasets |  |  | *SLC46A1* | ↓in all three datasets |
| ***ANXA11*** | ↑ in all three datasets |  |  | *CLDN15* | ↓in all three datasets |
| ***ANKIB1*** | ↑ in all three datasets |  |  | *SLC25A42* | ↓in all three datasets |
| ***ZBTB8A*** | ↑ in all three datasets |  |  | *FAM20A* | ↓in all three datasets |
| ***WNT2B*** | ↑ in all three datasets |  |  | *GUSBP11* | ↓in all three datasets |
| ***RDM1*** | ↑ in all three datasets |  |  | *LMTK2* | ↓in all three datasets |
| ***PHF21A*** | ↑ in all three datasets |  |  | *TCF7* | ↓in all three datasets |
| ***MORN4*** | ↑ in all three datasets |  |  | *BTD* | ↓in all three datasets |
| ***TAZ*** | ↑ in all three datasets |  |  | *SHROOM1* | ↓in all three datasets |
| ***STK3*** | ↑ in all three datasets |  |  | *NUP50* | ↓in all three datasets |
| ***PKN2*** | ↑ in all three datasets |  |  | *GOLGB1* | ↓in all three datasets |
| ***MRPS6*** | ↑ in all three datasets |  |  | *C19orf25* | ↓in all three datasets |
| ***ACTR1A*** | ↑ in all three datasets |  |  | *ZBTB46* | ↓in all three datasets |
| ***RASA3*** | ↑ in all three datasets |  |  | *ERP44* | ↓in all three datasets |
| ***NEXN-AS1*** | ↑ in all three datasets |  |  | *HAAO* | ↓in all three datasets |
| ***ZDHHC15*** | ↑ in all three datasets |  |  | *TUBD1* | ↓in all three datasets |
| ***CEP128*** | ↑ in all three datasets |  |  | *AKAP8L* | ↓in all three datasets |
| ***CAPNS1*** | ↑ in all three datasets |  |  | *MAN2C1* | ↓in all three datasets |
| ***SPAG9*** | ↑ in all three datasets |  |  | *MRPL47* | ↓in all three datasets |
| ***HEATR6*** | ↑ in all three datasets |  |  | *PDS5B* | ↓in all three datasets |
| ***ZNF337*** | ↑ in all three datasets |  |  | *POLDIP3* | ↓in all three datasets |
| ***MRPS16*** | ↑ in all three datasets |  |  | *NDUFAF5* | ↓in all three datasets |
| ***UBE2A*** | ↑ in all three datasets |  |  | *PIAS1* | ↓in all three datasets |
| ***ZNF436*** | ↑ in all three datasets |  |  | *MIDN* | ↓in all three datasets |
| ***MAP7D3*** | ↑ in all three datasets |  |  | *ZSCAN25* | ↓in all three datasets |
| ***AKR1E2*** | ↑ in all three datasets |  |  | *ZG16* | ↓in all three datasets |
| ***SOCS3*** | ↑ in all three datasets |  |  | *DECR2* | ↓in all three datasets |
| ***ADAMTSL1*** | ↑ in all three datasets |  |  | *KLF15* | ↓in all three datasets |
| ***SLC48A1*** | ↑ in all three datasets |  |  | *GATAD2A* | ↓in all three datasets |
| ***FOXJ2*** | ↑ in all three datasets |  |  | *MRPS14* | ↓in all three datasets |
| ***CAP1*** | ↑ in all three datasets |  |  | *CNBP* | ↓in all three datasets |
| ***POLR2L*** | ↑ in all three datasets |  |  | *PHF5A* | ↓in all three datasets |
| ***EPG5*** | ↑ in all three datasets |  |  | *ASB7* | ↓in all three datasets |
| ***RNF213*** | ↑ in all three datasets |  |  | *TRMT10C* | ↓in all three datasets |
| ***DHX40*** | ↑ in all three datasets |  |  | *SLC25A25* | ↓in all three datasets |
| ***STARD3NL*** | ↑ in all three datasets |  |  | *DOCK6* | ↓in all three datasets |
| ***LAMA3*** | ↑ in all three datasets |  |  | *SLC25A13* | ↓in all three datasets |
| ***ZNF585A*** | ↑ in all three datasets |  |  | *ITGAL* | ↓in all three datasets |
| ***AGGF1*** | ↑ in all three datasets |  |  | *DAGLA* | ↓in all three datasets |
| ***MBOAT7*** | ↑ in all three datasets |  |  | *LRP6* | ↓in all three datasets |
| ***FAM160B1*** | ↑ in all three datasets |  |  | *FOXP4* | ↓in all three datasets |
| ***MANBAL*** | ↑ in all three datasets |  |  | *TNS1* | ↓in all three datasets |
| ***COPRS*** | ↑ in all three datasets |  |  | *TULP4* | ↓in all three datasets |
| ***PARVA*** | ↑ in all three datasets |  |  | *PPIG* | ↓in all three datasets |
| ***RIN1*** | ↑ in all three datasets |  |  | *TADA3* | ↓in all three datasets |
| ***COL4A3BP*** | ↑ in all three datasets |  |  | *HMGN3* | ↓in all three datasets |
| ***UTP14A*** | ↑ in all three datasets |  |  | *CHERP* | ↓in all three datasets |
| ***TRIM7*** | ↑ in all three datasets |  |  | *RANBP3* | ↓in all three datasets |
| ***MICAL3*** | ↑ in all three datasets |  |  | *OTULIN* | ↓in all three datasets |
| ***BCAP29*** | ↑ in all three datasets |  |  | *AKAP8* | ↓in all three datasets |
| ***CAMK2G*** | ↑ in all three datasets |  |  | *TMEM242* | ↓in all three datasets |
| ***ATP6V1D*** | ↑ in all three datasets |  |  | *FAM32A* | ↓in all three datasets |
| ***RBFA*** | ↑ in all three datasets |  |  | *SENP5* | ↓in all three datasets |
| ***TRIML2*** | ↑ in all three datasets |  |  | *PAPOLA* | ↓in all three datasets |
| ***MECP2*** | ↑ in all three datasets |  |  | *ZXDC* | ↓in all three datasets |
| ***ZBTB4*** | ↑ in all three datasets |  |  | *APTX* | ↓in all three datasets |
| ***DUSP7*** | ↑ in all three datasets |  |  | *RAB26* | ↓in all three datasets |
| ***HPS6*** | ↑ in all three datasets |  |  | *MAP3K13* | ↓in all three datasets |
| ***TCEB2*** | ↑ in all three datasets |  |  | *UBD///GABBR1* | ↓ in two datasets |
| ***LPCAT4*** | ↑ in all three datasets |  |  | *ORM2///ORM1* | ↓ in two datasets |
| ***CCDC96*** | ↑ in all three datasets |  |  | *LYZ* | ↓ in two datasets |
| ***NAT14*** | ↑ in all three datasets |  |  | *CP* | ↓ in two datasets |
| ***IQGAP1*** | ↑ in all three datasets |  |  | *DLK1* | ↓ in two datasets |
| ***NEK11*** | ↑ in all three datasets |  |  | *S100P* | ↓ in two datasets |
| ***HFE*** | ↑ in all three datasets |  |  | *KISS1R* | ↓ in two datasets |
| ***FSIP1*** | ↑ in all three datasets |  |  | *HAMP* | ↓ in two datasets |
| ***MAP4K5*** | ↑ in all three datasets |  |  | *RELN* | ↓ in two datasets |
| ***WASF2*** | ↑ in all three datasets |  |  | *RARRES2* | ↓ in two datasets |
| ***RNASEL*** | ↑ in all three datasets |  |  | *ANGPTL3* | ↓ in two datasets |
| ***RBBP7*** | ↑ in all three datasets |  |  | *SERPINI1* | ↓ in two datasets |
| ***TRIM37*** | ↑ in all three datasets |  |  | *CYBA* | ↓ in two datasets |
| ***LMLN*** | ↑ in all three datasets |  |  | *HPD* | ↓ in two datasets |
| ***EPS15*** | ↑ in all three datasets |  |  | *TESC* | ↓ in two datasets |
| ***CEP164*** | ↑ in all three datasets |  |  | *AGTR1* | ↓ in two datasets |
| ***ZNF585B*** | ↑ in all three datasets |  |  | *EPS8L3* | ↓ in two datasets |
| ***FOSL2*** | ↑ in all three datasets |  |  | *CTHRC1* | ↓ in two datasets |
| ***TP53BP1*** | ↑ in all three datasets |  |  | *HYAL1* | ↓ in two datasets |
| ***PGRMC2*** | ↑ in all three datasets |  |  | *CEACAM1* | ↓ in two datasets |
| ***SYDE1*** | ↑ in all three datasets |  |  | *SPRY1* | ↓ in two datasets |
| ***THAP9-AS1*** | ↑ in all three datasets |  |  | *LPAR6* | ↓ in two datasets |
| ***MBTPS2*** | ↑ in all three datasets |  |  | *TIMP3* | ↓ in two datasets |
| ***FAM204A*** | ↑ in all three datasets |  |  | *IGSF1* | ↓ in two datasets |
| ***ARF6*** | ↑ in all three datasets |  |  | *SLC29A3* | ↓ in two datasets |
| ***SOCS5*** | ↑ in all three datasets |  |  | *ALDH8A1* | ↓ in two datasets |
| ***MDGA1*** | ↑ in all three datasets |  |  | *ERP27* | ↓ in two datasets |
| ***RUVBL2*** | ↑ in all three datasets |  |  | *MCC* | ↓ in two datasets |
| ***LINC01137*** | ↑ in all three datasets |  |  | *TMEM86B* | ↓ in two datasets |
| ***TSEN15*** | ↑ in all three datasets |  |  | *DTX4* | ↓ in two datasets |
| ***RNH1*** | ↑ in all three datasets |  |  | *PAQR9* | ↓ in two datasets |
| ***TTC21A*** | ↑ in all three datasets |  |  | *CRIP1* | ↓ in two datasets |
| ***KMT2A*** | ↑ in all three datasets |  |  | *LIN7A* | ↓ in two datasets |
| ***EZH1*** | ↑ in all three datasets |  |  | *PLLP* | ↓ in two datasets |
| ***ASPHD2*** | ↑ in all three datasets |  |  | *FAM13C* | ↓ in two datasets |
| ***HARS2*** | ↑ in all three datasets |  |  | *PLEKHB1* | ↓ in two datasets |
| ***GIT1*** | ↑ in all three datasets |  |  | *BHMT2* | ↓ in two datasets |
| ***FAM214B*** | ↑ in all three datasets |  |  | *GSAP* | ↓ in two datasets |
| ***ZNF449*** | ↑ in all three datasets |  |  | *LGR5* | ↓ in two datasets |
| ***CTNNA1*** | ↑ in all three datasets |  |  | *C1orf115* | ↓ in two datasets |
| ***TBC1D1*** | ↑ in all three datasets |  |  | *TNFRSF19* | ↓ in two datasets |
| ***IQSEC2*** | ↑ in all three datasets |  |  | *SKP2* | ↓ in two datasets |
| ***POLR2I*** | ↑ in all three datasets |  |  | *PALMD* | ↓ in two datasets |
| ***VPS29*** | ↑ in all three datasets |  |  | *JAG1* | ↓ in two datasets |
| ***PAM*** | ↑ in all three datasets |  |  | *NADK2* | ↓ in two datasets |
| ***TBC1D9B*** | ↑ in all three datasets |  |  | *GYG2* | ↓ in two datasets |
| ***STAG2*** | ↑ in all three datasets |  |  | *NAALAD2* | ↓ in two datasets |
| ***ZNF568*** | ↑ in all three datasets |  |  | *MMD* | ↓ in two datasets |
| ***DNTTIP2*** | ↑ in all three datasets |  |  | *PLD1* | ↓ in two datasets |
| ***ZNF619*** | ↑ in all three datasets |  |  | *SULT1C2* | ↓ in two datasets |
| ***EPHA2*** | ↑ in all three datasets |  |  | *GPC6* | ↓ in two datasets |
| ***ATP6V0E1*** | ↑ in all three datasets |  |  | *SHANK2* | ↓ in two datasets |
| ***YWHAB*** | ↑ in all three datasets |  |  | *FRAT1* | ↓ in two datasets |
| ***USP51*** | ↑ in all three datasets |  |  | *SALL2* | ↓ in two datasets |
| ***RHOT1*** | ↑ in all three datasets |  |  | *TBC1D4* | ↓ in two datasets |
| ***NUMA1*** | ↑ in all three datasets |  |  | *ETNPPL* | ↓ in two datasets |
| ***GNA13*** | ↑ in all three datasets |  |  | *DMD* | ↓ in two datasets |
| ***AATF*** | ↑ in all three datasets |  |  | *HNF4G* | ↓ in two datasets |
| ***HOXB5*** | ↑ in all three datasets |  |  | *STAP2* | ↓ in two datasets |
| ***BEST3*** | ↑ in all three datasets |  |  | *TNFAIP8L1* | ↓ in two datasets |
| ***PRMT2*** | ↑ in all three datasets |  |  | *SPRY4* | ↓ in two datasets |
| ***ITGB3*** | ↑ in all three datasets |  |  | *FRMD3* | ↓ in two datasets |
| ***C8orf46*** | ↑ in all three datasets |  |  | *NDRG2* | ↓ in two datasets |
| ***VPS26B*** | ↑ in all three datasets |  |  | *SEMA3G* | ↓ in two datasets |
| ***CYSTM1*** | ↑ in all three datasets |  |  | *TMEM178A* | ↓ in two datasets |
| ***FAM208A*** | ↑ in all three datasets |  |  | *PUS10* | ↓ in two datasets |
| ***ZMYND11*** | ↑ in all three datasets |  |  | *TNNC1* | ↓ in two datasets |
| ***MGAT5B*** | ↑ in all three datasets |  |  | *FCGRT* | ↓ in two datasets |
| ***TTLL6*** | ↑ in all three datasets |  |  | *TUBBP5* | ↓ in two datasets |
| ***VPS26A*** | ↑ in all three datasets |  |  | *SLC16A6* | ↓ in two datasets |
| ***NCOR2*** | ↑ in all three datasets |  |  | *TFR2* | ↓ in two datasets |
| ***VIM*** | ↑ in two datasets |  |  | *PEG10* | ↓ in two datasets |
| ***IL6*** | ↑ in two datasets |  |  | *ANXA4* | ↓ in two datasets |
| ***TMSB4X*** | ↑ in two datasets |  |  | *SULT1E1* | ↓ in two datasets |
| ***LOC105379362*** | ↑ in two datasets |  |  | *EML6* | ↓ in two datasets |
| ***IFI44*** | ↑ in two datasets |  |  | *VPS54* | ↓ in two datasets |
| ***LIF*** | ↑ in two datasets |  |  | *SLC7A9* | ↓ in two datasets |
| ***ENO2*** | ↑ in two datasets |  |  | *IFNGR1* | ↓ in two datasets |
| ***LY6K*** | ↑ in two datasets |  |  | *ISX* | ↓ in two datasets |
| ***GPNMB*** | ↑ in two datasets |  |  | *TBX3* | ↓ in two datasets |
| ***ELK3*** | ↑ in two datasets |  |  | *ZNF516* | ↓ in two datasets |
| ***MIR100HG*** | ↑ in two datasets |  |  | *GPD1L* | ↓ in two datasets |
| ***GAPLINC*** | ↑ in two datasets |  |  | *CRACR2B* | ↓ in two datasets |
| ***IL18*** | ↑ in two datasets |  |  | *NINJ2* | ↓ in two datasets |
| ***MCTP1*** | ↑ in two datasets |  |  | *ALDH4A1* | ↓ in two datasets |
| ***P3H2*** | ↑ in two datasets |  |  | *THRB* | ↓ in two datasets |
| ***PRPS2*** | ↑ in two datasets |  |  | *SESTD1* | ↓ in two datasets |
| ***DPYSL3*** | ↑ in two datasets |  |  | *SOX6* | ↓ in two datasets |
| ***MT1X*** | ↑ in two datasets |  |  | *RXRA* | ↓ in two datasets |
| ***MT1E*** | ↑ in two datasets |  |  | *MCF2L-AS1* | ↓ in two datasets |
| ***SPIN4*** | ↑ in two datasets |  |  | *OPLAH* | ↓ in two datasets |
| ***HMGA2*** | ↑ in two datasets |  |  | *SLC27A3* | ↓ in two datasets |
| ***PFKFB3*** | ↑ in two datasets |  |  | *TMEM140* | ↓ in two datasets |
| ***SLC30A4*** | ↑ in two datasets |  |  | *CCDC69* | ↓ in two datasets |
| ***ARHGAP23*** | ↑ in two datasets |  |  | *HEY1* | ↓ in two datasets |
| ***BAGE*** | ↑ in two datasets |  |  | *RHOB* | ↓ in two datasets |
| ***AMFR*** | ↑ in two datasets |  |  | *TMEM141* | ↓ in two datasets |
| ***MT2A*** | ↑ in two datasets |  |  | *EFEMP2* | ↓ in two datasets |
| ***FOSB*** | ↑ in two datasets |  |  | *LRP1* | ↓ in two datasets |
| ***CARMIL1*** | ↑ in two datasets |  |  | *NCOA2* | ↓ in two datasets |
| ***TMEM178B*** | ↑ in two datasets |  |  | *BHMT* | ↓ in two datasets |
| ***ABR*** | ↑ in two datasets |  |  | *PTCD3* | ↓ in two datasets |
| ***STC2*** | ↑ in two datasets |  |  | *FAM214A* | ↓ in two datasets |
| ***KLHL29*** | ↑ in two datasets |  |  | *ERMP1* | ↓ in two datasets |
| ***MTMR1*** | ↑ in two datasets |  |  | *FGD4* | ↓ in two datasets |
| ***NHS*** | ↑ in two datasets |  |  | *PELI1* | ↓ in two datasets |
| ***SLC25A37*** | ↑ in two datasets |  |  | *VWA8* | ↓ in two datasets |
| ***MT1H*** | ↑ in two datasets |  |  | *ADCY9* | ↓ in two datasets |
| ***IFT27*** | ↑ in two datasets |  |  | *SMAD9* | ↓ in two datasets |
| ***IFT57*** | ↑ in two datasets |  |  | *CBS* | ↓ in two datasets |
| ***CD47*** | ↑ in two datasets |  |  | *DRAM1* | ↓ in two datasets |
| ***HDAC9*** | ↑ in two datasets |  |  | *PPP4R4* | ↓ in two datasets |
| ***FAM92A1*** | ↑ in two datasets |  |  | *KIF13B* | ↓ in two datasets |
| ***DUSP1*** | ↑ in two datasets |  |  | *ZNF764* | ↓ in two datasets |
| ***B4GALT6*** | ↑ in two datasets |  |  | *NPR2* | ↓ in two datasets |
| ***TGFB2*** | ↑ in two datasets |  |  | *NINJ1* | ↓ in two datasets |
| ***SH3BP5*** | ↑ in two datasets |  |  | *PLXND1* | ↓ in two datasets |
| ***MCUB*** | ↑ in two datasets |  |  | *KLF13* | ↓ in two datasets |
| ***KLF7*** | ↑ in two datasets |  |  | *YPEL2* | ↓ in two datasets |
| ***APBB2*** | ↑ in two datasets |  |  | *FAM118A* | ↓ in two datasets |
| ***RIMKLB*** | ↑ in two datasets |  |  | *IL13RA1* | ↓ in two datasets |
| ***DPY19L2*** | ↑ in two datasets |  |  | *ZCCHC24* | ↓ in two datasets |
| ***ARMC9*** | ↑ in two datasets |  |  | *SNAP23* | ↓ in two datasets |
| ***LOC100126784*** | ↑ in two datasets |  |  | *VAMP8* | ↓ in two datasets |
| ***C15orf52*** | ↑ in two datasets |  |  | *TMC6* | ↓ in two datasets |
| ***PPP1R15A*** | ↑ in two datasets |  |  | *SLC4A11* | ↓ in two datasets |
| ***IL15*** | ↑ in two datasets |  |  | *SEC24D* | ↓ in two datasets |
| ***PLEK2*** | ↑ in two datasets |  |  | *AKAP1* | ↓ in two datasets |
| ***BOD1L1*** | ↑ in two datasets |  |  | *GOLIM4* | ↓ in two datasets |
| ***TRIM38*** | ↑ in two datasets |  |  | *MRAS* | ↓ in two datasets |
| ***PTBP2*** | ↑ in two datasets |  |  | *ISYNA1* | ↓ in two datasets |
| ***SH2B3*** | ↑ in two datasets |  |  | *CHD7* | ↓ in two datasets |
| ***NT5E*** | ↑ in two datasets |  |  | *CCNB1IP1* | ↓ in two datasets |
| ***ULBP2*** | ↑ in two datasets |  |  | *IDH2* | ↓ in two datasets |
| ***COL8A1*** | ↑ in two datasets |  |  | *EIF2AK3* | ↓ in two datasets |
| ***TBX18*** | ↑ in two datasets |  |  | *LPIN2* | ↓ in two datasets |
| ***MICB*** | ↑ in two datasets |  |  | *SLAIN1* | ↓ in two datasets |
| ***MDFIC*** | ↑ in two datasets |  |  | *TMTC4* | ↓ in two datasets |
| ***SS18*** | ↑ in two datasets |  |  | *SOWAHC* | ↓ in two datasets |
| ***SMS*** | ↑ in two datasets |  |  | *ZMAT3* | ↓ in two datasets |
| ***LOC101928955*** | ↑ in two datasets |  |  | *PLSCR4* | ↓ in two datasets |
| ***GLIDR*** | ↑ in two datasets |  |  | *ZNF253* | ↓ in two datasets |
| ***MYO10*** | ↑ in two datasets |  |  | *TMEM2* | ↓ in two datasets |
| ***PTPRD*** | ↑ in two datasets |  |  | *MOGAT3* | ↓ in two datasets |
| ***CPNE8*** | ↑ in two datasets |  |  | *TXNDC16* | ↓ in two datasets |
| ***LTBP4*** | ↑ in two datasets |  |  | *IRF2BP2* | ↓ in two datasets |
| ***OASL*** | ↑ in two datasets |  |  | *FEM1A* | ↓ in two datasets |
| ***KIF1BP*** | ↑ in two datasets |  |  | *SERP1* | ↓ in two datasets |
| ***ITGB1*** | ↑ in two datasets |  |  | *SH3TC1* | ↓ in two datasets |
| ***TMEM185A*** | ↑ in two datasets |  |  | *KLF9* | ↓ in two datasets |
| ***MYL12A*** | ↑ in two datasets |  |  | *CLYBL* | ↓ in two datasets |
| ***PHKA1*** | ↑ in two datasets |  |  | *UGGT2* | ↓ in two datasets |
| ***ZNF365*** | ↑ in two datasets |  |  | *DTD2* | ↓ in two datasets |
| ***PRKACB*** | ↑ in two datasets |  |  | *LZTS3* | ↓ in two datasets |
| ***KCTD1*** | ↑ in two datasets |  |  | *FNTA* | ↓ in two datasets |
| ***CHM*** | ↑ in two datasets |  |  | *COG3* | ↓ in two datasets |
| ***RNF14*** | ↑ in two datasets |  |  | *LOC100506314* | ↓ in two datasets |
| ***BNIP3L*** | ↑ in two datasets |  |  | *SMCO4* | ↓ in two datasets |
| ***GPR180*** | ↑ in two datasets |  |  | *SEC23A* | ↓ in two datasets |
| ***DNAJC18*** | ↑ in two datasets |  |  | *LEO1* | ↓ in two datasets |
| ***NABP1*** | ↑ in two datasets |  |  | *RMND1* | ↓ in two datasets |
| ***MT1G*** | ↑ in two datasets |  |  | *MBTD1* | ↓ in two datasets |
| ***RIPPLY2*** | ↑ in two datasets |  |  | *UXS1* | ↓ in two datasets |
| ***SWAP70*** | ↑ in two datasets |  |  | *CCDC50* | ↓ in two datasets |
| ***FZD6*** | ↑ in two datasets |  |  | *HDHD3* | ↓ in two datasets |
| ***ZC3HAV1*** | ↑ in two datasets |  |  | *HIRA* | ↓ in two datasets |
| ***AFF4*** | ↑ in two datasets |  |  | *MCCC1* | ↓ in two datasets |
| ***NRAV*** | ↑ in two datasets |  |  | *TRMT11* | ↓ in two datasets |
| ***HIVEP3*** | ↑ in two datasets |  |  | *LMBRD1* | ↓ in two datasets |
| ***FGD6*** | ↑ in two datasets |  |  | *CCNJ* | ↓ in two datasets |
| ***ARRDC3*** | ↑ in two datasets |  |  | *IFRD1* | ↓ in two datasets |
| ***STK10*** | ↑ in two datasets |  |  | *CEBPG* | ↓ in two datasets |
| ***PRELID3B*** | ↑ in two datasets |  |  | *FMO1* | ↓ in two datasets |
| ***MALAT1*** | ↑ in two datasets |  |  | *ZFPM1* | ↓ in two datasets |
| ***AFAP1*** | ↑ in two datasets |  |  | *FAM8A1* | ↓ in two datasets |
| ***RAB22A*** | ↑ in two datasets |  |  | *BAG1* | ↓ in two datasets |
| ***RLIM*** | ↑ in two datasets |  |  | *SPHAR///RAB4A* | ↓ in two datasets |
| ***CD99L2*** | ↑ in two datasets |  |  | *LYRM1* | ↓ in two datasets |
| ***NCS1*** | ↑ in two datasets |  |  | *TGDS* | ↓ in two datasets |
| ***ELK1*** | ↑ in two datasets |  |  | *NFE2L2* | ↓ in two datasets |
| ***CELF2*** | ↑ in two datasets |  |  | *DPP8* | ↓ in two datasets |
| ***LINC01111*** | ↑ in two datasets |  |  | *ATP2B2* | ↓ in two datasets |
| ***ANKRD20A5P*** | ↑ in two datasets |  |  | *KIF1C* | ↓ in two datasets |
| ***PARD6B*** | ↑ in two datasets |  |  | *UBN2* | ↓ in two datasets |
| ***SLC4A7*** | ↑ in two datasets |  |  | *PLEKHG6* | ↓ in two datasets |
| ***SAMD12*** | ↑ in two datasets |  |  | *CDC42EP1* | ↓ in two datasets |
| ***ARHGEF28*** | ↑ in two datasets |  |  | *NDUFA7* | ↓ in two datasets |
| ***NBN*** | ↑ in two datasets |  |  | *NFXL1* | ↓ in two datasets |
| ***PLEKHM1*** | ↑ in two datasets |  |  | *N4BP2L1* | ↓ in two datasets |
| ***HPSE*** | ↑ in two datasets |  |  | *PSAT1* | ↓ in two datasets |
| ***WDR76*** | ↑ in two datasets |  |  | *CDIP1* | ↓ in two datasets |
| ***SSH1*** | ↑ in two datasets |  |  | *GGCX* | ↓ in two datasets |
| ***FAF1*** | ↑ in two datasets |  |  | *FAM169A* | ↓ in two datasets |
| ***APOLD1*** | ↑ in two datasets |  |  | *CD46* | ↓ in two datasets |
| ***ADAM9*** | ↑ in two datasets |  |  | *ZBTB5* | ↓ in two datasets |
| ***CYB5R4*** | ↑ in two datasets |  |  | *TRAPPC6B* | ↓ in two datasets |
| ***CCDC53*** | ↑ in two datasets |  |  | *MAPK6* | ↓ in two datasets |
| ***VMP1*** | ↑ in two datasets |  |  | *PCCB* | ↓ in two datasets |
| ***TRIM25*** | ↑ in two datasets |  |  | *EEFSEC* | ↓ in two datasets |
| ***ERBIN*** | ↑ in two datasets |  |  | *CTDSPL* | ↓ in two datasets |
| ***RAD50*** | ↑ in two datasets |  |  | *REL* | ↓ in two datasets |
| ***TBCD*** | ↑ in two datasets |  |  | *ELF1* | ↓ in two datasets |
| ***NAA15*** | ↑ in two datasets |  |  | *PHKA2* | ↓ in two datasets |
| ***BBS7*** | ↑ in two datasets |  |  | *TGIF2* | ↓ in two datasets |
| ***EFCAB14*** | ↑ in two datasets |  |  | *SDHC* | ↓ in two datasets |
| ***AGTRAP*** | ↑ in two datasets |  |  | *SLC1A3* | ↓ in two datasets |
| ***RAB29*** | ↑ in two datasets |  |  | *LINC00597* | ↓ in two datasets |
| ***TTC37*** | ↑ in two datasets |  |  | *IPO5* | ↓ in two datasets |
| ***RASAL2*** | ↑ in two datasets |  |  | *SLC37A4* | ↓ in two datasets |
| ***MYEF2*** | ↑ in two datasets |  |  | *FAH* | ↓ in two datasets |
| ***LIX1L*** | ↑ in two datasets |  |  | *FXN* | ↓ in two datasets |
| ***DPY19L1*** | ↑ in two datasets |  |  | *PDSS2* | ↓ in two datasets |
| ***CCL28*** | ↑ in two datasets |  |  | *PDE8A* | ↓ in two datasets |
| ***AMOTL1*** | ↑ in two datasets |  |  | *ITM2C* | ↓ in two datasets |
| ***WHSC1*** | ↑ in two datasets |  |  | *TCAIM* | ↓ in two datasets |
| ***WDR44*** | ↑ in two datasets |  |  | *CD74* | ↓ in two datasets |
| ***TNPO1*** | ↑ in two datasets |  |  | *SMIM14* | ↓ in two datasets |
| ***TMEM65*** | ↑ in two datasets |  |  | *CNKSR1* | ↓ in two datasets |
| ***DSTYK*** | ↑ in two datasets |  |  | *GGTLC1* | ↓ in two datasets |
| ***ALPK2*** | ↑ in two datasets |  |  | *MPND* | ↓ in two datasets |
| ***ERLIN2*** | ↑ in two datasets |  |  | *LMO7* | ↓ in two datasets |
| ***ELMO2*** | ↑ in two datasets |  |  | *MEF2A* | ↓ in two datasets |
| ***TMTC3*** | ↑ in two datasets |  |  | *BCKDHA* | ↓ in two datasets |
| ***SPIN3*** | ↑ in two datasets |  |  | *SLC39A11* | ↓ in two datasets |
| ***PKIG*** | ↑ in two datasets |  |  | *NCK2* | ↓ in two datasets |
| ***LINC01588*** | ↑ in two datasets |  |  | *SPRED2* | ↓ in two datasets |
| ***ARNTL2*** | ↑ in two datasets |  |  | *MFSD3* | ↓ in two datasets |
| ***AMZ2P1*** | ↑ in two datasets |  |  | *POGLUT1* | ↓ in two datasets |
| ***TKT*** | ↑ in two datasets |  |  | *TMEM98* | ↓ in two datasets |
| ***LHFP*** | ↑ in two datasets |  |  | *SCNN1A* | ↓ in two datasets |
| ***IRF1*** | ↑ in two datasets |  |  | *ALDH3A2* | ↓ in two datasets |
| ***HCCS*** | ↑ in two datasets |  |  | *GALNT13* | ↓ in two datasets |
| ***EIF5A2*** | ↑ in two datasets |  |  | *OSTC* | ↓ in two datasets |
| ***RUBCN*** | ↑ in two datasets |  |  | *DVL3* | ↓ in two datasets |
| ***DOCK1*** | ↑ in two datasets |  |  | *GSPT1* | ↓ in two datasets |
| ***CREBL2*** | ↑ in two datasets |  |  | *SEPSECS* | ↓ in two datasets |
| ***NLE1*** | ↑ in two datasets |  |  | *GSDMB* | ↓ in two datasets |
| ***BTBD10*** | ↑ in two datasets |  |  | *HS2ST1* | ↓ in two datasets |
| ***AHI1*** | ↑ in two datasets |  |  | *SLC26A3* | ↓ in two datasets |
| ***LOC105379426*** | ↑ in two datasets |  |  | *ARHGEF16* | ↓ in two datasets |
| ***RBMS2*** | ↑ in two datasets |  |  | *BRAF* | ↓ in two datasets |
| ***HPRT1*** | ↑ in two datasets |  |  | *P4HB* | ↓ in two datasets |
| ***SQSTM1*** | ↑ in two datasets |  |  | *PDIA4* | ↓ in two datasets |
| ***FBXW11*** | ↑ in two datasets |  |  | *CTNNBIP1* | ↓ in two datasets |
| ***SEMA3E*** | ↑ in two datasets |  |  | *PLOD3* | ↓ in two datasets |
| ***PSAP*** | ↑ in two datasets |  |  | *BRD1* | ↓ in two datasets |
| ***DUBR*** | ↑ in two datasets |  |  | *ATAD2B* | ↓ in two datasets |
| ***CHML*** | ↑ in two datasets |  |  | *KEAP1* | ↓ in two datasets |
| ***RAB13*** | ↑ in two datasets |  |  | *ACSL3* | ↓ in two datasets |
| ***ELF4*** | ↑ in two datasets |  |  | *MAGEF1* | ↓ in two datasets |
| ***UEVLD*** | ↑ in two datasets |  |  | *GXYLT1* | ↓ in two datasets |
| ***SCPEP1*** | ↑ in two datasets |  |  | *FOXP1* | ↓ in two datasets |
| ***SYNE2*** | ↑ in two datasets |  |  | *UPF3A* | ↓ in two datasets |
| ***CTBS*** | ↑ in two datasets |  |  | *ACP5* | ↓ in two datasets |
| ***RCAN3*** | ↑ in two datasets |  |  | *GCC2* | ↓ in two datasets |
| ***PPP1R9B*** | ↑ in two datasets |  |  | *PDIA3* | ↓ in two datasets |
| ***ACTN1*** | ↑ in two datasets |  |  | *FCER1G* | ↓ in two datasets |
| ***YWHAZ*** | ↑ in two datasets |  |  | *LARP1B* | ↓ in two datasets |
| ***NSMAF*** | ↑ in two datasets |  |  | *KDM4B* | ↓ in two datasets |
| ***HTR7P1*** | ↑ in two datasets |  |  | *NFKB1* | ↓ in two datasets |
| ***CDC42*** | ↑ in two datasets |  |  | *HSD17B4* | ↓ in two datasets |
| ***XIAP*** | ↑ in two datasets |  |  | *ATXN7L3B* | ↓ in two datasets |
| ***SPRY2*** | ↑ in two datasets |  |  | *PROSER1* | ↓ in two datasets |
| ***HAUS7*** | ↑ in two datasets |  |  | *PEPD* | ↓ in two datasets |
| ***GPATCH11*** | ↑ in two datasets |  |  | *TIMM44* | ↓ in two datasets |
| ***TTC26*** | ↑ in two datasets |  |  | *GORASP2* | ↓ in two datasets |
| ***TNIP1*** | ↑ in two datasets |  |  | *ARID4A* | ↓ in two datasets |
| ***MGC70870*** | ↑ in two datasets |  |  | *UTRN* | ↓ in two datasets |
| ***PHTF2*** | ↑ in two datasets |  |  | *RNF44* | ↓ in two datasets |
| ***GALNT2*** | ↑ in two datasets |  |  | *TMEM169* | ↓ in two datasets |
| ***FKBP15*** | ↑ in two datasets |  |  | *EXOC6* | ↓ in two datasets |
| ***ATRNL1*** | ↑ in two datasets |  |  | *CIR1* | ↓ in two datasets |
| ***TMEM50B*** | ↑ in two datasets |  |  | *ST13* | ↓ in two datasets |
| ***ZFYVE1*** | ↑ in two datasets |  |  | *GALT* | ↓ in two datasets |
| ***RBM33*** | ↑ in two datasets |  |  | *ZSCAN2* | ↓ in two datasets |
| ***PER3*** | ↑ in two datasets |  |  | *C9orf64* | ↓ in two datasets |
| ***NRG2*** | ↑ in two datasets |  |  | *SEMA6B* | ↓ in two datasets |
| ***MRPL43*** | ↑ in two datasets |  |  | *CPT2* | ↓ in two datasets |
| ***NPHP1*** | ↑ in two datasets |  |  | *CHD2* | ↓ in two datasets |
| ***KLF10*** | ↑ in two datasets |  |  | *SEC62* | ↓ in two datasets |
| ***DST*** | ↑ in two datasets |  |  | *CLASP1* | ↓ in two datasets |
| ***ANXA2*** | ↑ in two datasets |  |  | *TMEM41A* | ↓ in two datasets |
| ***MICAL2*** | ↑ in two datasets |  |  | *ISL2* | ↓ in two datasets |
| ***FUT4*** | ↑ in two datasets |  |  | *GFM1* | ↓ in two datasets |
| ***CDC42SE2*** | ↑ in two datasets |  |  | *DGCR2* | ↓ in two datasets |
| ***C11orf63*** | ↑ in two datasets |  |  | *SBNO1* | ↓ in two datasets |
| ***ZNF438*** | ↑ in two datasets |  |  | *AGPS* | ↓ in two datasets |
| ***VAMP7*** | ↑ in two datasets |  |  | *SEMA4C* | ↓ in two datasets |
| ***TMEM185B*** | ↑ in two datasets |  |  | *EFCAB12* | ↓ in two datasets |
| ***PSCA*** | ↑ in two datasets |  |  | *GPX4* | ↓ in two datasets |
| ***FAM216A*** | ↑ in two datasets |  |  | *UGP2* | ↓ in two datasets |
| ***CYLD*** | ↑ in two datasets |  |  | *LRRC8D* | ↓ in two datasets |
| ***CREBZF*** | ↑ in two datasets |  |  | *DBI* | ↓ in two datasets |
| ***SRR*** | ↑ in two datasets |  |  | *RPL32P3* | ↓ in two datasets |
| ***SNPH*** | ↑ in two datasets |  |  | *LPCAT3* | ↓ in two datasets |
| ***MOB3A*** | ↑ in two datasets |  |  | *SMPX* | ↓ in two datasets |
| ***CCDC82*** | ↑ in two datasets |  |  | *KPNA1* | ↓ in two datasets |
| ***RNF115*** | ↑ in two datasets |  |  | *ATG4B* | ↓ in two datasets |
| ***KCTD11*** | ↑ in two datasets |  |  | *NUDT16* | ↓ in two datasets |
| ***HS1BP3*** | ↑ in two datasets |  |  | *TPCN1* | ↓ in two datasets |
| ***DIEXF*** | ↑ in two datasets |  |  | *CDC34* | ↓ in two datasets |
| ***BCL2*** | ↑ in two datasets |  |  | *PACSIN3* | ↓ in two datasets |
| ***WDR1*** | ↑ in two datasets |  |  | *USP8* | ↓ in two datasets |
| ***SMKR1*** | ↑ in two datasets |  |  | *CALML4* | ↓ in two datasets |
| ***LOC339803*** | ↑ in two datasets |  |  | *NIFK* | ↓ in two datasets |
| ***FCHO2*** | ↑ in two datasets |  |  | *ARGLU1* | ↓ in two datasets |
| ***RSF1*** | ↑ in two datasets |  |  | *MRPS30* | ↓ in two datasets |
| ***NLN*** | ↑ in two datasets |  |  | *CSK* | ↓ in two datasets |
| ***HEIH*** | ↑ in two datasets |  |  | *SRSF3* | ↓ in two datasets |
| ***ZNF578*** | ↑ in two datasets |  |  | *SCAPER* | ↓ in two datasets |
| ***RRP12*** | ↑ in two datasets |  |  | *ABHD17B* | ↓ in two datasets |
| ***MIER1*** | ↑ in two datasets |  |  | *CDC37L1* | ↓ in two datasets |
| ***DGKA*** | ↑ in two datasets |  |  | *THAP9* | ↓ in two datasets |
| ***CCDC6*** | ↑ in two datasets |  |  | *METTL8* | ↓ in two datasets |
| ***ARHGEF12*** | ↑ in two datasets |  |  | *WDR81* | ↓ in two datasets |
| ***ANKRD13B*** | ↑ in two datasets |  |  | *POLR1B* | ↓ in two datasets |
| ***RAD51B*** | ↑ in two datasets |  |  | *LAMTOR3* | ↓ in two datasets |
| ***PANX1*** | ↑ in two datasets |  |  | *FGFRL1* | ↓ in two datasets |
| ***CYTH2*** | ↑ in two datasets |  |  | *CIRBP* | ↓ in two datasets |
| ***TSR3*** | ↑ in two datasets |  |  | *LIAS* | ↓ in two datasets |
| ***STARD13*** | ↑ in two datasets |  |  | *TFB2M* | ↓ in two datasets |
| ***MIGA1*** | ↑ in two datasets |  |  | *DCP2* | ↓ in two datasets |
| ***LOC100129406*** | ↑ in two datasets |  |  | *TJP3* | ↓ in two datasets |
| ***FOXJ3*** | ↑ in two datasets |  |  | *SENP2* | ↓ in two datasets |
| ***ABCE1*** | ↑ in two datasets |  |  | *FBXO22* | ↓ in two datasets |
| ***NR1H2*** | ↑ in two datasets |  |  | *NMD3* | ↓ in two datasets |
| ***MTMR2*** | ↑ in two datasets |  |  | *SEC63* | ↓ in two datasets |
| ***DLL3*** | ↑ in two datasets |  |  | *TRIM33* | ↓ in two datasets |
| ***CRLF3*** | ↑ in two datasets |  |  | *MAGI1* | ↓ in two datasets |
| ***PICALM*** | ↑ in two datasets |  |  | *SPATA5L1* | ↓ in two datasets |
| ***FPGT*** | ↑ in two datasets |  |  | *QSOX2* | ↓ in two datasets |
| ***ADAM17*** | ↑ in two datasets |  |  | *SMAD4* | ↓ in two datasets |
| ***SMARCC2*** | ↑ in two datasets |  |  | *ADAT2* | ↓ in two datasets |
| ***PGAP1*** | ↑ in two datasets |  |  | *SCP2* | ↓ in two datasets |
| ***ZXDA*** | ↑ in two datasets |  |  | *HCG18* | ↓ in two datasets |
| ***S1PR5*** | ↑ in two datasets |  |  | *WDR91* | ↓ in two datasets |
| ***LSM11*** | ↑ in two datasets |  |  | *TSPYL1* | ↓ in two datasets |
| ***KIF1B*** | ↑ in two datasets |  |  | *PPP1R2* | ↓ in two datasets |
| ***DCLRE1C*** | ↑ in two datasets |  |  | *SUPT20H* | ↓ in two datasets |
| ***BCO1*** | ↑ in two datasets |  |  | *MTO1* | ↓ in two datasets |
| ***SLC29A1*** | ↑ in two datasets |  |  | *TP73-AS1* | ↓ in two datasets |
| ***RRAS2*** | ↑ in two datasets |  |  | *EEF2* | ↓ in two datasets |
| ***PPP1R14B*** | ↑ in two datasets |  |  | *C2orf68* | ↓ in two datasets |
| ***METTL21B*** | ↑ in two datasets |  |  | *TAF4* | ↓ in two datasets |
| ***MTCP1*** | ↑ in two datasets |  |  | *NDUFB5* | ↓ in two datasets |
| ***DNAH5*** | ↑ in two datasets |  |  | *PCGF3* | ↓ in two datasets |
| ***LARS*** | ↑ in two datasets |  |  | *AQR* | ↓ in two datasets |
| ***TNFAIP8*** | ↑ in two datasets |  |  | *MAML1* | ↓ in two datasets |
| ***BCAR1*** | ↑ in two datasets |  |  | *JDP2* | ↓ in two datasets |
| ***LINC00998*** | ↑ in two datasets |  |  | *ANAPC5* | ↓ in two datasets |
| ***YBX3*** | ↑ in two datasets |  |  | *TNPO2* | ↓ in two datasets |
| ***CXorf38*** | ↑ in two datasets |  |  | *ZMYM2* | ↓ in two datasets |
| ***APOPT1*** | ↑ in two datasets |  |  | *NFX1* | ↓ in two datasets |
| ***PYGO1*** | ↑ in two datasets |  |  | *LRCH3* | ↓ in two datasets |
| ***CXorf40B*** | ↑ in two datasets |  |  | *HUNK* | ↓ in two datasets |
| ***ENO1*** | ↑ in two datasets |  |  | *PLXNA1* | ↓ in two datasets |
| ***ADTRP*** | ↑ in two datasets |  |  | *RNF41* | ↓ in two datasets |
| ***BRCC3*** | ↑ in two datasets |  |  | *FBXW4P1* | ↓ in two datasets |
| ***KLHL42*** | ↑ in two datasets |  |  | *UPB1* | ↓ in two datasets |
| ***IER2*** | ↑ in two datasets |  |  | *ZNF770* | ↓ in two datasets |
| ***SNX12*** | ↑ in two datasets |  |  | *RAB3GAP1* | ↓ in two datasets |
| ***NDFIP1*** | ↑ in two datasets |  |  | *TSPAN9* | ↓ in two datasets |
| ***PRPF40B*** | ↑ in two datasets |  |  | *POLR2E* | ↓ in two datasets |
| ***ANK1*** | ↑ in two datasets |  |  | *RIC1* | ↓ in two datasets |
| ***COL17A1*** | ↑ in two datasets |  |  | *GMIP* | ↓ in two datasets |
| ***ARAF*** | ↑ in two datasets |  |  | *MDH2* | ↓ in two datasets |
| ***YLPM1*** | ↑ in two datasets |  |  | *PAPOLG* | ↓ in two datasets |
| ***VAMP3*** | ↑ in two datasets |  |  | *TRPM7* | ↓ in two datasets |
| ***COPS6*** | ↑ in two datasets |  |  | *RNF103* | ↓ in two datasets |
| ***UBE2M*** | ↑ in two datasets |  |  | *C1QTNF6* | ↓ in two datasets |
| ***UBR7*** | ↑ in two datasets |  |  | *MGST1* | ↓ in two datasets |
| ***RPL26L1*** | ↑ in two datasets |  |  | *FXR1* | ↓ in two datasets |
| ***GPR161*** | ↑ in two datasets |  |  | *C1orf53* | ↓ in two datasets |
| ***SLC30A5*** | ↑ in two datasets |  |  | *DNAJC19* | ↓ in two datasets |
| ***GM2A*** | ↑ in two datasets |  |  | *COX5A* | ↓ in two datasets |
| ***LRRC37B*** | ↑ in two datasets |  |  | *TOX4* | ↓ in two datasets |
| ***SMAD2*** | ↑ in two datasets |  |  | *CNOT4* | ↓ in two datasets |
| ***PCDH10*** | ↑ in two datasets |  |  | *PARL* | ↓ in two datasets |
| ***ANXA2P3*** | ↑ in two datasets |  |  | *MYCN* | ↓ in two datasets |
| ***PML*** | ↑ in two datasets |  |  | *CPXM1* | ↓ in two datasets |
| ***EIF2AK2*** | ↑ in two datasets |  |  | *PAH* | ↓ in two datasets |
| ***SHOC2*** | ↑ in two datasets |  |  | *VTN* | ↓ in two datasets |
| ***PKD1L2*** | ↑ in two datasets |  |  | *GATA6* | ↓ in two datasets |
| ***AKAP10*** | ↑ in two datasets |  |  | *NAT8* | ↓ in two datasets |
| ***PLEKHM2*** | ↑ in two datasets |  |  | *CGNL1* | ↓ in two datasets |
| ***KLF11*** | ↑ in two datasets |  |  | *ESRP2* | ↓ in two datasets |
| ***CCDC189*** | ↑ in two datasets |  |  | *CCDC68* | ↓ in two datasets |
| ***KPNB1*** | ↑ in two datasets |  |  | *ABCC6* | ↓ in two datasets |
| ***MRTO4*** | ↑ in two datasets |  |  | *LINC00341* | ↓ in two datasets |
| ***YWHAQ*** | ↑ in two datasets |  |  | *OLFML3* | ↓ in two datasets |
| ***FEM1C*** | ↑ in two datasets |  |  | *SPHK1* | ↓ in two datasets |
| ***GPRIN1*** | ↑ in two datasets |  |  | *ABCB6* | ↓ in two datasets |
| ***GNB5*** | ↑ in two datasets |  |  | *FAM46A* | ↓ in two datasets |
| ***GULP1*** | ↑ in two datasets |  |  | *SLC6A14* | ↓ in two datasets |
| ***MPP5*** | ↑ in two datasets |  |  | *ABCG2* | ↓ in two datasets |
| ***DDX10*** | ↑ in two datasets |  |  | *ACTA1* | ↓ in two datasets |
| ***LOC100288181*** | ↑ in two datasets |  |  | *ACYP2* | ↓ in two datasets |
| ***MRE11A*** | ↑ in two datasets |  |  | *PGF* | ↓ in two datasets |
| ***EPHA4*** | ↑ in two datasets |  |  | *MIA2* | ↓ in two datasets |
| ***STX4*** | ↑ in two datasets |  |  | *NUDT4* | ↓ in two datasets |
| ***LSM10*** | ↑ in two datasets |  |  | *SGK223* | ↓ in two datasets |
| ***RELA*** | ↑ in two datasets |  |  | *HSD3B7* | ↓ in two datasets |
| ***PHF20*** | ↑ in two datasets |  |  | *TMEM45A* | ↓ in two datasets |
| ***ANKRD20A11P*** | ↑ in two datasets |  |  | *PDIA5* | ↓ in two datasets |
| ***JAK2*** | ↑ in two datasets |  |  | *ETS2* | ↓ in two datasets |
| ***UBL4A*** | ↑ in two datasets |  |  | *DPP3* | ↓ in two datasets |
| ***INTU*** | ↑ in two datasets |  |  | *SIK1* | ↓ in two datasets |
| ***C7orf13*** | ↑ in two datasets |  |  | *CYCS* | ↓ in two datasets |
| ***ANKS1B*** | ↑ in two datasets |  |  | *COPG1* | ↓ in two datasets |
| ***MAP3K6*** | ↑ in two datasets |  |  | *SLC20A2* | ↓ in two datasets |
| ***RHEB*** | ↑ in two datasets |  |  | *C11orf24* | ↓ in two datasets |
| ***MUC1*** | ↑ in two datasets |  |  | *MAPK14* | ↓ in two datasets |
| ***PAQR4*** | ↑ in two datasets |  |  | *PLIN2* | ↓ in two datasets |
| ***IDH3G*** | ↑ in two datasets |  |  | *CD36* | ↓ in two datasets |
| ***RECK*** | ↑ in two datasets |  |  | *TMC7* | ↓ in two datasets |
| ***DNAL1*** | ↑ in two datasets |  |  | *TOB1* | ↓ in two datasets |
| ***ERI3*** | ↑ in two datasets |  |  | *SEC61A1* | ↓ in two datasets |
| ***ANKRD36BP2*** | ↑ in two datasets |  |  | *ZBTB34* | ↓ in two datasets |
| ***LOC642852*** | ↑ in two datasets |  |  | *FUOM* | ↓ in two datasets |
| ***ATG12*** | ↑ in two datasets |  |  | *LOC728613* | ↓ in two datasets |
| ***TIRAP*** | ↑ in two datasets |  |  | *TOMM40L* | ↓ in two datasets |
| ***NRDC*** | ↑ in two datasets |  |  | *RPS6KB2* | ↓ in two datasets |
| ***CAD*** | ↑ in two datasets |  |  | *FETUB* | ↓ in two datasets |
| ***ARSD*** | ↑ in two datasets |  |  | *LONRF3* | ↓ in two datasets |
| ***SYPL1*** | ↑ in two datasets |  |  | *NAA50* | ↓ in two datasets |
| ***ELK4*** | ↑ in two datasets |  |  | *UBAC2* | ↓ in two datasets |
| ***CSNK1G3*** | ↑ in two datasets |  |  | *DDB1* | ↓ in two datasets |
| ***NDRG3*** | ↑ in two datasets |  |  | *GTF2I* | ↓ in two datasets |
| ***SYAP1*** | ↑ in two datasets |  |  | *LINC00574* | ↓ in two datasets |
| ***FLT3LG*** | ↑ in two datasets |  |  | *RNF126* | ↓ in two datasets |
| ***SRA1*** | ↑ in two datasets |  |  | *TTC21B* | ↓ in two datasets |
| ***PPIE*** | ↑ in two datasets |  |  | *UBR3* | ↓ in two datasets |
| ***NPR3*** | ↑ in two datasets |  |  | *TM9SF4* | ↓ in two datasets |
| ***TMLHE*** | ↑ in two datasets |  |  | *AJUBA* | ↓ in two datasets |
| ***RELT*** | ↑ in two datasets |  |  | *G3BP2* | ↓ in two datasets |
| ***CBL*** | ↑ in two datasets |  |  | *CALU* | ↓ in two datasets |
| ***RUFY2*** | ↑ in two datasets |  |  | *KCNQ1OT1* | ↓ in two datasets |
| ***NSMCE3*** | ↑ in two datasets |  |  | *TFF3* | ↓ in two datasets |
| ***MAST2*** | ↑ in two datasets |  |  | *SND1* | ↓ in two datasets |
| ***TAF4B*** | ↑ in two datasets |  |  | *GTPBP3* | ↓ in two datasets |
| ***MAATS1*** | ↑ in two datasets |  |  | *ACIN1* | ↓ in two datasets |
| ***MAPK11*** | ↑ in two datasets |  |  | *ZNF280A* | ↓ in two datasets |
| ***CLIP1*** | ↑ in two datasets |  |  | *ACO2* | ↓ in two datasets |
| ***ANKRD13C*** | ↑ in two datasets |  |  | *OVGP1* | ↓ in two datasets |
| ***UTP11*** | ↑ in two datasets |  |  | *PIAS4* | ↓ in two datasets |
| ***MARC9*** | ↑ in two datasets |  |  | *ARL5A* | ↓ in two datasets |
| ***ZFYVE28*** | ↑ in two datasets |  |  | *FLVCR2* | ↓ in two datasets |
| ***RUSC2*** | ↑ in two datasets |  |  | *NDUFC2* | ↓ in two datasets |
| ***ST3GAL4*** | ↑ in two datasets |  |  | *TAF11* | ↓ in two datasets |
| ***DYNC1H1*** | ↑ in two datasets |  |  | *MIR600HG* | ↓ in two datasets |
| ***AP3B1*** | ↑ in two datasets |  |  | *ZNF92* | ↓ in two datasets |
| ***ZBTB1*** | ↑ in two datasets |  |  | *CSNK1G2* | ↓ in two datasets |
| ***PDCD5*** | ↑ in two datasets |  |  | *NCLN* | ↓ in two datasets |
| ***CPEB4*** | ↑ in two datasets |  |  | *SRSF5* | ↓ in two datasets |
| ***NDUFC1*** | ↑ in two datasets |  |  | *ZNF792* | ↓ in two datasets |
| ***PTDSS1*** | ↑ in two datasets |  |  | *MPHOSPH8* | ↓ in two datasets |
| ***FAM173A*** | ↑ in two datasets |  |  | *SMARCB1* | ↓ in two datasets |
| ***PPM1F*** | ↑ in two datasets |  |  | *MCM3* | ↓ in two datasets |
| ***IKBKB*** | ↑ in two datasets |  |  | *SH3GL1* | ↓ in two datasets |
| ***CAMKK1*** | ↑ in two datasets |  |  | *TCF20* | ↓ in two datasets |
| ***VMA21*** | ↑ in two datasets |  |  | *DESI1* | ↓ in two datasets |
| ***SBF2*** | ↑ in two datasets |  |  | *STAT5A* | ↓ in two datasets |
| ***SLC25A28*** | ↑ in two datasets |  |  | *RAB14* | ↓ in two datasets |
| ***RGS9BP*** | ↑ in two datasets |  |  | *CTDSPL2* | ↓ in two datasets |
| ***MFSD14A*** | ↑ in two datasets |  |  | *PIM3* | ↓ in two datasets |
| ***SMPD2*** | ↑ in two datasets |  |  | *R3HDM1* | ↓ in two datasets |
| ***MMS19*** | ↑ in two datasets |  |  | *PTBP3* | ↓ in two datasets |
| ***EIF3I*** | ↑ in two datasets |  |  | *SALL4* | ↓ in two datasets |
| ***GALNT10*** | ↑ in two datasets |  |  | *MTMR14* | ↓ in two datasets |
| ***DENND5A*** | ↑ in two datasets |  |  | *TBP* | ↓ in two datasets |
| ***CYP2U1*** | ↑ in two datasets |  |  | *L3MBTL2* | ↓ in two datasets |
| ***ZNF343*** | ↑ in two datasets |  |  | *EPC2* | ↓ in two datasets |
| ***CERCAM*** | ↑ in two datasets |  |  | *ILF2* | ↓ in two datasets |
| ***DIP2A*** | ↑ in two datasets |  |  | *ILF3* | ↓ in two datasets |
| ***ST7L*** | ↑ in two datasets |  |  | *BCAS1* | ↓ in two datasets |
| ***TBK1*** | ↑ in two datasets |  |  | *MYO7A* | ↓ in two datasets |
| ***ROM1*** | ↑ in two datasets |  |  | *ARID5A* | ↓ in two datasets |
| ***MED14*** | ↑ in two datasets |  |  | *KHSRP* | ↓ in two datasets |
| ***UBTF*** | ↑ in two datasets |  |  | *STK11* | ↓ in two datasets |
| ***TNFRSF1A*** | ↑ in two datasets |  |  | *TARSL2* | ↓ in two datasets |
| ***PIP4K2B*** | ↑ in two datasets |  |  | *ARMC6* | ↓ in two datasets |
| ***B4GALNT1*** | ↑ in two datasets |  |  | *ANGPTL2* | ↓ in two datasets |
| ***PHAX*** | ↑ in two datasets |  |  | *MAP3K11* | ↓ in two datasets |
| ***CACNB1*** | ↑ in two datasets |  |  | *RPAP1* | ↓ in two datasets |
| ***UXT*** | ↑ in two datasets |  |  | *SH3BP1* | ↓ in two datasets |
| ***LTBR*** | ↑ in two datasets |  |  | *TRA2B* | ↓ in two datasets |
| ***LOC648987*** | ↑ in two datasets |  |  | *B3GNT8* | ↓ in two datasets |
| ***LINC01128*** | ↑ in two datasets |  |  | *PPIL2* | ↓ in two datasets |
| ***C14orf79*** | ↑ in two datasets |  |  | *USP39* | ↓ in two datasets |
| ***IFT81*** | ↑ in two datasets |  |  | *C6orf132* | ↓ in two datasets |
| ***FAM76A*** | ↑ in two datasets |  |  | *KRTCAP3* | ↓ in two datasets |
| ***BIRC2*** | ↑ in two datasets |  |  | *TRIM71* | ↓ in two datasets |
| ***VPS53*** | ↑ in two datasets |  |  | *SMLR1* | ↓ in two datasets |
| ***MAPK10*** | ↑ in two datasets |  |  | *PDZK1* | ↓ in two datasets |
| ***MKL1*** | ↑ in two datasets |  |  | *SEPP1* | ↓ in two datasets |
| ***PLCB3*** | ↑ in two datasets |  |  | *GPAM* | ↓ in two datasets |
| ***TSEN34*** | ↑ in two datasets |  |  | *FRAS1* | ↓ in two datasets |
| ***EPRS*** | ↑ in two datasets |  |  | *SLC40A1* | ↓ in two datasets |
| ***POLR3C*** | ↑ in two datasets |  |  | *EFNA2* | ↓ in two datasets |
| ***ELMSAN1*** | ↑ in two datasets |  |  | *MAOB* | ↓ in two datasets |
| ***ZNF644*** | ↑ in two datasets |  |  | *COL27A1* | ↓ in two datasets |
| ***CAPNS2*** | ↑ in two datasets |  |  | *SERPINA7* | ↓ in two datasets |
| ***UBA3*** | ↑ in two datasets |  |  | *AZGP1* | ↓ in two datasets |
| ***TEX10*** | ↑ in two datasets |  |  | *KIAA0485* | ↓ in two datasets |
| ***TIAL1*** | ↑ in two datasets |  |  | *EPPK1* | ↓ in two datasets |
| ***ARSK*** | ↑ in two datasets |  |  | *CPN2* | ↓ in two datasets |
| ***SDC3*** | ↑ in two datasets |  |  | *HP* | ↓ in two datasets |
| ***ADAMTS5*** | ↑ in two datasets |  |  | *MPZL3* | ↓ in two datasets |
| ***TK2*** | ↑ in two datasets |  |  | *C1orf106* | ↓ in two datasets |
| ***EFL1*** | ↑ in two datasets |  |  | *RNASE4* | ↓ in two datasets |
| ***LRFN3*** | ↑ in two datasets |  |  | *MPV17L* | ↓ in two datasets |
| ***PURA*** | ↑ in two datasets |  |  | *SSUH2* | ↓ in two datasets |
| ***EIF3K*** | ↑ in two datasets |  |  | *ADH4* | ↓ in two datasets |
| ***WDFY3-AS2*** | ↑ in two datasets |  |  | *SPTLC3* | ↓ in two datasets |
| ***DNAH7*** | ↑ in two datasets |  |  | *PLPP3* | ↓ in two datasets |
| ***POU2F2*** | ↑ in two datasets |  |  | *SDC2* | ↓ in two datasets |
| ***CAPZB*** | ↑ in two datasets |  |  | *EFNA1* | ↓ in two datasets |
| ***ATM*** | ↑ in two datasets |  |  | *CCEPR* | ↓ in two datasets |
| ***KIAA1109*** | ↑ in two datasets |  |  | *FMO5* | ↓ in two datasets |
| ***SPACA6*** | ↑ in two datasets |  |  | *ADGRD1* | ↓ in two datasets |
| ***PPM1M*** | ↑ in two datasets |  |  | *STRBP* | ↓ in two datasets |
| ***NBPF1*** | ↑ in two datasets |  |  | *HCAR3* | ↓ in two datasets |
| ***WDR55*** | ↑ in two datasets |  |  | *TTC39C* | ↓ in two datasets |
| ***PPIL6*** | ↑ in two datasets |  |  | *SERPINA4* | ↓ in two datasets |
| ***RUFY3*** | ↑ in two datasets |  |  | *FOXN3* | ↓ in two datasets |
| ***XRRA1*** | ↑ in two datasets |  |  | *FAM3B* | ↓ in two datasets |
| ***NOTCH1*** | ↑ in two datasets |  |  | *ACADSB* | ↓ in two datasets |
| ***PIWIL4*** | ↑ in two datasets |  |  | *ZNF703* | ↓ in two datasets |
| ***HESX1*** | ↑ in two datasets |  |  | *PLTP* | ↓ in two datasets |
| ***NF1*** | ↑ in two datasets |  |  | *DAAM1* | ↓ in two datasets |
| ***FOXK1*** | ↑ in two datasets |  |  | *ALDH6A1* | ↓ in two datasets |
| ***VLDLR-AS1*** | ↑ in two datasets |  |  | *G2E3* | ↓ in two datasets |
| ***GYPC*** | ↑ in two datasets |  |  | *LOC158402* | ↓ in two datasets |
| ***MATN2*** | ↑ in two datasets |  |  | *KLF12* | ↓ in two datasets |
| ***EML1*** | ↑ in two datasets |  |  | *MAFB* | ↓ in two datasets |
| ***LRRC8C*** | ↑ in two datasets |  |  | *SULT1A2* | ↓ in two datasets |
| ***TLE4*** | ↑ in two datasets |  |  | *AS3MT* | ↓ in two datasets |
| ***PGBD3*** | ↑ in two datasets |  |  | *TRIM10* | ↓ in two datasets |
| ***ZNF33B*** | ↑ in two datasets |  |  | *ZNF704* | ↓ in two datasets |
| ***ZBED8*** | ↑ in two datasets |  |  | *NREP* | ↓ in two datasets |
| ***USP43*** | ↑ in two datasets |  |  | *EPHX2* | ↓ in two datasets |
| ***DYNLT3*** | ↑ in two datasets |  |  | *CNIH1* | ↓ in two datasets |
| ***SNAPC1*** | ↑ in two datasets |  |  | *SP8* | ↓ in two datasets |
| ***BBS12*** | ↑ in two datasets |  |  | *RNF144A* | ↓ in two datasets |
| ***SLC3A1*** | ↑ in two datasets |  |  | *NOSTRIN* | ↓ in two datasets |
| ***PFN4*** | ↑ in two datasets |  |  | *OBSL1* | ↓ in two datasets |
| ***WDR31*** | ↑ in two datasets |  |  | *XCL1* | ↓ in two datasets |
| ***EPN2*** | ↑ in two datasets |  |  | *UNC93A* | ↓ in two datasets |
| ***HNF1B*** | ↑ in two datasets |  |  | *SERINC5* | ↓ in two datasets |
| ***HOXC8*** | ↑ in two datasets |  |  | *CPS1* | ↓ in two datasets |
| ***LOC284454*** | ↑ in two datasets |  |  | *SESN3* | ↓ in two datasets |
| ***RNF130*** | ↑ in two datasets |  |  | *MYH10* | ↓ in two datasets |
| ***EFCAB7*** | ↑ in two datasets |  |  | *KALRN* | ↓ in two datasets |
| ***HERC6*** | ↑ in two datasets |  |  | *ENPP3* | ↓ in two datasets |
| ***CCDC112*** | ↑ in two datasets |  |  | *ZNF747* | ↓ in two datasets |
| ***ZFP69*** | ↑ in two datasets |  |  | *TNRC6C* | ↓ in two datasets |
| ***SNX24*** | ↑ in two datasets |  |  | *KIAA1161* | ↓ in two datasets |
| ***GAB3*** | ↑ in two datasets |  |  | *NID2* | ↓ in two datasets |
| ***ACCS*** | ↑ in two datasets |  |  | *PIGZ* | ↓ in two datasets |
| ***UST*** | ↑ in two datasets |  |  | *PRKAB2* | ↓ in two datasets |
| ***FAM89B*** | ↑ in two datasets |  |  | *HACD2* | ↓ in two datasets |
| ***NR2F1-AS1*** | ↑ in two datasets |  |  | *EPO* | ↓ in two datasets |
| ***MYLK*** | ↑ in two datasets |  |  | *ZNF789* | ↓ in two datasets |
| ***PIH1D2*** | ↑ in two datasets |  |  | *NFYA* | ↓ in two datasets |
| ***DOCK2*** | ↑ in two datasets |  |  | *C4orf19* | ↓ in two datasets |
| ***ELOVL1*** | ↑ in two datasets |  |  | *N4BP2L2* | ↓ in two datasets |
| ***FAM58A*** | ↑ in two datasets |  |  | *ID2* | ↓ in two datasets |
| ***TMEM42*** | ↑ in two datasets |  |  | *F13B* | ↓ in two datasets |
| ***TTC8*** | ↑ in two datasets |  |  | *UPK3A* | ↓ in two datasets |
| ***ZNF571*** | ↑ in two datasets |  |  | *KIAA1958* | ↓ in two datasets |
| ***CCDC148*** | ↑ in two datasets |  |  | *BHLHA15* | ↓ in two datasets |
| ***TMPRSS15*** | ↑ in two datasets |  |  | *AFDN* | ↓ in two datasets |
| ***CCDC90B*** | ↑ in two datasets |  |  | *PLXNA2* | ↓ in two datasets |
| ***RAB11FIP2*** | ↑ in two datasets |  |  | *ETFB* | ↓ in two datasets |
| ***ZNF420*** | ↑ in two datasets |  |  | *PANK1* | ↓ in two datasets |
| ***ZC3H12C*** | ↑ in two datasets |  |  | *PPARA* | ↓ in two datasets |
| ***JADE1*** | ↑ in two datasets |  |  | *ZNF853* | ↓ in two datasets |
| ***LRRC46*** | ↑ in two datasets |  |  | *USF3* | ↓ in two datasets |
| ***RABGAP1L*** | ↑ in two datasets |  |  | *SYNE4* | ↓ in two datasets |
| ***PRDM11*** | ↑ in two datasets |  |  | *LOC107984282* | ↓ in two datasets |
| ***SAMD8*** | ↑ in two datasets |  |  | *C8B* | ↓ in two datasets |
| ***EBNA1BP2*** | ↑ in two datasets |  |  | *RBM4* | ↓ in two datasets |
| ***KBTBD3*** | ↑ in two datasets |  |  | *C10orf11* | ↓ in two datasets |
| ***KIAA0319*** | ↑ in two datasets |  |  | *RFX6* | ↓ in two datasets |
| ***PARP14*** | ↑ in two datasets |  |  | *NR6A1* | ↓ in two datasets |
| ***TCTN1*** | ↑ in two datasets |  |  | *COQ8A* | ↓ in two datasets |
| ***ZNF213*** | ↑ in two datasets |  |  | *ACSL4* | ↓ in two datasets |
| ***MRPL24*** | ↑ in two datasets |  |  | *SLC31A1* | ↓ in two datasets |
| ***ZNF383*** | ↑ in two datasets |  |  | *MTSS1L* | ↓ in two datasets |
| ***XIRP2*** | ↑ in two datasets |  |  | *NCALD* | ↓ in two datasets |
| ***BRWD3*** | ↑ in two datasets |  |  | *EPHA1* | ↓ in two datasets |
| ***LINC00525*** | ↑ in two datasets |  |  | *CDS1* | ↓ in two datasets |
| ***USP6*** | ↑ in two datasets |  |  | *MCEE* | ↓ in two datasets |
| ***AGO3*** | ↑ in two datasets |  |  | *KCNMB3* | ↓ in two datasets |
| ***GPR63*** | ↑ in two datasets |  |  | *TOR1AIP2* | ↓ in two datasets |
| ***OBFC1*** | ↑ in two datasets |  |  | *SSR1* | ↓ in two datasets |
| ***SERPINB6*** | ↑ in two datasets |  |  | *NT5DC1* | ↓ in two datasets |
| ***SULT2B1*** | ↑ in two datasets |  |  | *LOC149703* | ↓ in two datasets |
| ***GIN1*** | ↑ in two datasets |  |  | *ARFGEF3* | ↓ in two datasets |
| ***LOC389641*** | ↑ in two datasets |  |  | *ZSWIM5* | ↓ in two datasets |
| ***RBM41*** | ↑ in two datasets |  |  | *MRPL42* | ↓ in two datasets |
| ***ZNF30*** | ↑ in two datasets |  |  | *WIPI2* | ↓ in two datasets |
| ***ADAT1*** | ↑ in two datasets |  |  | *HES6* | ↓ in two datasets |
| ***KRBOX4*** | ↑ in two datasets |  |  | *MLXIPL* | ↓ in two datasets |
| ***ZNF83*** | ↑ in two datasets |  |  | *IGF2R* | ↓ in two datasets |
| ***FAM13B*** | ↑ in two datasets |  |  | *PPID* | ↓ in two datasets |
| ***NIPAL3*** | ↑ in two datasets |  |  | *MEX3A* | ↓ in two datasets |
| ***WDR78*** | ↑ in two datasets |  |  | *CDC14B* | ↓ in two datasets |
| ***EXOSC1*** | ↑ in two datasets |  |  | *B4GAT1* | ↓ in two datasets |
| ***LRRC6*** | ↑ in two datasets |  |  | *ADGRV1* | ↓ in two datasets |
| ***VAMP1*** | ↑ in two datasets |  |  | *ITIH1* | ↓ in two datasets |
| ***ZNF311*** | ↑ in two datasets |  |  | *IREB2* | ↓ in two datasets |
| ***PRKAG1*** | ↑ in two datasets |  |  | *ACTR3B* | ↓ in two datasets |
| ***AASDHPPT*** | ↑ in two datasets |  |  | *SLC51A* | ↓ in two datasets |
| ***S100PBP*** | ↑ in two datasets |  |  | *COA5* | ↓ in two datasets |
| ***TLR1*** | ↑ in two datasets |  |  | *ZKSCAN7* | ↓ in two datasets |
| ***VEZF1*** | ↑ in two datasets |  |  | *ORMDL1* | ↓ in two datasets |
| ***SIRT1*** | ↑ in two datasets |  |  | *GLUD2* | ↓ in two datasets |
| ***C1orf122*** | ↑ in two datasets |  |  | *DTX3* | ↓ in two datasets |
| ***CDK7*** | ↑ in two datasets |  |  | *PRAP1* | ↓ in two datasets |
| ***CD99P1*** | ↑ in two datasets |  |  | *GATSL2* | ↓ in two datasets |
| ***ZNF566*** | ↑ in two datasets |  |  | *TAPT1* | ↓ in two datasets |
| ***ZBTB21*** | ↑ in two datasets |  |  | *DLX6-AS1* | ↓ in two datasets |
| ***MED29*** | ↑ in two datasets |  |  | *ADAMTSL4* | ↓ in two datasets |
| ***EXT1*** | ↑ in two datasets |  |  | *ZNRF3* | ↓ in two datasets |
| ***PPM1J*** | ↑ in two datasets |  |  | *RTKN* | ↓ in two datasets |
| ***ATRX*** | ↑ in two datasets |  |  | *ZNF3* | ↓ in two datasets |
| ***OFD1*** | ↑ in two datasets |  |  | *WNK4* | ↓ in two datasets |
| ***PSORS1C1*** | ↑ in two datasets |  |  | *SUB1* | ↓ in two datasets |
| ***ZZZ3*** | ↑ in two datasets |  |  | *SAPCD2* | ↓ in two datasets |
| ***MYPN*** | ↑ in two datasets |  |  | *EAF1* | ↓ in two datasets |
| ***C5orf56*** | ↑ in two datasets |  |  | *ARRB1* | ↓ in two datasets |
| ***ZNF112*** | ↑ in two datasets |  |  | *KHK* | ↓ in two datasets |
| ***ZMYM4*** | ↑ in two datasets |  |  | *CHRD* | ↓ in two datasets |
| ***CFL1*** | ↑ in two datasets |  |  | *ASL* | ↓ in two datasets |
| ***LOC145783*** | ↑ in two datasets |  |  | *SCFD1* | ↓ in two datasets |
| ***TADA2A*** | ↑ in two datasets |  |  | *PKLR* | ↓ in two datasets |
| ***SYNPO*** | ↑ in two datasets |  |  | *CDK18* | ↓ in two datasets |
| ***C11orf49*** | ↑ in two datasets |  |  | *GABRA2* | ↓ in two datasets |
| ***LRRC73*** | ↑ in two datasets |  |  | *CREB3L3* | ↓ in two datasets |
| ***LENG8*** | ↑ in two datasets |  |  | *SORD* | ↓ in two datasets |
| ***RNF2*** | ↑ in two datasets |  |  | *BCAM* | ↓ in two datasets |
| ***HDDC2*** | ↑ in two datasets |  |  | *TMEM177* | ↓ in two datasets |
| ***THAP3*** | ↑ in two datasets |  |  | *SPOCK2* | ↓ in two datasets |
| ***MMP1*** | ↑ in two datasets |  |  | *SUSD6* | ↓ in two datasets |
| ***PRR34-AS1*** | ↑ in two datasets |  |  | *SNRPA1* | ↓ in two datasets |
| ***SRGN*** | ↑ in two datasets |  |  | *HOMER1* | ↓ in two datasets |
| ***GOLM1*** | ↑ in two datasets |  |  | *GSTM2* | ↓ in two datasets |
| ***SERPINB7*** | ↑ in two datasets |  |  | *ASF1A* | ↓ in two datasets |
| ***PRTFDC1*** | ↑ in two datasets |  |  | *SLC12A7* | ↓ in two datasets |
| ***STC1*** | ↑ in two datasets |  |  | *MMP11* | ↓ in two datasets |
| ***PRKAR2B*** | ↑ in two datasets |  |  | *LMF1* | ↓ in two datasets |
| ***PXDNL*** | ↑ in two datasets |  |  | *PCSK5* | ↓ in two datasets |
| ***CCNYL2*** | ↑ in two datasets |  |  | *SFI1* | ↓ in two datasets |
| ***MAGEA1*** | ↑ in two datasets |  |  | *RUNDC3B* | ↓ in two datasets |
| ***NOG*** | ↑ in two datasets |  |  | *GMCL1* | ↓ in two datasets |
| ***MME*** | ↑ in two datasets |  |  | *GFI1* | ↓ in two datasets |
| ***STEAP1B*** | ↑ in two datasets |  |  | *PABPN1* | ↓ in two datasets |
| ***WFDC21P*** | ↑ in two datasets |  |  | *OIP5-AS1* | ↓ in two datasets |
| ***FHL1*** | ↑ in two datasets |  |  | *ZNF189* | ↓ in two datasets |
| ***LOC100507487*** | ↑ in two datasets |  |  | *RHBG* | ↓ in two datasets |
| ***SLC22A4*** | ↑ in two datasets |  |  | *PPP2R1B* | ↓ in two datasets |
| ***TUBA1A*** | ↑ in two datasets |  |  | *NAGS* | ↓ in two datasets |
| ***MGLL*** | ↑ in two datasets |  |  | *CACFD1* | ↓ in two datasets |
| ***NDN*** | ↑ in two datasets |  |  | *FAM222A* | ↓ in two datasets |
| ***OSBPL1A*** | ↑ in two datasets |  |  | *DCAF10* | ↓ in two datasets |
| ***IGFBP4*** | ↑ in two datasets |  |  | *AGXT* | ↓ in two datasets |
| ***LINC00973*** | ↑ in two datasets |  |  | *SLC25A15* | ↓ in two datasets |
| ***MICA*** | ↑ in two datasets |  |  | *FEM1B* | ↓ in two datasets |
| ***ADAMTS1*** | ↑ in two datasets |  |  | *VAV2* | ↓ in two datasets |
| ***CEP112*** | ↑ in two datasets |  |  | *ANKS4B* | ↓ in two datasets |
| ***MAP3K5*** | ↑ in two datasets |  |  | *JAML* | ↓ in two datasets |
| ***MLPH*** | ↑ in two datasets |  |  | *DEF6* | ↓ in two datasets |
| ***MGC24103*** | ↑ in two datasets |  |  | *TCAF1* | ↓ in two datasets |
| ***FAM46B*** | ↑ in two datasets |  |  | *LCOR* | ↓ in two datasets |
| ***LOC100130938*** | ↑ in two datasets |  |  | *ARMC8* | ↓ in two datasets |
| ***QPCT*** | ↑ in two datasets |  |  | *ACOX1* | ↓ in two datasets |
| ***IFIT1*** | ↑ in two datasets |  |  | *ST20-AS1* | ↓ in two datasets |
| ***FAM102B*** | ↑ in two datasets |  |  | *SEC24A* | ↓ in two datasets |
| ***SLC25A12*** | ↑ in two datasets |  |  | *PTGDR2* | ↓ in two datasets |
| ***FGF1*** | ↑ in two datasets |  |  | *MSRB1* | ↓ in two datasets |
| ***MAPK7*** | ↑ in two datasets |  |  | *PHF10* | ↓ in two datasets |
| ***PLEKHA2*** | ↑ in two datasets |  |  | *C8A* | ↓ in two datasets |
| ***FLJ32255*** | ↑ in two datasets |  |  | *SLC6A11* | ↓ in two datasets |
| ***TCF21*** | ↑ in two datasets |  |  | *NEU4* | ↓ in two datasets |
| ***AKAP12*** | ↑ in two datasets |  |  | *H2AFY2* | ↓ in two datasets |
| ***PAPPA*** | ↑ in two datasets |  |  | *FTCD* | ↓ in two datasets |
| ***SUSD5*** | ↑ in two datasets |  |  | *CLPX* | ↓ in two datasets |
| ***HBEGF*** | ↑ in two datasets |  |  | *SERINC2* | ↓ in two datasets |
| ***FLJ45513*** | ↑ in two datasets |  |  | *BRD3* | ↓ in two datasets |
| ***ZNF488*** | ↑ in two datasets |  |  | *NINL* | ↓ in two datasets |
| ***MARVELD1*** | ↑ in two datasets |  |  | *CCNT2* | ↓ in two datasets |
| ***TIMP2*** | ↑ in two datasets |  |  | *TBC1D24* | ↓ in two datasets |
| ***CUEDC1*** | ↑ in two datasets |  |  | *ICAM3* | ↓ in two datasets |
| ***C16orf45*** | ↑ in two datasets |  |  | *DCXR* | ↓ in two datasets |
| ***SNX19*** | ↑ in two datasets |  |  | *SPDYE2* | ↓ in two datasets |
| ***SRGAP1*** | ↑ in two datasets |  |  | *POLRMT* | ↓ in two datasets |
| ***ZDHHC2*** | ↑ in two datasets |  |  | *MMP15* | ↓ in two datasets |
| ***SPATA4*** | ↑ in two datasets |  |  | *SECISBP2* | ↓ in two datasets |
| ***LINC01139*** | ↑ in two datasets |  |  | *COX19* | ↓ in two datasets |
| ***LOC100506844*** | ↑ in two datasets |  |  | *RASL10B* | ↓ in two datasets |
| ***TRIM2*** | ↑ in two datasets |  |  | *LDLRAD4* | ↓ in two datasets |
| ***NEGR1*** | ↑ in two datasets |  |  | *MVB12B* | ↓ in two datasets |
| ***FAR2*** | ↑ in two datasets |  |  | *CYB5A* | ↓ in two datasets |
| ***GLIS2*** | ↑ in two datasets |  |  | *COG2* | ↓ in two datasets |
| ***WWTR1*** | ↑ in two datasets |  |  | *LARGE2* | ↓ in two datasets |
| ***NRIP3*** | ↑ in two datasets |  |  | *KDELR3* | ↓ in two datasets |
| ***LRP12*** | ↑ in two datasets |  |  | *CA14* | ↓ in two datasets |
| ***SLC36A4*** | ↑ in two datasets |  |  | *ATP13A1* | ↓ in two datasets |
| ***S100A10*** | ↑ in two datasets |  |  | *PRPF40A* | ↓ in two datasets |
| ***GJC1*** | ↑ in two datasets |  |  | *C14orf1* | ↓ in two datasets |
| ***MROH1*** | ↑ in two datasets |  |  | *GNG7* | ↓ in two datasets |
| ***BLVRB*** | ↑ in two datasets |  |  | *ZDHHC14* | ↓ in two datasets |
| ***LFNG*** | ↑ in two datasets |  |  | *TEF* | ↓ in two datasets |
| ***IGFBP6*** | ↑ in two datasets |  |  | *OCEL1* | ↓ in two datasets |
| ***NENF*** | ↑ in two datasets |  |  | *FAM60A* | ↓ in two datasets |
| ***QSOX1*** | ↑ in two datasets |  |  | *DCAF11* | ↓ in two datasets |
| ***NGF*** | ↑ in two datasets |  |  | *ISM1* | ↓ in two datasets |
| ***TFAP2C*** | ↑ in two datasets |  |  | *INTS10* | ↓ in two datasets |
| ***PCDHB8*** | ↑ in two datasets |  |  | *TM6SF2* | ↓ in two datasets |
| ***B4GALT5*** | ↑ in two datasets |  |  | *GATA2-AS1* | ↓ in two datasets |
| ***ACYP1*** | ↑ in two datasets |  |  | *ESF1* | ↓ in two datasets |
| ***FBXO16*** | ↑ in two datasets |  |  | *RRBP1* | ↓ in two datasets |
| ***DPYSL2*** | ↑ in two datasets |  |  | *PTPN18* | ↓ in two datasets |
| ***PEAR1*** | ↑ in two datasets |  |  | *LIMK2* | ↓ in two datasets |
| ***HOXD-AS2*** | ↑ in two datasets |  |  | *ZNF101* | ↓ in two datasets |
| ***SPDL1*** | ↑ in two datasets |  |  | *ABCA5* | ↓ in two datasets |
| ***TIGD7*** | ↑ in two datasets |  |  | *ZNF77* | ↓ in two datasets |
| ***CCND3*** | ↑ in two datasets |  |  | *SOX13* | ↓ in two datasets |
| ***SH3BGRL3*** | ↑ in two datasets |  |  | *EMC3* | ↓ in two datasets |
| ***SPSB2*** | ↑ in two datasets |  |  | *CBX4* | ↓ in two datasets |
| ***KIF3C*** | ↑ in two datasets |  |  | *ZUFSP* | ↓ in two datasets |
| ***TPGS2*** | ↑ in two datasets |  |  | *FNBP1* | ↓ in two datasets |
| ***PLEKHA3*** | ↑ in two datasets |  |  | *ADH5* | ↓ in two datasets |
| ***NEIL3*** | ↑ in two datasets |  |  | *PYM1* | ↓ in two datasets |
| ***S100A3*** | ↑ in two datasets |  |  | *CHCHD10* | ↓ in two datasets |
| ***NUDT11*** | ↑ in two datasets |  |  | *DOT1L* | ↓ in two datasets |
| ***COL5A1*** | ↑ in two datasets |  |  | *ZNF385B* | ↓ in two datasets |
| ***DISP2*** | ↑ in two datasets |  |  | *CFHR2* | ↓ in two datasets |
| ***CTPS1*** | ↑ in two datasets |  |  | *RFXANK* | ↓ in two datasets |
| ***ZBED5-AS1*** | ↑ in two datasets |  |  | *TOMM70* | ↓ in two datasets |
| ***SLC9A6*** | ↑ in two datasets |  |  | *RNF208* | ↓ in two datasets |
| ***NCEH1*** | ↑ in two datasets |  |  | *RAB1A* | ↓ in two datasets |
| ***LOC643085*** | ↑ in two datasets |  |  | *CUX1* | ↓ in two datasets |
| ***ARHGAP10*** | ↑ in two datasets |  |  | *MANBA* | ↓ in two datasets |
| ***ATP6V1E2*** | ↑ in two datasets |  |  | *FGD3* | ↓ in two datasets |
| ***CENPI*** | ↑ in two datasets |  |  | *SLC26A6* | ↓ in two datasets |
| ***TMEM161B-AS1*** | ↑ in two datasets |  |  | *ZNF563* | ↓ in two datasets |
| ***DEPDC1*** | ↑ in two datasets |  |  | *SCLY* | ↓ in two datasets |
| ***PAX2*** | ↑ in two datasets |  |  | *LSM3* | ↓ in two datasets |
| ***ADAMTS15*** | ↑ in two datasets |  |  | *ARMT1* | ↓ in two datasets |
| ***C9orf84*** | ↑ in two datasets |  |  | *JHDM1D-AS1* | ↓ in two datasets |
| ***ZBTB18*** | ↑ in two datasets |  |  | *ZNF710* | ↓ in two datasets |
| ***HMGN4*** | ↑ in two datasets |  |  | *THUMPD3-AS1* | ↓ in two datasets |
| ***MGEA5*** | ↑ in two datasets |  |  | *CDK19* | ↓ in two datasets |
| ***CD58*** | ↑ in two datasets |  |  | *LOC101928303* | ↓ in two datasets |
| ***AP1S3*** | ↑ in two datasets |  |  | *SMA4* | ↓ in two datasets |
| ***TCF19*** | ↑ in two datasets |  |  | *CAPN3* | ↓ in two datasets |
| ***APLP1*** | ↑ in two datasets |  |  | *PPHLN1* | ↓ in two datasets |
| ***CFLAR-AS1*** | ↑ in two datasets |  |  | *FBP1* | ↓ in two datasets |
| ***HRK*** | ↑ in two datasets |  |  | *YIPF2* | ↓ in two datasets |
| ***TMEM234*** | ↑ in two datasets |  |  | *EFNA4* | ↓ in two datasets |
| ***JUN*** | ↑ in two datasets |  |  | *STXBP4* | ↓ in two datasets |
| ***SH2D5*** | ↑ in two datasets |  |  | *BRD4* | ↓ in two datasets |
| ***UTS2*** | ↑ in two datasets |  |  | *PIGL* | ↓ in two datasets |
| ***RGS4*** | ↑ in two datasets |  |  | *KANSL1L* | ↓ in two datasets |
| ***IRF2BPL*** | ↑ in two datasets |  |  | *TTC33* | ↓ in two datasets |
| ***GRAMD3*** | ↑ in two datasets |  |  | *ADGRA3* | ↓ in two datasets |
| ***WDR13*** | ↑ in two datasets |  |  | *GATC* | ↓ in two datasets |
| ***DDX58*** | ↑ in two datasets |  |  | *ANKRD13A* | ↓ in two datasets |
| ***DAGLB*** | ↑ in two datasets |  |  | *CDK5RAP2* | ↓ in two datasets |
| ***LINC01278*** | ↑ in two datasets |  |  | *ARID3B* | ↓ in two datasets |
| ***PCDHB15*** | ↑ in two datasets |  |  | *PLEKHA6* | ↓ in two datasets |
| ***ACBD5*** | ↑ in two datasets |  |  | *FDFT1* | ↓ in two datasets |
| ***CC2D1B*** | ↑ in two datasets |  |  | *ZNF592* | ↓ in two datasets |
| ***WDR45B*** | ↑ in two datasets |  |  | *ITCH* | ↓ in two datasets |
| ***MMGT1*** | ↑ in two datasets |  |  | *PRKAR1B* | ↓ in two datasets |
| ***UPF3B*** | ↑ in two datasets |  |  | *PCBP2* | ↓ in two datasets |
| ***IRX5*** | ↑ in two datasets |  |  | *COLCA1* | ↓ in two datasets |
| ***ZXDB///ZXDA*** | ↑ in two datasets |  |  | *ARL4D* | ↓ in two datasets |
| ***SERTAD1*** | ↑ in two datasets |  |  | *SWSAP1* | ↓ in two datasets |
| ***CDC42BPB*** | ↑ in two datasets |  |  | *C19orf43* | ↓ in two datasets |
| ***PBX3*** | ↑ in two datasets |  |  | *AIFM3* | ↓ in two datasets |
| ***PLCL2*** | ↑ in two datasets |  |  | *SPOUT1* | ↓ in two datasets |
| ***STX1A*** | ↑ in two datasets |  |  | *GTF2A2* | ↓ in two datasets |
| ***LOC153684*** | ↑ in two datasets |  |  | *GATS* | ↓ in two datasets |
| ***DUT*** | ↑ in two datasets |  |  | *ABHD3* | ↓ in two datasets |
| ***PMVK*** | ↑ in two datasets |  |  | *FBXO9* | ↓ in two datasets |
| ***MRPL15*** | ↑ in two datasets |  |  | *CHD6* | ↓ in two datasets |
| ***C8orf37*** | ↑ in two datasets |  |  | *APOC2* | ↓ in two datasets |
| ***LOC646762*** | ↑ in two datasets |  |  | *GMEB2* | ↓ in two datasets |
| ***SLC5A3*** | ↑ in two datasets |  |  | *RAMP2* | ↓ in two datasets |
| ***CMC4///MTCP1*** | ↑ in two datasets |  |  | *NUDT16L1* | ↓ in two datasets |
| ***REEP5*** | ↑ in two datasets |  |  | *MED23* | ↓ in two datasets |
| ***SEC61A2*** | ↑ in two datasets |  |  | *IGF2BP1* | ↓ in two datasets |
| ***LAMA2*** | ↑ in two datasets |  |  | *ILVBL* | ↓ in two datasets |
| ***INPPL1*** | ↑ in two datasets |  |  | *TIMP4* | ↓ in two datasets |
| ***CORO2B*** | ↑ in two datasets |  |  | *TXNDC11* | ↓ in two datasets |
| ***REXO2*** | ↑ in two datasets |  |  | *NEK8* | ↓ in two datasets |
| ***STRA13*** | ↑ in two datasets |  |  | *SMDT1* | ↓ in two datasets |
| ***ACER3*** | ↑ in two datasets |  |  | *MPV17L2* | ↓ in two datasets |
| ***SNX7*** | ↑ in two datasets |  |  | *FAM120B* | ↓ in two datasets |
| ***ATP2B4*** | ↑ in two datasets |  |  | *NPM2* | ↓ in two datasets |
| ***LINC00173*** | ↑ in two datasets |  |  | *SREBF2* | ↓ in two datasets |
| ***PITRM1*** | ↑ in two datasets |  |  | *LINC00479* | ↓ in two datasets |
| ***ZNF615*** | ↑ in two datasets |  |  | *EXOSC3* | ↓ in two datasets |
| ***STMN1*** | ↑ in two datasets |  |  | *ALG14* | ↓ in two datasets |
| ***MFAP3L*** | ↑ in two datasets |  |  | *HSD17B7* | ↓ in two datasets |
| ***NUDT1*** | ↑ in two datasets |  |  | *RPS28* | ↓ in two datasets |
| ***GPSM2*** | ↑ in two datasets |  |  | *PLIN5* | ↓ in two datasets |
| ***TK1*** | ↑ in two datasets |  |  | *TOMM5* | ↓ in two datasets |
| ***EOGT*** | ↑ in two datasets |  |  | *MBD4* | ↓ in two datasets |
| ***CSTF2*** | ↑ in two datasets |  |  | *PRDX6* | ↓ in two datasets |
| ***RNF214*** | ↑ in two datasets |  |  | *ARL5B* | ↓ in two datasets |
| ***TNFRSF12A*** | ↑ in two datasets |  |  | *TBC1D22A* | ↓ in two datasets |
| ***LRP10*** | ↑ in two datasets |  |  | *SLC26A1* | ↓ in two datasets |
| ***LOH12CR2*** | ↑ in two datasets |  |  | *TAMM41* | ↓ in two datasets |
| ***ZDHHC16*** | ↑ in two datasets |  |  | *SLC11A2* | ↓ in two datasets |
| ***PGK1*** | ↑ in two datasets |  |  | *RBM6* | ↓ in two datasets |
| ***PPP3CC*** | ↑ in two datasets |  |  | *OS9* | ↓ in two datasets |
| ***TMEM237*** | ↑ in two datasets |  |  | *TFB1M* | ↓ in two datasets |
| ***POLD3*** | ↑ in two datasets |  |  | *NES* | ↓ in two datasets |
| ***TFAP2A-AS1*** | ↑ in two datasets |  |  | *RNF207* | ↓ in two datasets |
| ***IER5*** | ↑ in two datasets |  |  | *SCAP* | ↓ in two datasets |
| ***CAP2*** | ↑ in two datasets |  |  | *TRABD2B* | ↓ in two datasets |
| ***CDC25B*** | ↑ in two datasets |  |  | *HACD3* | ↓ in two datasets |
| ***JADE2*** | ↑ in two datasets |  |  | *VTI1A* | ↓ in two datasets |
| ***TRIM45*** | ↑ in two datasets |  |  | *H6PD* | ↓ in two datasets |
| ***CUL2*** | ↑ in two datasets |  |  | *KYAT1* | ↓ in two datasets |
| ***HAUS7///TREX2*** | ↑ in two datasets |  |  | *MESDC2* | ↓ in two datasets |
| ***TMOD2*** | ↑ in two datasets |  |  | *PLAGL2* | ↓ in two datasets |
| ***TRPC4*** | ↑ in two datasets |  |  | *RCN2* | ↓ in two datasets |
| ***CLCF1*** | ↑ in two datasets |  |  | *KRTDAP* | ↓ in two datasets |
| ***SLC35E4*** | ↑ in two datasets |  |  | *DCUN1D3* | ↓ in two datasets |
| ***UPF2*** | ↑ in two datasets |  |  | *CAPN12* | ↓ in two datasets |
| ***LPP-AS2*** | ↑ in two datasets |  |  | *OAZ1* | ↓ in two datasets |
| ***FOXC2*** | ↑ in two datasets |  |  | *KDELR2* | ↓ in two datasets |
| ***METTL23*** | ↑ in two datasets |  |  | *NMT1* | ↓ in two datasets |
| ***TRNAU1AP*** | ↑ in two datasets |  |  | *CCNC* | ↓ in two datasets |
| ***CECR7*** | ↑ in two datasets |  |  | *PSMB1* | ↓ in two datasets |
| ***VAT1*** | ↑ in two datasets |  |  | *BAIAP2L2* | ↓ in two datasets |
| ***ABCB7*** | ↑ in two datasets |  |  | *LOC100505912* | ↓ in two datasets |
| ***ANXA2P2*** | ↑ in two datasets |  |  | *EIF5* | ↓ in two datasets |
| ***CCHCR1*** | ↑ in two datasets |  |  | *ACOT12* | ↓ in two datasets |
| ***ZNF226*** | ↑ in two datasets |  |  | *PKP4* | ↓ in two datasets |
| ***RAB18*** | ↑ in two datasets |  |  | *CARMIL2* | ↓ in two datasets |
| ***PCDHB13*** | ↑ in two datasets |  |  | *XKR8* | ↓ in two datasets |
| ***NPHP3*** | ↑ in two datasets |  |  | *AMD1* | ↓ in two datasets |
| ***MESP1*** | ↑ in two datasets |  |  | *PTPRCAP* | ↓ in two datasets |
| ***EFR3B*** | ↑ in two datasets |  |  | *VPS25* | ↓ in two datasets |
| ***KCNK6*** | ↑ in two datasets |  |  | *MAF* | ↓ in two datasets |
| ***LINC00592*** | ↑ in two datasets |  |  | *RMDN3* | ↓ in two datasets |
| ***AGAP2-AS1*** | ↑ in two datasets |  |  | *BCOR* | ↓ in two datasets |
| ***PFDN1*** | ↑ in two datasets |  |  | *AMACR* | ↓ in two datasets |
| ***ATP13A2*** | ↑ in two datasets |  |  |  |  |
| ***ADRB1*** | ↑ in two datasets |  |  |  |  |
| ***LOC100268168*** | ↑ in two datasets |  |  |  |  |
| ***ANKRD26*** | ↑ in two datasets |  |  |  |  |
| ***ZNF570*** | ↑ in two datasets |  |  |  |  |
| ***DUSP18*** | ↑ in two datasets |  |  |  |  |
| ***MUS81*** | ↑ in two datasets |  |  |  |  |
| ***BHLHE41*** | ↑ in two datasets |  |  |  |  |
| ***PADI1*** | ↑ in two datasets |  |  |  |  |
| ***BIRC5*** | ↑ in two datasets |  |  |  |  |
| ***CTSL*** | ↑ in two datasets |  |  |  |  |
| ***KLF6*** | ↑ in two datasets |  |  |  |  |
| ***SEC14L1*** | ↑ in two datasets |  |  |  |  |
| ***MEA1*** | ↑ in two datasets |  |  |  |  |
| ***ATXN3*** | ↑ in two datasets |  |  |  |  |
| ***C10orf88*** | ↑ in two datasets |  |  |  |  |
| ***SUGT1P1*** | ↑ in two datasets |  |  |  |  |
| ***SYT16*** | ↑ in two datasets |  |  |  |  |
| ***TRMT2B*** | ↑ in two datasets |  |  |  |  |
| ***COL6A1*** | ↑ in two datasets |  |  |  |  |
| ***SPATA5*** | ↑ in two datasets |  |  |  |  |
| ***UBE2Z*** | ↑ in two datasets |  |  |  |  |
| ***TMSB10*** | ↑ in two datasets |  |  |  |  |
| ***NPAT*** | ↑ in two datasets |  |  |  |  |
| ***CENPB*** | ↑ in two datasets |  |  |  |  |
| ***TRMT1L*** | ↑ in two datasets |  |  |  |  |
| ***INO80E*** | ↑ in two datasets |  |  |  |  |
| ***PSMD10*** | ↑ in two datasets |  |  |  |  |
| ***LOC440028*** | ↑ in two datasets |  |  |  |  |
| ***ABI1*** | ↑ in two datasets |  |  |  |  |
| ***TAPBP*** | ↑ in two datasets |  |  |  |  |
| ***APPL1*** | ↑ in two datasets |  |  |  |  |
| ***DCLRE1B*** | ↑ in two datasets |  |  |  |  |
| ***TTC30B*** | ↑ in two datasets |  |  |  |  |
| ***ADPRHL2*** | ↑ in two datasets |  |  |  |  |
| ***MBNL1-AS1*** | ↑ in two datasets |  |  |  |  |
| ***SLC9A1*** | ↑ in two datasets |  |  |  |  |
| ***PSMC3IP*** | ↑ in two datasets |  |  |  |  |
| ***SAP30BP*** | ↑ in two datasets |  |  |  |  |
| ***CCDC24*** | ↑ in two datasets |  |  |  |  |
| ***fcFOXL1*** | ↑ in two datasets |  |  |  |  |
| ***H2AFJ*** | ↑ in two datasets |  |  |  |  |
| ***CCDC71L*** | ↑ in two datasets |  |  |  |  |
| ***RTN4*** | ↑ in two datasets |  |  |  |  |
| ***JMJD6*** | ↑ in two datasets |  |  |  |  |
| ***HDAC8*** | ↑ in two datasets |  |  |  |  |
| ***FAM188A*** | ↑ in two datasets |  |  |  |  |
| ***STAM*** | ↑ in two datasets |  |  |  |  |
| ***STUB1*** | ↑ in two datasets |  |  |  |  |
| ***XPNPEP1*** | ↑ in two datasets |  |  |  |  |
| ***NSD1*** | ↑ in two datasets |  |  |  |  |
| ***CENPM*** | ↑ in two datasets |  |  |  |  |
| ***MIR31HG*** | ↑ in two datasets |  |  |  |  |
| ***CSE1L*** | ↑ in two datasets |  |  |  |  |
| ***SZRD1*** | ↑ in two datasets |  |  |  |  |
| ***DCLRE1A*** | ↑ in two datasets |  |  |  |  |
| ***ZNF341*** | ↑ in two datasets |  |  |  |  |
| ***SLC2A12*** | ↑ in two datasets |  |  |  |  |
| ***TSPAN10*** | ↑ in two datasets |  |  |  |  |
| ***DDX27*** | ↑ in two datasets |  |  |  |  |
| ***C18orf25*** | ↑ in two datasets |  |  |  |  |
| ***PSMC5*** | ↑ in two datasets |  |  |  |  |
| ***WDR7*** | ↑ in two datasets |  |  |  |  |
| ***KPNA6*** | ↑ in two datasets |  |  |  |  |
| ***MKI67*** | ↑ in two datasets |  |  |  |  |
| ***PI4K2A*** | ↑ in two datasets |  |  |  |  |
| ***FICD*** | ↑ in two datasets |  |  |  |  |
| ***GPR137*** | ↑ in two datasets |  |  |  |  |
| ***OLR1*** | ↑ in two datasets |  |  |  |  |
| ***RSG1*** | ↑ in two datasets |  |  |  |  |
| ***CYP2E1*** | ↑ in two datasets |  |  |  |  |
| ***ZNF37BP*** | ↑ in two datasets |  |  |  |  |
| ***NCAPD3*** | ↑ in two datasets |  |  |  |  |
| ***POP4*** | ↑ in two datasets |  |  |  |  |
| ***ZNF397*** | ↑ in two datasets |  |  |  |  |
| ***TLR4*** | ↑ in two datasets |  |  |  |  |
| ***BTF3L4*** | ↑ in two datasets |  |  |  |  |
| ***XRCC3*** | ↑ in two datasets |  |  |  |  |
| ***ANXA7*** | ↑ in two datasets |  |  |  |  |
| ***EXOC5*** | ↑ in two datasets |  |  |  |  |
| ***BTN2A1*** | ↑ in two datasets |  |  |  |  |
| ***LOC284080*** | ↑ in two datasets |  |  |  |  |
| ***ZBTB45*** | ↑ in two datasets |  |  |  |  |
