# Supplementary material for: Liver cancer cell lines distinctly mimic the metabolic gene expression pattern of the corresponding human tumours
Source: J Exp Clin Cancer Res. 2018 Sep 3;37:211. doi: 10.1186/s13046-018-0872-6 (PMC6122702; doi:10.1186/s13046-018-0872-6)
Supplement: Supplementary file 3 — Table S2. HCC-associated genes. Contains a set of genes often associated with HCC, including those related to epithelial–mesenchymal transition. (DOCX 19 kb) [file 13046_2018_872_MOESM3_ESM.docx]

| **Table S2**. HCC-associated genes | |  |
| --- | --- | --- |
|  |  |  |
| **Genes** | **Name** |  |
| *APC* | APC, WNT signaling pathway regulator |  |
| *ARID1A* | AT-rich interaction domain 1A |  |
| *ARID1B* | AT-rich interaction domain 1B |  |
| *ATP7B* | ATPase copper transporting beta |  |
| *CUL3* | cullin 3 |  |
| *FRK* | fyn related Src family tyrosine kinase |  |
| *GLUL* | glutamate-ammonia ligase |  |
| *GNAS* | GNAS complex locus |  |
| *HNF1A* | HNF1 homeobox A |  |
| *HRAS* | HRas proto-oncogene, GTPase |  |
| *JAK1* | Janus kinase 1 |  |
| *KEAP1* | kelch like ECH associated protein 1 |  |
| *KIF1B* | kinesin family member 1B |  |
| *LGR5* | leucine rich repeat containing G protein-coupled receptor 5 |  |
| *MICA* | MHC class I polypeptide-related sequence A |  |
| *NFE2L2* | nuclear factor, erythroid 2 like 2 |  |
| *NRAS* | neuroblastoma RAS viral oncogene homolog |  |
| *ORAOV1* | oral cancer overexpressed 1 |  |
| *PGD* | phosphogluconate dehydrogenase |  |
| *PTEN* | phosphatase and tensin homolog |  |
| *RPS6KA3* | ribosomal protein S6 kinase A3 |  |
| *SERPINA1* | serpin family A member 1 |  |
| *STAT3* | signal transducer and activator of transcription 3 |  |
| *TGFB1* | Transforming Growth Factor Beta 1 |  |
| *TNFSF10* | tumor necrosis factor superfamily member 10 |  |
| *UBE4B* | ubiquitination factor E4B |  |
| *UROD* | uroporphyrinogen decarboxylase |  |
| *ARID2* | AT-rich interaction domain 2 |  |
| *ATM* | ATM serine/threonine kinase |  |
| *AXIN1* | axin 1 |  |
| *BRD7* | bromodomain containing 7 |  |
| *CCNA1* | cyclin A1 |  |
| *CCNA2* | cyclin A2 |  |
| *CCND1* | cyclin D1 |  |
| *CCNE1* | cyclin E1 |  |
| *CDKN2A* | cyclin dependent kinase inhibitor 2A |  |
| *CTNNB1* | catenin beta 1 |  |
| *DEPDC5* | DEP domain containing 5 |  |
| *EGF* | epidermal growth factor |  |
| *FAH* | fumarylacetoacetate hydrolase |  |
| *FGF19* | fibroblast growth factor 19 |  |
| *FGF4* | fibroblast growth factor 4 |  |
| *G6PC* | glucose-6-phosphatase catalytic subunit |  |
| *GSK3B* | glycogen synthase kinase 3 beta |  |
| *GSTT1* | glutathione S-transferase theta 1 |  |
| *GSTT2* | glutathione S-transferase theta 2 (gene/pseudogene) |  |
| *HFE* | hemochromatosis type 1 |  |
| *HMBS* | hydroxymethylbilane synthase |  |
| *IL10* | interleukin 10 |  |
| *IL1B* | interleukin 1 beta |  |
| *IL6ST* | interleukin 6 signal transducer |  |
| *IRF2* | interferon regulatory factor 2 |  |
| *KRAS* | KRAS proto-oncogene, GTPase |  |
| *MDM2* | MDM2 proto-oncogene |  |
| *MLL* | myeloid/lymphoid or mixed-lineage leukemia 1 |  |
| *MLL2* | myeloid/lymphoid or mixed-lineage leukemia 2 |  |
| *KMT2B* | Lysine Methyltransferase 2B (also myeloid/lymphoid or mixed-lineage leukemia 4) |  |
| *MPO* | myeloperoxidase |  |
| *PIK3CA* | phosphatidylinositol-4,5-bisphosphate 3-kinase catalytic subunit alpha |  |
| *PNPLA3* | patatin like phospholipase domain containing 3 |  |
| *RB1* | RB transcriptional corepressor 1 |  |
| *SOD2* | superoxide dismutase 2, mitochondrial |  |
| *STAT4* | signal transducer and activator of transcription 4 |  |
| *TERT* | telomerase reverse transcriptase |  |
| *TNF* | Tumor Necrosis Factor |  |
| *TP53* | tumor protein p53 |  |
| *TPTE2* | transmembrane phosphoinositide 3-phosphatase and tensin homolog 2 |  |
| *TSC1* | tuberous sclerosis 1 |  |
| *TSC2* | tuberous sclerosis 2 |  |
| *CDH1* | cadherin 1 | EMT related genes |
| *CDH2* | cadherin 2 | EMT related genes |
| *ECM1* | extracellular matrix protein 1 | EMT related genes |
| *MMP2* | matrix metallopeptidase 2 | EMT related genes |
| *MMP9* | matrix metallopeptidase 9 | EMT related genes |
| *TWIST1* | twist family bHLH transcription factor 1 | EMT related genes |
| *VIM* | vimentin | EMT related genes |
| *ZEB2* | zinc finger E-box binding homeobox 2 | EMT related genes |
| *ECM2* | extracellular matrix protein 2 | EMT related genes |
| *SNAI1* | snail family transcriptional repressor 1 | EMT related genes |
| *SNAI2* | snail family transcriptional repressor 2 | EMT related genes |
| *TWIST2* | twist family bHLH transcription factor 2 | EMT related genes |
| *ZEB1* | zinc finger E-box binding homeobox 1 | EMT related genes |
|  |  |  |
| Most of these genes were compiled from published review: https://www.ncbi.nlm.nih.gov/pubmed/26099527 | | |
