# Supplementary material for: Liver cancer cell lines distinctly mimic the metabolic gene expression pattern of the corresponding human tumours
Source: J Exp Clin Cancer Res. 2018 Sep 3;37:211. doi: 10.1186/s13046-018-0872-6 (PMC6122702; doi:10.1186/s13046-018-0872-6)
Supplement: Supplementary file 4 — Table S3. Top pathway enrichment and the specific genes involved. Enrichment analysis was performed with the genes more (3a) or (3b) lowly expressed in the poorly differentiated relative to well-differentiated HCC cell lines. (DOCX 18 kb) [file 13046_2018_872_MOESM4_ESM.docx]

| **Table S3. Top pathway enrichment and the specific genes involved. Generated using genes more (3a) or (3b) lowly expressed in the poorly differentiated relative to well differentiated HCC cell lines**  **3a.** KEGG pathway enrichment of upregulated/highly expressed genes in poorly differentiated (HLF, HLE, SNU-449) compared to well-differentiated HCC cell lines (HEP3B, HUH7 and HEPG2) | | |
| --- | --- | --- |
|  |  |  |
| Pathway | Number of genes | Genes involved |
| **hsa05200:Pathways in cancer** | 59 | *GNA13, ADCY3, FGF5, HRAS, MITF, GLI2, MMP1, TGFB2, FLT3LG, CUL2, CDC42, PLCB3, PLCB4, PRKACB, FGF1, FGF2, AKT3, CSF2RA, PRKCA, CTBP2, RELA, CTNNA1, ARHGEF12, CCDC6, JUN, GNB5, WNT5A, FGFR1, WNT5B, GNAI2, XIAP, PML, GNG11, GNG12, ITGB1, BCL2, TRAF5, IL6, COL4A1, MET, CBL, SMAD3, ITGA3, SMAD2, BIRC5, FZD2, BAD, MAPK10, BIRC3, APPL1, BIRC2, FZD6, WNT2B, LAMA2, LAMA3, ARAF, TCEB2, JAK1, IKBKB* |
| hsa04010:MAPK signaling pathway | 43 | *FGFR1, FGF5, HRAS, ZAK, CACNB1, ELK1, CACNB3, GNG12, TGFB2, TNFRSF1A, MAP3K6, CDC42, BDNF, MAP3K5, ELK4, PPP3CB, PPP3CC, RRAS, PRKACB, FGF1, FGF2, RASA1, AKT3, PRKCA, RELA, NF1, MAPK11, MAPK10, FLNA, STK3, CDC25B, RPS6KA5, RPS6KA4, DUSP1, JUN, RRAS2, STMN1, MAPK7, MAP3K14, IKBKB, MAP3K12, DUSP7, NGF* |
| hsa05205:Proteoglycans in cancer | 40 | *WNT5A, CAV2, FGFR1, CAV1, HRAS, WNT5B, CAMK2G, ELK1, TLR4, ITGB3, ITGB1, PXN, IQGAP1, TGFB2, CDC42, ANK1, CD44, HPSE, RRAS, PRKACB, MSN, FGF2, AKT3, TWIST1, PRKCA, MET, CBL, MAPK11, FZD2, ARHGEF12, FLNA, PLAUR, FZD6, WNT2B, CTSL, RRAS2, ARAF, HBEGF, PLAU, SLC9A1* |
| hsa05166:HTLV-I infection | 38 | *ADCY3, WNT5A, TSPO, HRAS, WNT5B, XIAP, ELK1, IL15, MYBL1, TGFB2, TNFRSF1A, CDKN2C, ELK4, PPP3CB, PPP3CC, RRAS, PRKACB, FOSL1, AKT3, IL6, KAT2B, LTBR, RELA, SMAD3, SMAD2, CD40, FZD2, ATM, WNT2B, FZD6, POLD3, CCND3, ETS1, JUN, RRAS2, JAK1, MAP3K14, IKBKB* |
| hsa04510:Focal adhesion | 37 | *CAV2, CAV1, HRAS, XIAP, TNC, BCAR1, ELK1, ITGB3, ITGB1, PXN, MYL9, VCL, CDC42, DOCK1, BCL2, COL6A2, COL6A1, AKT3, PRKCA, COL4A1, MET, ACTN1, ITGA3, MYL12A, MAPK10, BAD, CAPN2, BIRC3, BIRC2, COL5A1, FLNA, LAMA2, LAMA3, CCND3, JUN, MYLK, PARVA* |
| hsa04810:Regulation of actin cytoskeleton | 33 | *GNA13, FGFR1, FGF5, HRAS, SSH1, BCAR1, WASF2, GNG12, ITGB3, ITGB1, PXN, IQGAP1, MYL9, VCL, CDC42, DOCK1, PFN4, RRAS, MSN, FGF1, FGF2, GIT1, ACTN1, ITGA3, MYL12A, ARHGEF12, RRAS2, CFL1, ARAF, TMSB4X, MYLK, SLC9A1, PIP4K2B* |
| hsa04390:Hippo signaling pathway | 27 | *WNT5A, YWHAZ, WNT5B, BMPR2, GLI2, TGFB2, FRMD6, SERPINE1, FGF1, FBXW11, PARD6B, NF2, MPP5, YWHAB, SMAD3, TEAD1, SMAD2, BIRC5, FZD2, WWTR1, CTNNA1, STK3, WNT2B, FZD6, CCND3, YWHAQ, BMP5* |
| hsa04380:Osteoclast differentiation | 24 | *FOSL2, SOCS3, RELA, MITF, FHL2, MAPK11, MAPK10, FOSB, ITGB3, SIRPA, TGFB2, IRF9, TNFRSF1A, CYLD, CAMK4, SQSTM1, JUN, PPP3CB, PPP3CC, JAK1, IKBKB, MAP3K14, FOSL1, AKT3* |
| hsa04668:TNF signaling pathway | 20 | *IL6, CCL2, SOCS3, RELA, MAPK11, MAPK10, IL15, BIRC3, BIRC2, RPS6KA5, LIF, TNFRSF1A, MAP3K5, RPS6KA4, JUN, MLKL, IKBKB, MAP3K14, TRAF5, AKT3* |
| hsa04064:NF-kappa B signaling pathway | 20 | *IRAK1, BCL10, LTBR, XIAP, RELA, TIRAP, TRIM25, TLR4, CD40, BIRC3, BIRC2, ATM, DDX58, TNFRSF1A, BCL2, ERC1, IKBKB, MAP3K14, TRAF5, PLAU* |

| **3b.** KEGG pathway enrichment of downregulated/lowly expressed genes in poorly differentiated (HLF, HLE, SNU-449) compared to well-differentiated HCC cell lines (HEP3B, HUH7 and HEPG2) | | |
| --- | --- | --- |
|  |  |  |
| Pathway | Number of genes | Genes involved |
| **hsa01100:Metabolic pathways** | 159 | *ALAD, BTD, EHHADH, COX5A, ACSS3, UXS1, AGXT, ITPKA, FDFT1, FAH, GLDC, AGPS, SCLY, ST3GAL6, PHOSPHO2, RGN, ALDH6A1, PLD1, CYP1A1, FAXDC2, SPTLC3, FBP1, HAL, MOGS, PNPLA3, GLUL, BHMT, ABAT, PCCB, MDH2, MPST, ACAA1, GATC, CHKA, ACADSB, ASS1, NAGS, ENPP1, GLUD2, ALDOC, GLUD1, ENPP3, HMGCS1, CERS4, PAH, AGMAT, ASL, ACAT2, ARG1, ISYNA1, IDH2, IDH1, ALDH4A1, LIAS, AMD1, GCDH, NADK2, MGAT4A, DNMT3A, ST6GAL1, B4GAT1, UPB1, MAOA, SPHK1, MAOB, GALT, EPHX2, FDPS, HGD, CPS1, TST, ALDH2, QPRT, GK, DCXR, PC, ALG14, ACOX2, TM7SF2, ETNPPL, ACOX1, SEPHS2, CYP2J2, AMT, ANPEP, PSPH, CKB, PIGL, MCEE, MCCC1, PLCB1, PCYT2, SARDH, DHCR24, HPD, HYAL1, DDC, ACO2, CYCS, NDUFC2, NDUFA13, POLR1B, MAN1A1, LPIN2, CDS1, NDUFA11, PIGN, ATP6V1A, MTMR14, UMPS, PANK1, H6PD, PRDX6, TGDS, PKLR, AKR1D1, GPAM, UGP2, XYLB, NDUFB5, POLR2E, SORD, HSD17B2, ADPGK, HSD3B7, ADH5, ADH6, PLPP3, ALDH3A2, GCH1, ADH4, PLA2G12B, HAAO, ENO3, KYAT1, UGT2A3, HSD17B4, ACSL4, ACSL3, GALNT13, HSD17B7, MOGAT3, BCKDHA, CHDH, SHMT2, AMACR, NDUFA7, FTCD, KHK, ADI1, GBA2, AMDHD1, SDHC, UGT2B4, GAMT, PSAT1, LIPC, SCP2, CBS* |
| hsa01130:Biosynthesis of antibiotics | 42 | *TM7SF2, ASS1, ADPGK, AMT, EHHADH, ALDOC, ADH5, HMGCS1, ASL, PSPH, ACAT2, AGXT, ALDH3A2, FDFT1, GLDC, ARG1, ISYNA1, FNTA, IDH2, RGN, IDH1, ENO3, HSD17B7, BCKDHA, GCDH, SHMT2, ACO2, FAXDC2, FDPS, FBP1, AK3, PGM3, SDHC, TGDS, PKLR, ALDH2, PSAT1, PCCB, MDH2, UGP2, CBS, ACAA1* |
| hsa04610:Complement and coagulation cascades | 28 | *MBL2, A2M, C3, C5, F13B, FGG, FGA, SERPINA5, CD46, SERPINA1, C2, CFI, KNG1, F12, F10, CFB, C4BPB, F7, C4BPA, PROC, C8A, C8B, F5, SERPINF2, F2, SERPIND1, CPB2, PROS1* |
| hsa01200:Carbon metabolism | 28 | *GLUD2, ADPGK, EHHADH, ALDOC, GLUD1, AMT, ADH5, ACAT2, PSPH, AGXT, GLDC, MCEE, IDH2, ENO3, RGN, IDH1, ALDH6A1, SHMT2, ACO2, FBP1, CPS1, H6PD, SDHC, PKLR, PSAT1, PCCB, MDH2, PC* |
| hsa03320:PPAR signaling pathway | 21 | *ACOX2, PPARA, ACOX1, CPT2, EHHADH, RXRA, DBI, APOA2, APOA1, CD36, SORBS1, APOC3, FABP1, GK, ACSL4, ACSL3, SCP2, SLC27A2, PLTP, ACAA1, NR1H3* |
| hsa04146:Peroxisome | 21 | *ACOX2, ACOX1, EHHADH, AMACR, DECR2, EPHX2, AGXT, PECR, AGPS, MPV17L2, MPV17L, IDH2, IDH1, ABCD3, PEX13, HSD17B4, ACSL4, ACSL3, SCP2, SLC27A2, ACAA1* |
| hsa01230:Biosynthesis of amino acids | 18 | *SHMT2, ASS1, NAGS, ACO2, ALDOC, PAH, CPS1, ASL, PSPH, ARG1, GLUL, PKLR, IDH2, ENO3, IDH1, PSAT1, CBS, PC* |
| hsa00071:Fatty acid degradation | 14 | *GCDH, ACOX1, ACADSB, CPT2, EHHADH, ADH5, ADH6, ACAT2, ALDH3A2, ADH4, ALDH2, ACSL4, ACSL3, ACAA1* |
| hsa00260:Glycine, serine and threonine metabolism | 13 | *CHDH, SHMT2, AMT, MAOA, MAOB, PSPH, AGXT, GLDC, BHMT, GAMT, PSAT1, SARDH, CBS* |
| hsa04950:Maturity onset diabetes of the young | 11 | *HHEX, HNF1A, FOXA2, HNF4A, ONECUT1, FOXA3, BHLHA15, RFX6, SLC2A2, PKLR, HNF4G* |
