# Supplementary material for: Liver cancer cell lines distinctly mimic the metabolic gene expression pattern of the corresponding human tumours
Source: J Exp Clin Cancer Res. 2018 Sep 3;37:211. doi: 10.1186/s13046-018-0872-6 (PMC6122702; doi:10.1186/s13046-018-0872-6)
Supplement: Supplementary file 6 — Table S5. Complete list of downregulated HMGs concordantly low in the p.d. cells (part of Fig. 2c). (DOCX 14 kb) [file 13046_2018_872_MOESM6_ESM.docx]

**Table S5.** Complete list of downregulated HMGs concordantly low in the p.d. cells (part of Fig. 2c)

| *A1CF* |
| --- |
| *ABAT* |
| *ABCC6* |
| *ABCD3* |
| *ABHD10* |
| *ACAA1* |
| *ACADSB* |
| *ACOX1* |
| *ACOX2* |
| *ADH6* |
| *ADI1* |
| *AGXT* |
| *AKR1D1* |
| *ALDH2* |
| *ALDH4A1* |
| *ALDH6A1* |
| *ALDH8A1* |
| *AQP3* |
| *ARG1* |
| *ASL* |
| *ASS1* |
| *BCKDHA* |
| *BHMT* |
| *BHMT2* |
| *CBS* |
| *CP* |
| *CPS1* |
| *CPT2* |
| *CYB5A* |
| *CYP1A1* |
| *CYP2J2* |
| *DCXR* |
| *EHHADH* |
| *ENO3* |
| *ENPP1* |
| *EPHX2* |
| *FAH* |
| *FBP1* |
| *FTCD* |
| *FXN* |
| *GAMT* |
| *GCDH* |
| *GCH1* |
| *GK* |
| *GLDC* |
| *GLUD1* |
| *GYG2* |
| *HAAO* |
| *HAL* |
| *HGD* |
| *HNMT* |
| *HPD* |
| *HSD17B2* |
| *HYAL1* |
| *KHK* |
| *LIPC* |
| *LPIN2* |
| *MAOB* |
| *MGST2* |
| *MPC1* |
| *MTTP* |
| *NPC1L1* |
| *PAH* |
| *PC* |
| *PCCB* |
| *PECR* |
| *PIK3R1* |
| *PSAT1* |
| *QPRT* |
| *SARDH* |
| *SCP2* |
| *SLC16A10* |
| *SLC17A2* |
| *SLC25A15* |
| *SLC27A2* |
| *SLC2A2* |
| *SLC31A1* |
| *SLC37A4* |
| *SLC38A4* |
| *SLC39A14* |
| *SLC47A1* |
| *SLC7A2* |
| *SORD* |
| *ST3GAL6* |
| *ST6GAL1* |
| *SULT1A1* |
| *SULT1A2* |
| *SULT2A1* |
| *TF* |
| *TGDS* |
| *UGP2* |
| *UPB1* |
