# Supplementary material for: Liver cancer cell lines distinctly mimic the metabolic gene expression pattern of the corresponding human tumours
Source: J Exp Clin Cancer Res. 2018 Sep 3;37:211. doi: 10.1186/s13046-018-0872-6 (PMC6122702; doi:10.1186/s13046-018-0872-6)
Supplement: Supplementary file 8 — Table S7. Human HCC tissue-derived metabolic genes (HMGs) mimicked by HLE or HUH7 cells upon comparison. Contains HMGs whose expression pattern in tumours (high or low expression) is reflected either in HLE (poorly differentiated, in total 227 HMGs) or HUH7 cells (well-differentiated, 140 candidates), when the cells are compared. The included HMGs showed consistent expression pattern in the respective cell lines both in GSE57083 and GSE36133 (CCLE) datasets. Those that also showed similar pattern at the protein level are indicated. (DOCX 25 kb) [file 13046_2018_872_MOESM8_ESM.docx]

| **Table S7. Human HCC tissue-derived metabolic genes (HMGs) mimicked by HLE or HUH7 cells upon comparison** | |
| --- | --- |
| These HMGs differed in a consistent manner for the respective cell lines in both GSE57083 and GSE36133 (CCLE) dataset. In red are the HMGs that did not emerge in the cell line comparison (gene level) but were detected by proteomics analysis to be expressed as in human HCC microarrays. | |
|  |  |
| **Upregulated HMGs also high in HLE cells (gene and or protein)** | |
| ***MBOAT7*** | detected as high by proteomics |
| ***ATP6V0A1*** | detected as high by proteomics |
| ***GLS*** | detected as high by proteomics |
| ***BCAT2*** | detected as high by proteomics |
| ***SMS*** | detected as high by proteomics |
| ***SPTLC1*** | detected as high by proteomics |
| ***LTA4H*** | detected as high by proteomics |
| ***UQCRH*** | detected as high by proteomics |
| ***SLC25A3*** | detected as high by proteomics |
| ***GNS*** | detected as high by proteomics |
| ***PIGT*** | detected as high by proteomics |
| ***VDAC1*** | detected as high by proteomics |
| ***UQCRB*** | detected as high by proteomics |
| ***CYC1*** | detected as high by proteomics |
| ***ACOT7*** | detected as high by proteomics |
| ***CS*** | detected as high by proteomics |
| ***NPC1*** | detected as high by proteomics |
| ***VDAC3*** | detected as high by proteomics |
| ***PGK1*** | detected as high by proteomics |
| ***ENO1*** | detected as high by proteomics |
| ***G6PD*** | detected as high by proteomics |
| ***ATP5J2*** | detected as high by proteomics |
| ***COX6B1*** | detected as high by proteomics |
| ***IMPAD1*** | detected as high by proteomics |
| ***NDUFA8*** | detected as high by proteomics |
| ***ATP1B3*** | detected as high by proteomics |
| ***ATP5H*** | detected as high by proteomics |
| ***ATP1A1*** | detected as high by proteomics |
| ***DDOST*** | detected as high by proteomics |
| ***ME2*** | detected as high by proteomics |
| ***GMPS*** | detected as high by proteomics |
| *PFKP* | detected as high by proteomics |
| *NAT10* | detected as high by proteomics |
| *BLVRA* | detected as high by proteomics |
| *PYGB* | detected as high by proteomics |
| *SLC4A7* | detected as high by proteomics |
| *IMPDH2* | detected as high by proteomics |
| *CAD* | detected as high by proteomics |
| *TKT* | detected as high by proteomics |
| *HEXB* | detected as high by proteomics |
| *GNPDA1* | detected as high by proteomics |
| *TK1* | detected as high by proteomics |
| *FN3KRP* | detected as high by proteomics |
| *ADA* | detected as high by proteomics |
| *RRM2* |  |
| *SLC30A5* |  |
| *NDST1* |  |
| *SLC7A11* |  |
| *ATP6V1D* |  |
| *NDUFA4L2* |  |
| *RRM1* |  |
| *ELOVL5* |  |
| *GLTP* |  |
| *IDUA* |  |
| *ENPP4* |  |
| *PAICS* |  |
| *ALG6* |  |
| *COX8A* |  |
| *PGS1* |  |
| *ABCC4* |  |
| *NQO1* |  |
| *PTDSS2* |  |
| *HKDC1* |  |
| *NUDT2* |  |
| *CDS2* |  |
| *SLC29A1* |  |
| *DUT* |  |
| *ACYP1* |  |
| *SLC7A1* |  |
| *NUDT1* |  |
| *EXT2* |  |
| *SLC39A6* |  |
| *G6PC3* |  |
| *MFSD1* |  |
| *OAS3* |  |
| *PIGF* |  |
| *GCNT3* |  |
| *CANT1* |  |
| *TAP1* |  |
|  |  |
| **Downregulated HMGs also low in HLE cells (gene and or protein)** | |
| *SARDH* | detected as low by proteomics |
| *HNMT* | detected as low by proteomics |
| *NNT* | detected as low by proteomics |
| *ABCD3* | detected as low by proteomics |
| *ACAA1* | detected as low by proteomics |
| *ADI1* | detected as low by proteomics |
| *ARG1* | detected as low by proteomics |
| *ASL* | detected as low by proteomics |
| *CBS* | detected as low by proteomics |
| *GRHPR* | detected as low by proteomics |
| *PC* | detected as low by proteomics |
| *UGP2* | detected as low by proteomics |
| *EHHADH* | detected as low by proteomics |
| *ENPP1* | detected as low by proteomics |
| *HGD* | detected as low by proteomics |
| *MTTP* | detected as low by proteomics |
| *SLC39A14* | detected as low by proteomics |
| *SULT2A1* | detected as low by proteomics |
| *TF* | detected as low by proteomics |
| *GCAT* | detected as low by proteomics |
| *GLUD1* | detected as low by proteomics |
| *ACAA2* | detected as low by proteomics |
| *ACADSB* | detected as low by proteomics |
| *HADH* | detected as low by proteomics |
| *ALDH2* | detected as low by proteomics |
| *ALDH1B1* | detected as low by proteomics |
| *ABHD10* | detected as low by proteomics |
| *QDPR* | detected as low by proteomics |
| *AGL* | detected as low by proteomics |
| *GAMT* | detected as low by proteomics |
| *A1CF* | detected as low by proteomics |
| *GATM* | detected as low by proteomics |
| *GLDC* | detected as low by proteomics |
| *CA2* | detected as low by proteomics |
| *CPOX* | detected as low by proteomics |
| *PSAT1* | detected as low by proteomics |
| ***PAH*** | detected as low by proteomics |
| ***ACOX1*** | detected as low by proteomics |
| ***CAT*** | detected as low by proteomics |
| ***PCK2*** | detected as low by proteomics |
| ***PGM1*** | detected as low by proteomics |
| ***QPRT*** | detected as low by proteomics |
| ***SORD*** | detected as low by proteomics |
| ***ACO1*** | detected as low by proteomics |
| ***DAK*** | detected as low by proteomics |
| ***GOT1*** | detected as low by proteomics |
| ***PHGDH*** | detected as low by proteomics |
| *ASS1* |  |
| *THNSL1* |  |
| *CPT2* |  |
| *ABAT* |  |
| *ADH1B* |  |
| *ALDH6A1* |  |
| *ALDH8A1* |  |
| *CDO1* |  |
| *CRAT* |  |
| *CYP1A1* |  |
| *CYP2C9* |  |
| *CYP4F12* |  |
| *CYP4F2* |  |
| *DBT* |  |
| *GSTA1* |  |
| *LCAT* |  |
| *LIPC* |  |
| *LIPG* |  |
| *PON3* |  |
| *SLC38A4* |  |
| *SLC7A2* |  |
| *ST3GAL6* |  |
| *ST6GAL1* |  |
| *UPB1* |  |
| *ACADVL* |  |
| *ACOX2* |  |
| *ALDH4A1* |  |
| *BHMT* |  |
| *CPS1* |  |
| *CYP39A1* |  |
| *HPD* |  |
| *KHK* |  |
| *PCCA* |  |
| *PIK3R1* |  |
| *PON1* |  |
| *SLC23A2* |  |
| *SLC2A2* |  |
| *SLC31A1* |  |
| *SLCO1B3* |  |
| *CTH* |  |
| *ACADL* |  |
| *MLYCD* |  |
| *BCKDHB* |  |
| *BHMT2* |  |
| *ECHDC3* |  |
| *LGSN* |  |
| *PCYOX1* |  |
| *HMGCS2* |  |
| *EPHX2* |  |
| *PHYH* |  |
| *SCP2* |  |
| *MGST2* |  |
| *MAN1C1* |  |
| *GBA3* |  |
| *HYAL1* |  |
| *KCNJ8* |  |
| *BDH2* |  |
| *LPIN2* |  |
| *ADH6* |  |
| *ABHD2* |  |
| *CA5A* |  |
| *TGDS* |  |
| *DHODH* |  |
| *DCXR* |  |
| *PCCB* |  |
| *RBKS* |  |
| *FXN* |  |
| *GCH1* |  |
| *PAPSS2* |  |
| *DHRS1* |  |
| *SLC1A1* |  |
| *SLC25A15* |  |
| *SLC25A20* |  |
| *SLC27A2* |  |
| *SLC47A1* |  |
| *SLCO2B1* |  |
| *SLC4A4* |  |
| *SLC17A1* |  |
| *SLC17A2* |  |
| *SLC37A4* |  |
| *GCDH* |  |
| *PIPOX* |  |
| *FAHD2A* |  |
| *BCKDHA* |  |
| *HAL* |  |
| *TDO2* |  |
| *ELOVL6* |  |
| *ENO3* |  |
| *MPC1* |  |
| *KCNAB1* |  |
| *KCNJ16* |  |
| *AGPAT2* |  |
| *HPGD* |  |
| *SULT1A1* |  |
| *CP* |  |
| *AQP3* |  |
| *NPC1L1* |  |
| *SLC16A10* |  |
| *SLC19A3* |  |
| *AKR1D1* |  |
| *HSD17B2* |  |
| **HUH7 data continues next page.** | |

| **Upregulated HMGs also high in HUH7 cells (gene and or protein)** | |
| --- | --- |
| *CKB* | detected as high by proteomics |
| *AGPS* | detected as high by proteomics |
| *PSPH* | detected as high by proteomics |
| *GFPT1* | detected as high by proteomics |
| *SEPHS1* | detected as high by proteomics |
| *ATP1B1* | detected as high by proteomics |
| *PYCR1* | detected as high by proteomics |
| *MDH2* | detected as high by proteomics |
| *ME1* | detected as high by proteomics |
| *DLAT* | detected as high by proteomics |
| *ACSL3* | detected as high by proteomics |
| *ALDH18A1* | detected as high by proteomics |
| *FDPS* | detected as high by proteomics |
| *ACBD3* | detected as high by proteomics |
| *HK2* | detected as high by proteomics |
| *IDI1* | detected as high by proteomics |
| *NANS* | detected as high by proteomics |
| *PGD* | detected as high by proteomics |
| *ASNS* | detected as high by proteomics |
| *PFAS* | detected as high by proteomics |
| ***IDH3B*** | detected as high by proteomics |
| ***DDAH2*** | detected as high by proteomics |
| ***BCAT1*** | detected as high by proteomics |
| ***ADSS*** | detected as high by proteomics |
| ***ADSL*** | detected as high by proteomics |
| ***SRM*** | detected as high by proteomics |
| ***NQO1*** | detected as high by proteomics |
| ***ABCF1*** | detected as high by proteomics |
| ***TXNRD1*** | detected as high by proteomics |
| ***PRDX1*** | detected as high by proteomics |
| ***ACACA*** | detected as high by proteomics |
| ***GPI*** | detected as high by proteomics |
| ***ALDOA*** | detected as high by proteomics |
| ***ACSL4*** | detected as high by proteomics |
| ***UGDH*** | detected as high by proteomics |
| ***ATIC*** | detected as high by proteomics |
| ***GAPDH*** | detected as high by proteomics |
| ***ACLY*** | detected as high by proteomics |
| ***PKM*** | detected as high by proteomics |
| ***FASN*** | detected as high by proteomics |
| *LYZ* |  |
| *ENPP2* |  |
| *SULT1C2* |  |
| *CA12* |  |
| *PLCB1* |  |
| *MPC2* |  |
| *CHKA* |  |
| *GPX7* |  |
| *PLA2G7* |  |
| *SLC29A2* |  |
| *B3GALNT1* |  |
| *CDS1* |  |
| *PLA2G4C* |  |
| *GGPS1* |  |
| *GPD1L* |  |
| *SLC38A6* |  |
| *PDXK* |  |
| *AKR1B10* |  |
| *SCD* |  |
| *GNPAT* |  |
| *ATP8B2* |  |
| *SLC1A4* |  |
| *COX5A* |  |
| *STARD7* |  |
| *TMCO3* |  |
| *SYNJ2* |  |
| *SLC7A6* |  |
| *NPL* |  |
| *SQLE* |  |
| *AACS* |  |
| *SLC2A6* |  |
| *UQCRB* |  |
| *SLCO2A1* |  |
| *PON2* |  |
| *NT5DC2* |  |
| *BPGM* |  |
| *GMDS* |  |
| *P2RX4* |  |
| *ME2* |  |
| *ABCC5* |  |
| *TSTA3* |  |
| *CYCS* |  |
| *SLC33A1* |  |
| *ATP1A1* |  |
| *AGK* |  |
| *CHPF2* |  |
| *SLC1A3* |  |
| *IP6K2* |  |
| *SLC26A6* |  |
| *ALG3* |  |
| *FLAD1* |  |
| *SLC39A1* |  |
| *NOX4* |  |
| *DOLK* |  |
| *GUK1* |  |
| *ABHD4* |  |
| *ATP6V1E1* |  |
| *PAPSS1* |  |
|  |  |
| **Downregulated HMGs also low in HUH7 cells (gene and or protein)** | |
| **HMGCL** | detected as low by proteomics |
| **COMT** | detected as low by proteomics |
| **ACADVL** | detected as low by proteomics |
| **SDHA** | detected as low by proteomics |
| **GOT2** | detected as low by proteomics |
| **PCYOX1** | detected as low by proteomics |
| **SFXN1** | detected as low by proteomics |
| **ACADM** | detected as low by proteomics |
| **ALDH7A1** | detected as low by proteomics |
| ACAT1 | detected as low by proteomics |
| NNMT | detected as low by proteomics |
| SUCLG2 | detected as low by proteomics |
| SOD1 | detected as low by proteomics |
| ADK | detected as low by proteomics |
| MTHFD1 | detected as low by proteomics |
| MUT |  |
| ACSL5 |  |
| DBH |  |
| MME |  |
| PEMT |  |
| SLC38A2 |  |
| BCHE |  |
| SDHB |  |
| GPHN |  |
| ETFDH |  |
| CTBS |  |
| PIGV |  |
| BDH1 |  |
| CBR4 |  |
| UAP1 |  |
| AOX1 |  |
| SLC27A5 |  |
| SLC25A37 |  |
| HSD17B6 |  |
| KYNU |  |
| MGLL |  |
| CHST4 |  |
| KCNMA1 |  |
| KCNN2 |  |
| DSE |  |
| KDSR |  |
| CYP2E1 |  |
